# Collapse of the mammoth-steppe in central Yukon as revealed by ancient environmental DNA

**DOI:** 10.1038/s41467-021-27439-6

**Published:** 2021-12-08

**Authors:** Tyler J. Murchie, Alistair J. Monteath, Matthew E. Mahony, George S. Long, Scott Cocker, Tara Sadoway, Emil Karpinski, Grant Zazula, Ross D. E. MacPhee, Duane Froese, Hendrik N. Poinar

**Affiliations:** 1 McMaster Ancient DNA Centre, McMaster University, Hamilton, Canada; 2 Department of Anthropology, McMaster University, Hamilton, Canada; 3 Department of Earth and Atmospheric Sciences, University of Alberta, Edmonton, Canada; 4 School of Geography and Environmental Science, University of Southampton, Southampton, United Kingdom; 5 Department of Biology, McMaster University, Hamilton, Canada; 6 The Hospital for Sick Children, Toronto, Canada; 7 Yukon Government, Palaeontology Program, Department of Tourism and Culture, Whitehorse, Canada; 8 Collections and Research, Canadian Museum of Nature, Ottawa, Canada; 9 Division of Vertebrate Zoology/Mammalogy, American Museum of Natural History, New York, United States; 10 Department of Biochemistry, McMaster University, Hamilton, Canada; 11 Michael G. DeGroote Institute for Infectious Disease Research, McMaster University, Hamilton, Canada; 12 CIFAR Humans and the Microbiome Program, Toronto, Canada

**Keywords:** Molecular ecology, Archaeology, Palaeontology, Eukaryote

## Abstract

The temporal and spatial coarseness of megafaunal fossil records complicates attempts to to disentangle the relative impacts of climate change, ecosystem restructuring, and human activities associated with the Late Quaternary extinctions. Advances in the extraction and identification of ancient DNA that was shed into the environment and preserved for millennia in sediment now provides a way to augment discontinuous palaeontological assemblages. Here, we present a 30,000-year sedimentary ancient DNA (sedaDNA) record derived from loessal permafrost silts in the Klondike region of Yukon, Canada. We observe a substantial turnover in ecosystem composition between 13,500 and 10,000 calendar years ago with the rise of woody shrubs and the disappearance of the mammoth-steppe (steppe-tundra) ecosystem. We also identify a lingering signal of *Equus* sp. (North American horse) and *Mammuthus primigenius* (woolly mammoth) at multiple sites persisting thousands of years after their supposed extinction from the fossil record.

## Abstract (web summary)

‘The timing and ecological dynamics of extinction in the late Pleistocene are not well understood. Here, the authors use sediment ancient DNA from permafrost cores to reconstruct the paleoecology of the central Yukon, finding a substantial turnover in ecosystem composition between 13,500-10,000 years BP and persistence of some species past their supposed extinctions.’

## Introduction

Humans evolved and dispersed throughout the continents in an epoch dominated by giant terrestrial mammals. Megafauna (body mass ≥ 44 kg) only exist in comparable densities today within small refugia (mainly Africa) where most of their populations are in states of decline, and many of these species are threatened or endangered^1,2^. The ecological reverberations associated with the Late Pleistocene (130,000–11,700 years before present [BP]) loss of approximately 101 of 150 genera^3^ of Earth’s largest terrestrial animals is thought to have restructured the terrestrial biosphere, impacting vegetation composition and diversity, biogeochemistry, and climate feedback systems^4–13^. This rearrangement of terrestrial ecosystems, including massive biogeographic range shifts, local extirpations, and widespread extinctions, is argued by some to be the direct result of rapid climate change and attendant environmental feedbacks during the late Pleistocene^14–17^. Others contend^18–23^ that factors unique to the last glacial period are to blame, such as the coincident dispersal of a new predator—*Homo sapiens*. It is likely that no single factor can account for the staggered magnitude of such losses globally, but rather that each ecosystem experienced a variable set of locally compounding pressures^17,24,25^. Taphonomic processes challenge attempts to tease apart the palaeoecological nuances of the late Quaternary extinctions (LQE), necessitating relatively precise estimates for megafaunal population declines and last appearance dates^26,27^, for timings of ecological shifts (e.g. changes in plant community structure), as well as for robust archaeological evidence of anthropogenic impacts.

In the case of eastern Beringia (unglaciated regions of Yukon, Canada and Alaska, U.S.A.), Guthrie^16^, Mann et al.^28,29^, and Rabanus-Wallace et al.^30^ argue that the expansion of woody shrubs and peatlands following an increased moisture regime during the late Pleistocene was the leading contributor to the loss of megafaunal grazers, including mammoth, horse, and bison. By contrast, Zimov et al.^23,31^ contend that megafaunal extirpations preceded a rise in woody shrubs, with the loss of keystone megaherbivores having led to the disappearance of the graminoid and forb dominated, mammoth-steppe biome^5,31–33^. Disentangling the relative timings of ecological restructuring versus megafaunal population declines often exceeds the resolution^34^ of Quaternary records.

**Fig. 1:**
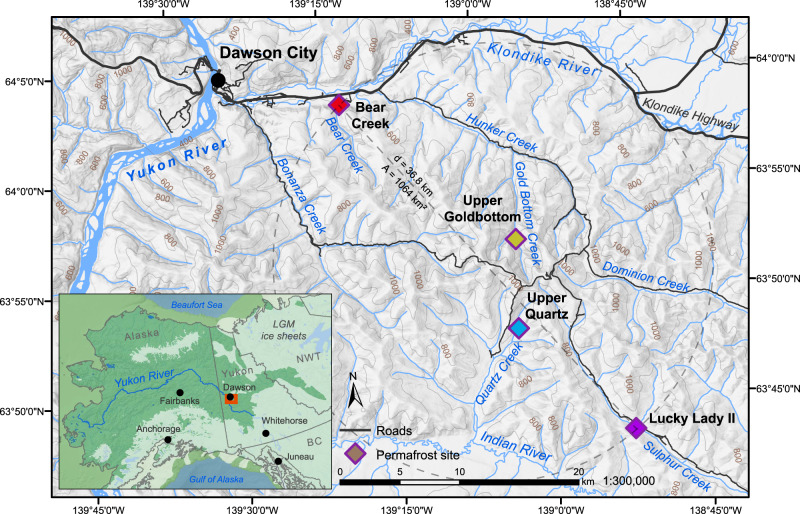
Permafrost sites from the Klondike region of Yukon, Canada. Base map data retrieved from GeoYukon (hosted by the Government of Yukon); contours elevation unit: meters above sea level. See Supplementary Fig. 2 for a description of inset map data sources.

Here, we present hybridization capture enriched sedimentary ancient DNA (sedaDNA) data derived from loessal silts preserved in permafrost (Fig. 1, Table 1) and recovered from four sites in the Klondike goldfields—an unglaciated region of west-central Yukon Territory, Canada^35^—dating to ca. 30,000–4000 calibrated (calendar) years before present (cal BP). This work builds on the methodological results reported in Murchie et al.^36^ in which North American horse (*Equus caballus*) and woolly mammoth (*Mammuthus primigenius)* DNA was unexpectedly identified in a permafrost sample dating to ~9700 cal BP. This post-dates the last macrofossil evidence (such as bones, teeth, and soft-tissues) of these animals in Alaska by some 3300 years. Such a late date is indicative of a substantial ghost range (cryptic population)—an extended spatio-temporal range derived from palaeoecological proxies that post-date the last macrofossil remains^37^.

**Table 1:** SedaDNA permafrost samples.

Site	Core^a^	ID^b^	Calibrated age-model^c^ (2σ)			Input^d^	Total raw reads	PalaeoChip mapped & *MEGAN* assigned^e^
			Median	From	To			
Bear Creek	BC 4-2B	PHP-1	≳30,000			1.05	5,650,809	241,143 (4.27%)
Lucky Lady II	LL2S-189-E	PHP-2	8707	8919	8637	0.9	8,667,496	9,908 (0.11%)
	LL2S-253-D1	PHP-3	9177	9485	8950	0.6	9,303,528	491,487 (5.28%)
	LL2C-118-C	PHP-4	13,176	13,250	13,119	0.6	7,749,177	178,973 (2.31%)
	LLII 12-84-3	PHP-6	13,335	13,393	13,277	0.6	10,235,721	806, 601 (7.88%)
	LL2C-205-B	PHP-5	13,510	13,596	13,401	1.35	4,997,824	48,226 (0.96%)
	LL2C-243-A2	PHP-7	14,113	14,245	13,975	0.6	5,820,472	54,700 (0.94%)
	LLII 12-170-6	PHP-8	14,750	14,897	14,595	0.3	1,331,181	17,628 (1.32%)
	LLII 12-217-8	PHP-9	15,500	15,651	15,339	1.05	3,115,296	38, 566 (1.24%)
Upper Goldbottom	MM12-118B	PHP-11	9,246	9869	8604	1.35	7,983,085	541,521 (6.78%)
	MM12-119B	PHP-12	10,051	10,181	9909	1.5	18,409,441	1,823,618 (9.91%)
	MM12-115b	PHP-14	19,704	20,320	19,070	0.3	2,320,965	35,539 (1.53%)
	MM12-116b	PHP-13	20,787	20,955	20,554	0.3	1,624,580	174,890 (10.77%)
	MM12-117b	PHP-15	21,232	22,424	20,568	0.3	1,910,387	164,997 (8.64%)
Upper Quartz	MM12-QC-10	PHP-19	3849	3899	3727	0.6	9,244,928	190,555 (2.06%)
	MM12-QC-9	PHP-20	5776	5929	4590	0.6	5,913,447	312,597 (5.29%)
	MM12-QC-8	PHP-21	5909	5992	5744	0.6	5,142,454	265889 (5.17%)
	MM12-QC-6	PHP-22	12,860	12,989	12,758	0.6	6,080,182	114,978 (1.89%)
	MM12-QC-4	PHP-23	14,939	15,958	13,988	0.9	14,729,646	68,186 (0.46%)
	MM12-QC-3	PHP-24	15,759	16,862	14,720	0.3	1,129,623	17,162 (1.52%)
	MM12-QC-2	PHP-25	16,563	17,810	15,469	0.3	1,568,411	29,419 (1.88%)
Blanks	Extraction (9) & library (4) negative controls						102,440	81 (0.08%)
	TOTAL						132,928,653	5,626,583 (4.23%)

Core identifications as per D’Costa et al.^106^ for Bear Creek, Mahony^100^ for Lucky Lady II, Upper Goldbottom, and Upper Quartz, and Sadoway^109^ for Lucky Lady II.

^a^Lucky Lady II subsamples from vertical cores. Bear Creek, Upper Goldbottom, and Upper Quartz are horizontal samples taken from permafrost exposures.

^b^PHP = Pleistocene-Holocene Permafrost sedaDNA ID. See Supplementary Table 14 for a breakdown of sedaDNA replicates by core.

^c^See Supplementary Figs. 5–7 for Bayesian age-models. Dates reported as calendar/calibrated years before present (cal BP).

^d^Sediment input across extraction replicates.

^e^Sequenced reads that pass quality filters for de-duplication, adaptemer removal, minimum size (24 bp), and map to the PalaeoChip references^36^ that could then also be *BLASTn* aligned and taxonomically binned in *MEGAN*. Percent on-target reads (those that map to the PalaeoChip references) of total raw sequenced reads.

Our aims here are two-fold. The first is to assess how our permafrost sedaDNA dataset can be used to assess the plausibility of two competing explanatory models of Beringian extinctions. These are the bottom-up “shrub/peatland expansion” model of Guthrie^16^ and Mann et al.^28–30^, and the top-down “keystone megaherbivore decline” model of Zimov et al.^5,23^. The shrub/peatland expansion model argues that climate change drove ecosystems into new equilibrium states that were unfavourable for megafaunal herbivores in eastern Beringia^25^. In contrast, the keystone-decline model posits that megaherbivores were active ecological engineers. When their populations began to decline (from anthropogenic or environmental pressures), it triggered numerous knock-on effects including the disappearance of mammoth-steppe vegetation and its replacement by shrublands and wet tundra. Our second objective is to replicate and test the extent of the sedaDNA ghost ranges for *Equus* sp. and *Mammuthus* as identified in Murchie et al.^36^.

## Background

### Late Quaternary losses on the eastern mammoth-steppe

Megafaunal extinctions and extirpations after 40,000 cal BP in the Holarctic (i.e., northern Eurasia, Beringia, and North America) arguably followed a two-stage pattern suggested by dated macrofossils^27^ (Supplementary Table 1). The first wave seems to have occurred prior to or during the Last Glacial Maximum (LGM, 26,500–19,000 cal BP)^38^ which in eastern Beringia included the loss of *Homotherium latidens* (scimitar-toothed cat) and *Arctodus simus* (short-faced bear). The second wave occurred between 15,000–10,000 cal BP, which in mainland Beringia included Equidae (caballine and stilt-legged horses), *Mammuthus primigenius* (woolly mammoth), *Panthera spelaea* (cave lion), and *Saiga tatarica* (saiga antelope), while at least 20 genera went extinct or were extirpated in North America south of the continental ice sheets. Other species such as *Bison priscus* (steppe bison) survived until at least ~6000 years ago in southern Yukon^39^, with genetic data supporting a *B. priscus* persistence until as recently as a few hundred years ago^40^. *M. primigenius* survived on Wrangel (Russia) and St. Paul (Alaska) islands until about 4000 and 5500 cal BP, respectively^41,42^. Holarctic faunal assemblages of Pleistocene predators, species with small populations, and taxa living in understudied regions are expectedly sparse. This has led some to argue that these two late Pleistocene extinction/extirpation pulses are an artifact of an inadequate record and the Signor-Lipps effect^43,44^, and that the true wave of losses occurred during or after the Younger Dryas, ca. 12,900 cal BP^25,37,45^.

Beringia’s environment during the late Pleistocene has been characterized as a graminoid and forb-dominated steppe-tundra mosaic generally referred to as the mammoth-steppe^31,33,35,46–50^. It is thought to have been the most extensive terrestrial biome on Earth during the late Pleistocene, stretching from the Iberian Peninsula eastward across Eurasia and into Canada^31,50–52^, although the extent and character of this ecosystem remains controversial for some^53^. This paradoxically productive^54,55^ high-latitude mosaic biome supported a diverse abundance of large bodied fauna^31,50,52^, facilitating higher biotic productivity (energy and nutrient turnover) than many habitats existing at high latitudes today^23,56^.

Owen-Smith^32^ proposed the keystone herbivore hypothesis based on the ecology of extant African megafauna to explain the role of megaherbivores in transforming vegetation structure and composition^57–63^. Nutrients locked in leaves and stems are liberated when used by fauna, accelerating biogeochemical cycling^11^. In this top-down model, megafauna are critical for maintaining and promoting biodiversity in open-mosaic environments, and for controlling the abundance of woody vegetation that can limit biodiversity^5^. A severe reduction of these megafaunal engineers^64–66^ is proposed to have resulted in the conversion of mosaic, steppe grasslands and wood pastures to more uniform forests and prairies, shrinking mosaic ecotones—high productivity transition areas between biological communities^67^—thereby reducing the carrying capacity of terminal Pleistocene environments. This in turn could have led to a positive-feedback response wherein diminishing populations of megafauna were increasingly unable to control woody shrub expansion, further reducing the biotic productivity of the mammoth-steppe.

Alternatively, an increasing moisture regime during the Bølling–Allerød interstadial (ca. 14,690–12,890 cal BP)^68^ is argued to have caused the rise of mesic-adapted woody shrubs that were highly defended against herbivory, replacing the diet of Pleistocene grazers (woolly mammoth, steppe bison, and horse)^16,28–30^, along with the paludification of Beringia (the spread of peatlands). In this line of bottom-up arguments, climate change and attendant environmental feedbacks led to the disappearance of the mammoth-steppe in eastern Beringia, along with the megafauna it supported.

### Power and limits of sedimentary ancient DNA

Much of the LQE debate has been limited by the inability of dated macrofossils (primarily from detrital contexts) to convey the spatio-temporal resolution necessary to untangle the causative versus correlative ecological transformations associated with the Pleistocene-Holocene transition. Molecular (micro) methods^69^ are increasingly able to augment discontinuous macrofossil records. This can aid in identifying cryptic populations (or ghost ranges)^37,70,71^, independently assess population declines, and estimate the timings of functional extinctions/extripations^15,72^—the point at which undercrowding and inbreeding depression lead to a loss of fitness through Allee effects^73–75^ to the degree that a species no longer significantly contributes to ecosystem functioning, becoming a trace presence in records before completely disappearing.

Ancient environmental DNA (eDNA)^76^ is a powerful method for directly assessing the local^77^ presence of animals, plants, fungi, and microbiota through time^33,36,37,41,78–82^. Most eDNA is quickly metabolized by bacteria or otherwise degraded through a variety of chemical and physical processes. SedaDNA (referring to a subset of ancient eDNA sample types^83^) can survive these degradative processes, even in the absence of visible fossils, because cellular material can bind to sedimentary minerals^84–91^, protecting these molecular fragments for millennia, especially when perennially frozen^92,93^. Sediment samples as small as 100 mg can contain tens of billions of DNA fragments from all forms of life in a local ecosystem. However, there are several sedaDNA challenges to be aware of: (1) determining whether the recovered sedaDNA is stratigraphically accurate^91^; (2) whether the wet-lab recovery and targeting strategy, genetic reference databases, and taxon assignment approach (bioinformatic parameters) can accurately assess the breadth of eDNA or is prone to false-positive/negative assignments^36,94^; (3) assessing whether eDNA abundance retains a correlation with a population’s living biomass^95,96^; and (4) determining the degree to which sedimentary inhibitors or differential degradation may bias the sedaDNA signal^36^. Of particular importance for the kinds of permafrost sedaDNA analyzed in this report are the factors of reworked sedaDNA and leaching.

Perennially frozen sedaDNA has the potential to undergo erosion and redeposition while remaining chemically intact. Arnold et al.^91^ found evidence of reworked periglacial sediments in high-energy fluvial contexts within large catchments and local thermokarst deposists. They caution against sampling from settings where DNA can be readily reworked and redeposited within younger materials. In this study, we targeted loessal silts to mitigate the potential for fluvial reworking, although aeolian processes are certainly capable of reworking nanoscale sedaDNA complexes. Further, there is evidence throughout the Klondike of an early Holocene thaw unconformity^97–99^ that Mahony^100^ identified at Upper Goldbottom and Upper Quartz (among other sites) that presents the possibility for the localized reworking of sedaDNA during our early Holocene core samples. The Lucky Lady II section by contrast is continuous from >16,500– 8500 cal BP, sits toward the middle of a broad valley, and shows no evidence of erosion or redeposition by slope wash or thermokarst-induced slumping—suggesting that reworked early Holocene sedaDNA is of less concern at the Lucky Lady II site.

The vertical movement of free DNA has been found to be negligible in perennial frozen settings^91^. Studies have found synchronous palaeoecological shifts when comparing palynological and macrofossil evidence with sedaDNA reconstructions^101,102^, in addition to age-dependent DNA damage patterns^103–105^. Leaching is not considered to be a problem in perennially frozen sediment because there is minimal movement of liquid water^33,37,102,106–108^.

## Results

### Age-modelling and palynology

Age-depth models were first reported for cores used in this study by Sadoway^109^ and Mahony^100^. To refine the chronologies of these records we developed Bayesian age-depth models for each site using Oxcal v.4.4.2^110^ and the IntCal20 calibration curve^111^ (Supplementary Figs. 5–7 and Supplementary Table 3). Some outlier dates from the model were observed (Supplementary Figs. 6–7). As such, remodelled ages are reported here as 2σ maximum/minimum ranges to account for this uncertainty for comparing samples within and between sites (Table 1). A selection of subsamples were also analysed with palynology as a secondary proxy to the eDNA data. Most samples were nearly barren of palynomorphs, with only the Holocene-aged samples having preserved large numbers of pollen grains—in this case predominantly spruce (*Picea*) (Supplementary Table 4).

### SedaDNA palaeoecology

**Fig. 2:**
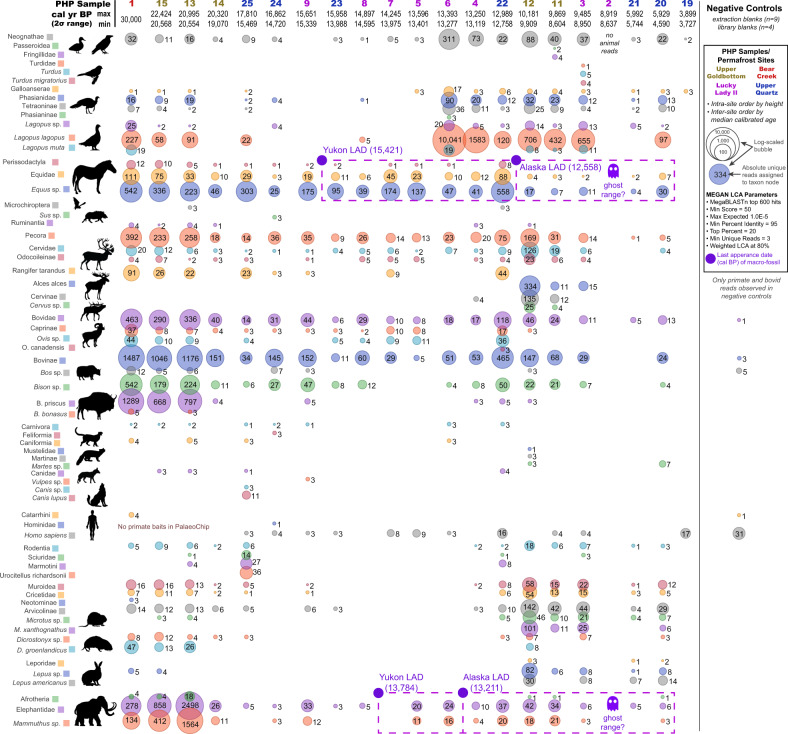
Metagenomic comparison of animal reads assigned using BLASTn to MEGAN. Values indicate unique reads assigned to that taxon node. Source data are provided as a Source Data file.

Through the targeted capture^36^ of organelle eDNA preserved in loessal permafrost silts (Supplementary Fig. 4), *Bison priscus* (steppe bison), *Mammuthus primigenius* (woolly mammoth), *Equus* sp. (specifically limited to caballine horse), and *Lagopus lagopus* (willow ptarmigan) constitute most of the identifiable DNA reads of direct interest within Animalia (Fig. 2). This is in addition to less abundant organisms such as *Rangifer tarandus* (caribou/reindeer), and *Ovis sp*. (likely Dall sheep). Many reads expectedly lack taxonomic specificity at species and genus ranks (as many regions of the mitochondrial genome are variably conserved), and as such a large portion of reads could only be confidently assigned to higher ranks such as Caprinae, Pecora, Perissodactyla, and Elephantidae. In some cases, such as with hits to order Perissodactyla and superorder Afrotheria, we can be confident that they represent *Equus* (or its familial relative, *Haringtonhippus* [stilt-legged horse]), and *Mammuthus*, respectively, as unique members of their clades in the late Quaternary record of this region. There is also a low biomolecular signal from predators in this dataset including *Canis lupus* (grey wolf) and *Martes* sp. (marten). A variety of rodents were identified, including *Urocitellus* sp. (likely arctic ground squirrel), *Microtus xanthognathus* (taiga vole), and *Dicrostonyx groenlandicus* (northern collared lemming). Human DNA was identified despite not being targeted with the PalaeoChip baits. However, human DNA was also observed in the negative controls. We do not consider this human signal to be reliable without further investigation.

**Fig. 3:**
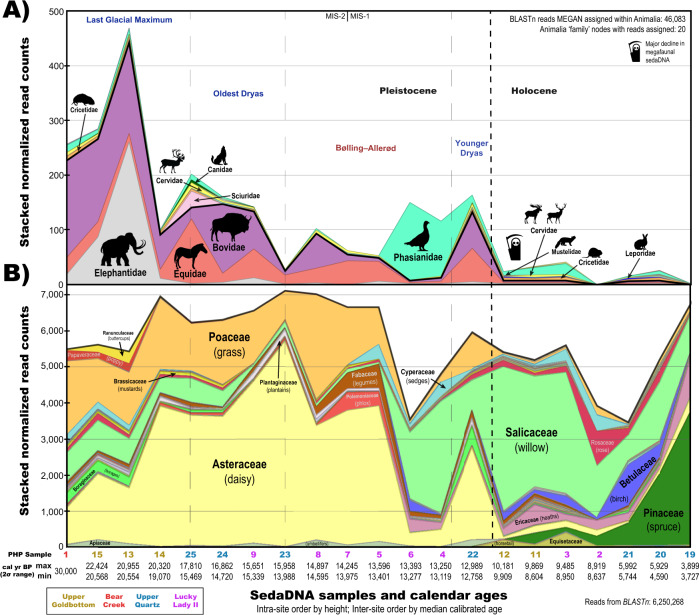
Major taxonomic shifts in relative sedaDNA read assignment through time. Stacked normalized reads assigned to the rank ‘family’ in (**A**) Animalia (insects excluded) and (**B**) Viridiplantae. See Supplemental Figs. 10–12 for a metagenomic breakdown of samples by site. Source data are provided as a Source Data file. Note: sedaDNA abundance is likely affected by variable taphonomic intensity through time (such as temperature, moisture, and acidity) among other known and unknown micro-environmental conditions and recovery biases. While eDNA abundance and biomass arguably retain some correlation upon initial release, many other factors influence this relationship. As such, relative abundances with sedaDNA must be interpreted cautiously pending further research.

Overall, this taxonomically rich sedaDNA dataset reflects a gradual decline in the megafauna signal through time (Fig. 3). Elephantidae is one of the first to decrease in sedaDNA abundance after ~20,000 cal BP. This is followed by declining signals for Bovidae and Equidae (Fig. 2), until a punctuated decrease occurs near the Pleistocene-Holocene transition as their sedaDNA signals almost disappear while those for *Alces alces* (moose) and *Cervus sp*. (likely *Cervus canadensis* [elk/wapiti]) enter the dataset. Our admittedly temporally coarse set of permafrost samples suggests a delay in the disappearance of megafaunal grazing species in the Klondike to between ca. 13,000–10,000 cal BP, but we also observe a lag in the final appearance of *Equus* and *Mammuthus* sedaDNA as late as ca. 6000 cal BP. Cores from Lucky Lady II, Upper Goldbottom, and Upper Quartz retain 100+ DNA sequences assignable to those taxa well beyond their last dated macrofossils (Fig. 2).

**Fig. 4:**
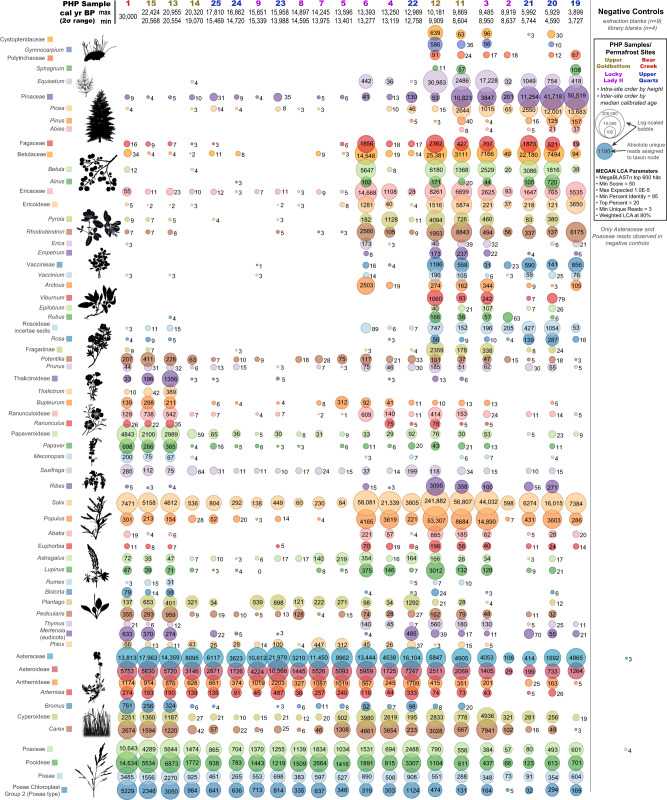
Viridiplantae metagenomic comparison of permafrost core subsamples analyzed using *BLAST*n to *MEGAN* assigned reads. Values indicate unique reads assigned to that taxon node. Select taxon nodes depicted. Source data are provided as a Source Data file.

Plant sedaDNA clearly reflects a major environmental turnover between 13,500–10,000 cal BP (Figs. 3–4). Pleistocene graminoids (grass-like, herbaceous [non-woody] plants) such as Poaceae (grasses), Cyperaceae (sedges), along with a variety of forbs (herbaceous flowering plants) such as *Artemesia* (sagebrush), *Lupinus* (lupine), *Saxifraga* (rockfoil), *Papaver* (poppy), and *Ranunculus* (buttercup) were identified in relative sedaDNA abundance from 30,000–13,500 cal BP. Woody taxa such as *Salix* (willow), *Populus* (poplar), *Betula* (birch), *Rhododendron*, *Arctous* (bearberry), and *Picea* (spruce) were identified with increasing relative sedaDNA abundances after ~13,500 cal BP, along with *Equisetum* (horsetail), *Gymnocarpium* (oak ferns), and *Sphagnum* (peat moss). Our data suggests that forbs and graminoids (in this case Poaceae and Asteraceae) were dominant from ~30,000–13,500 cal BP while woody shrubs were comparatively rare (Fig. 3), indicating that the conventional idea of the mammoth-steppe holds until at least this late.

### Assessing aDNA authenticity

All 13 negative controls had negligible library adapted molecules prior to indexing (Supplementary Fig. 8) and had minuscule molarities after targeted enrichment (Supplementary Fig. 9). Despite using the entire post-enrichment eluate for each of the controls during equimolar pooling, these blanks received minimal sequenced reads (102,440) and even fewer reads that could be taxonomically binned (81, 0.08%). Shotgun sequencing blanks processed with a subset of these libraries in Murchie et al.^36^ were almost entirely (>95%) adaptemers (adapter chimeric DNA), and likewise contained no signal of the ecologically relevant organisms under investigation here or in that previous work. As all 13 negative controls were processed identically in parallel with the permafrost subsample replicates (Supplementary Figs. 34–54), and yet contain none of the same sedaDNA signal (Figs. 2, 4, Supplementary Fig. 55), we can conclude that the trends observed here originate from the sediments themselves and are not the result of contamination.

We observe ancient DNA characteristic damage profiles where sufficient reads could be mapped to MEGAN and PIA identified references (Fig. 5, Supplementary Figs. 13–28), confirming that these molecules are ancient and not modern contaminants. The taxonomic constituents of these samples are also correlated more with age than site (Fig. 6), with 90% of the variance being explained by principal components 1/2 as driven by the proportions of Pinaceae, Asteraceae, and Salicaceae. These libraries also exhibit age- and climate-related damage patterns (Supplementary Figs. 29–33), suggesting that reworking and leaching have contributed minimally (if at all) to the ecological reconstructions.

**Fig. 5:**
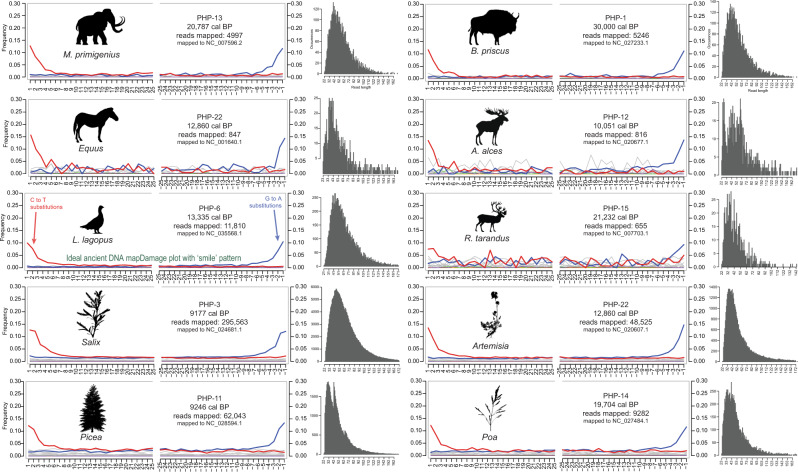
Example mapDamage plots. Minimum size 24 bp, minimum map quality 30. See Supplementary Figs. 13–28 for a full breakdown of fragment misincorporation plots and a discussion of on/off target mapping. Source data are provided as a Source Data file.

**Fig. 6:**
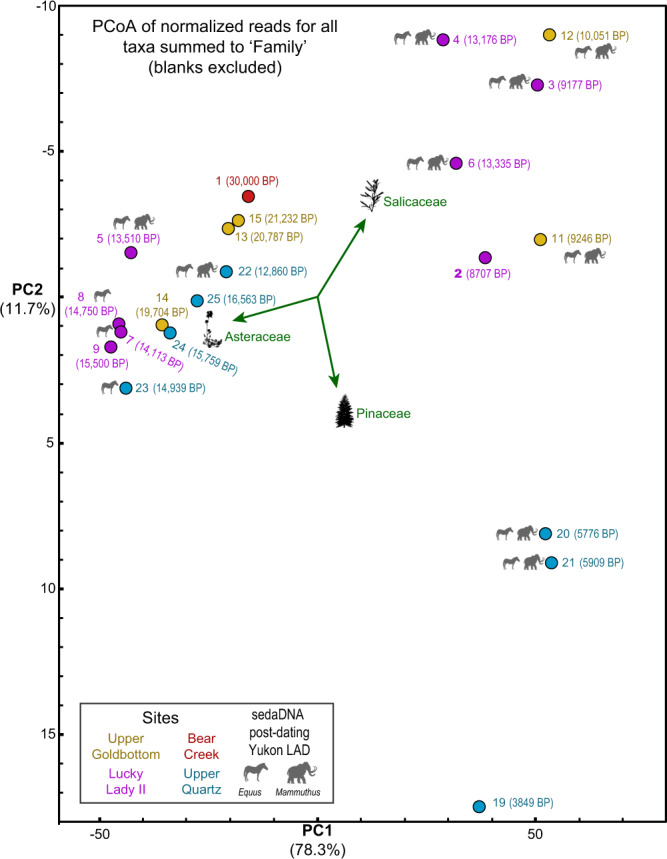
Metagenomic Principal Coordinates Analysis (PCoA) produced in MEGAN using a chi-square ecological index^187^. LAD last appearance date based on dated macrofossils (Fig. 2). Source data are provided as a Source Data file.

## Discussion

We observe four main trends within this dataset. (1) There is a surprising taxonomic richness and spatio-temporal consistency in the metagenomic signal across all sampling locations, suggesting that these reconstructions are representative of palaeoecological trends in the Klondike region. (2) Megafaunal sedaDNA declines gradually after the LGM with the *Mammuthus primigenius* signal being the first to drop out, followed by *Bison priscus* and *Equus*. (3) Signal dominance for forbs and graminoids is coeval with grazing megafauna, whereas the local transition towards woody shrubs is associated with a diminished faunal signal. Megafaunal sedaDNA reduces substantially after the Younger Dryas, by which time grazing megafauna had become functionally extirpated in the Klondike. (4) Despite this turnover, a low megafaunal sedaDNA signal persists into the Holocene. This signal—identified as a ghost range here—is suggestive of a late persistence of megafauna in a high latitude refugium, apparently outliving the functional extinction and complete loss of other continental populations.

### Ecological turnover and collapse of the mammoth-steppe

*Mammuthus, Equus*, and *Bison* presence are closely associated in our dataset with forbs and graminoids characteristic of the mammoth-steppe biome. During the relative sedaDNA rise in Salicaceae (likely willow shrubs) ca. 13,500 and 10,000 cal BP, Asteraceae and Poaceae correspondingly decline in relative sedaDNA abundance while grazing megafaunal DNA largely disappears from our dataset. This suggests that, at least in the Klondike, *Mammuthus primigenius* may have been the first megafaunal species to undergo a local population reduction after ~20,000 cal BP.

It is difficult to say whether sedaDNA signal decay reflects an actual reduction in the regional abundance of animals or is reflective of other stochastic and unknown^34^ factors unique to this proxy such as variable eDNA release and turnover^95^, biochemical changes to eDNA stabilization processes such as organo-mineral binding^90^, or shifting microbial and other taphonomic pressures. As *Equus* sedaDNA remains relatively consistent until the rise of mesic-hydric woody shrubs during the Allerød warming (~13,500 cal BP) (Fig. 3B), this is suggestive of longer-term local declines of *Mammuthus* (and perhaps *Bison*) eDNA input rather than a shifting of biomolecular taphonomy because we would generally expect animal eDNA to breakdown or become mineral-bound^88^ at similar local rates. Further, *Lagopus* DNA spikes during major declines in megafaunal DNA. Again, implying local ecological rather than just taphonomic or methodological factors driving shifts in relative animal sedaDNA recovery. As this study used a capture enrichment approach targeting organelle genomes^36^ (with replicates [Supplementary Table 14]) rather than PCR metabarcoding amplicons^76,112^, relative changes in metagenomic signal abundance are arguably somewhat correlated with shifting eDNA inputs^95,96,113–116^ during periods of otherwise stable climate. As such, we suggest that major shifts in relative DNA abundance, such as *Mammuthus* sedaDNA nearly dropping out of the dataset after 20,000 cal BP, are ecologically informative.

The decline in local mammoth abundance that we infer from this sedaDNA record is consistent with a low frequency of ^14^C-dated mammoth macrofossils from the Klondike. *M. primigenius* macrofossils are comparatively rare in central Yukon relative to more northerly sites around Old Crow (Supplementary Fig. 1), but still relatively few of those northern macrofossils have been successfully radiocarbon dated because most are beyond radiocarbon range^117,118^. Much of the <30,000-year fossil record in Beringia is represented by a small number of well-preserved, and logistically accessible sites (Supplementary Fig. 1). Our faunal sedaDNA dataset somewhat conflicts with these macrofossil abundances in that the highest frequencies of DNA reads identified as *Mammuthus*, *Equus*, or *Bison* are distributed between 30,000–15,000 cal BP. Conversely, dated faunal remains of *Mammuthus* and *Bison* in eastern Beringia have a median concentration around 15,000 cal BP, with only *Equus* bones being predominant nearer to 25,000 cal BP (Supplementary Fig. 1).

Relative *Bison* and *Equus* sedaDNA signals decrease in read counts after ~15,000 cal BP. However, there is a subsequent increase around the onset of the Younger Dryas (ca. 12,900 cal BP) that may be associated with previously described *Bison* dispersals^40,119^ or other local factors (e.g. shifting mosaic vegetation patches^25^, herbivore land use, or taphonomy). It is worth noting that this inference is limited by there only being a single Younger Dryas core sample in this dataset. After the Younger Dryas (during the early Holocene) grazer sedaDNA nearly disappears (Fig. 3), which is correlated with the ecological turnover from forbs and graminoids to woody shrubs—predominantly *Salix* sp.—and a rise in avian fauna, rodents, and cervid browsers. There is a pronounced transition in the ecological signal after ~13,500 cal BP from forbs and graminoids to woody shrubs in the Lucky Lady II cores, with rises in *Salix* reads tightly associated with a sharp increase of *Lagopus lagopus* (willow ptarmigan) sedaDNA—a grouse whose habitat and subsistence patterns are based on woody shrubs^120^ (Fig. 3). Keesing and Young^121^ observed on the African savanna that when large grazing mammals were removed from an area, the rodent populations doubled, which increased the populations of predators that target small-bodied animals. Our data mirrors this observation with an increase in rodent sedaDNA after ~10,000 cal BP (Fig. 2), along with the appearance of the small forest dwelling carnivore *Martes* sp. (martens) who may also now be present because of trees on the landscape.

After *Mammuthus primigenius* and *Equus* sp. were functionally extirpated from the Klondike, the local ecosystem began transitioning towards boreal taxa with an associated rise in *Picea* (spruce) and mosses (Fig. 4). Despite significant declines in grazing megafaunal DNA, reads extend beyond their last dated macro-remains—perhaps even as late as the mid-Holocene—which has already been observed for *Bison priscus*^39,40^.

Our plant dataset is consistent with Willerslev et al.^33^ in which forbs were found to proportionally dominate their metabarcoded sedaDNA signal during and after the LGM. Nichols et al.^112^ argue that the forb dominance observed in Willerslev et al.^33^ was partly caused by polymerase and GC biases of their PCR metabarcoding approach favoring forbs over graminoids with the Platinum HiFi Taq polymerase targeting the short *trnL* (P6 loop) locus^122^. We have used a capture enrichment approach (indexed and reamplified with the KAPA SYBR FAST qPCR Master Mix) targeting much larger regions of the chloroplast genome (*trnL* [~500 bp], *rbcL* [~600 bp], and *matK* [~800 bp])^36^ where overall GC content is generally equivalent between the three major target families identified in Fig. 3 (see Supplementary Fig. 56). We suspect that beyond the PCR biases argued to have influenced Willerslev et al.^33^ (i.e. the greater relative abundance of forb sedaDNA compared to graminoids and woody plants) that this is likely the result of eDNA release and preservation characteristics of forbs with higher rates of biomass turnover. This more rapid turnover thus potentially leads to an eDNA over-representation of forbs compared with typical palynological findings. It has been argued that interpreting relative floral abundances with eDNA requires calibration. Yoccoz et al.^95^, for example, observed that their above-ground vegetation surveys were accurately mirrored in modern environmental soil DNA, but that functional groups (woody plants, graminoids, and forbs) varied in their proportional eDNA representation. Woody plants were most affected by this trend, being proportionally under-represented in eDNA compared to above ground biomass by 1:5^95^, while graminoids were under-represented by 1:1.5. Conversely, forbs were over-represented by 2.5:1. GC and polymerase bias coupled with eDNA release variation, beyond simple growth form categories, complicates this further^112^. Nevertheless, the substantial abundance of forb DNA, even if cut by half, likely reflects an abundance of flowering herbs on the Pleistocene mammoth-steppe. This may also be under-represented palynologically due to varied pollen production between entomophilous (insect-pollinated) forbs and anemophilous (wind-pollinated) gramminoids^123^.

The rise in Pinaceae (notably spruce, see Fig. 4) around ~10,000 cal BP, and its growing dominance through our mid-Holocene samples, is consistent with other records from Yukon in regard to the initial development of the taiga/boreal forest^124–129^, and is consistent with pollen grains identified in samples younger than ca. 9200 cal BP (Supplementary Table 4). We observe similar relative sedaDNA increases in boreal flora (Fig. 4), albeit with a comparatively less abundant signal for *Betula*. Palynological studies frequently report an abundance of *Betula* with a comparatively small initial influx of *Salix* in the Alaskan-Yukon interior during the terminal Pleistocene shrub expansion^125^. While we observe a distinct rise in relative *Betula* sedaDNA during the Bølling–Allerød and post-Younger Dryas chronozones that persists into the Holocene, the number of *Betula* sedaDNA molecules are comparatively dwarfed by the immense abundances of Salicaceae (*Salix* [willow]) (Figs. 3–4) DNA. *Betula* is known to be over-represented by pollen, whereas *Salix* is often under-represented^130–133^. Our sedaDNA data suggests that *Salix* was more important in Beringian shrub expansion than palynological records have yet indicated.

The relative over-abundance of *Salix* sedaDNA compared to *Betula* is relevant to testing the shrub-expansion extinction model of Guthrie^16^, who contended that an increasing moisture regime and rise of mesic-hydric vegetation, with chemical defenses against herbivory (notably *Betula nana exilis* [resinous dwarf birch], but also including *Salix*)^134–136^, drove regional extirpations of grazing megafauna in eastern Beringia. If *Salix* was substantially more abundant in the Beringian shrub expansion than *Betula* as our sedaDNA dataset suggests, this questions whether the rise of defensive vegetation was a major driver in the extirpations as *Salix* is the most preferred and palatable shrub among extant subarctic browsers^135,137^. While *Bison* and *Equus* are considered closer towards the obligate grazer end of dietary guilds^50,138^, both have been observed to exhibit variable grazing and even mixed feeding^139,140^. *Mammuthus, Equus*, and *Bison* coprolites suggest that these taxa had a diet variably rich in forbs and graminoids, with a smaller but notable proportion of woody shrubs/trees (including alder [*Alnus*], birch [*Betula*], larch [*Larix*], spruce [*Picea*], and willow [*Salix*])^33,141,142^.

If mammalian sedaDNA abundances are rough indicators of palaeo-biomass (a correlation in need of further research), it is unclear why *Mammuthus primigenius* and *Bison priscus* (Fig. 2) relative sedaDNA signals decline prior to an expansion of woody shrubs during the Bølling-Allerød warming (Fig. 3). The abrupt increases in Cyperaceae (*Carex* [sedges]), Ericaceae (*Arctous* [bearberry], *Rhododendron*), Betulaceae, and Salicaceae during the Allerød are suggestive of a transition toward a moist dwarf-shrub ecosystem by ca. 13,500 cal BP. The presence of these plants suggests more continuous ground cover, better insulation, shallower permafrost, and likely boggy, wet conditions. The early Allerød rise of Fabaceae sedaDNA (particularly *Astragalus* and *Oxytropis*, Fig. 4) may also be indicative of a rise in flora with anti-herbivory defenses as these locoweeds are toxic to grazing fauna. Notably, despite otherwise rapid faunal and floral turnover, our single Younger Dryas core sample may suggest that the mammoth-steppe locally persisted through the Bølling-Allerød chronozone in the Klondike.

Mann et al.^28,29^ and Rabanus-Wallace et al.^30^ contend that a shifting moisture gradient from xeric to mesic-hydric, with the paludification of eastern Beringia, best accounts for the loss of dryland-specialists (*Equus, Mammuthus*), whereas mesic and mixed feeding seasonal fauna (*Rangifer, Cervus, Ovibos*) retained suitable habitats and hydric specialists (*Alces, Homo sapiens*) were able to invade new Beringian niches. While warming, an increasing moisture regime, the arrival of cervid browsers, and the rise of woody shrubs may explain much of the terminal signal decay observed for grazing specialists, this does not explain the relative declines of *Mammuthus* and *Bison* sedaDNA prior to the Bølling–Allerød chronozone (assuming that sedaDNA abundance retains a correlation with palaeo-biomass). Guthrie^143^ found that horses had undergone body-size declines after the LGM until their extirpation in Beringia, which likewise suggests longer-term pressures predating shrub expansion. The rise of woody plants in this dataset thus may be partially explained by both the gradual reduction in local megaherbivore ecosystem engineering over millennia and by a warming climate and shifting moisture regimes.

Unknowns in sedaDNA release, preservation, and recovery restrict what we can confidently infer from differences in relative signal abundance, but there are indications of both top-down and bottom-up contributions to the collapse of the mammoth-steppe in central Yukon. While the decline of faunal sedaDNA is likely influenced to some degree by shifting taphonomic processes (such as warming and increasing moisture causing more DNA degradation), substantial declines in megafaunal sedaDNA predates the Bølling–Allerød interstadial (14,690–12,900 cal BP)^68^. This could be seen as partially supporting Zimov and colleagues’^5,23^ keystone megaherbivore decline model that these animals were increasingly unable to maintain suitable steppe habitats due to declining populations. However, there is a substantial time lag in our dataset between a declining megafaunal sedaDNA signal after 20,000 cal BP and the relative rise of woody shrub sedaDNA ca. 13,500 cal BP. By contrast, the rise of mesic-hydric woody shrub DNA during the Allerød oscillation (13,900–12,900 cal BP) and early Holocene are clearly associated with an abrupt decline in megafaunal sedaDNA, strongly supporting the climate-induced shrub and peatland expansion model of Guthrie^16^ and Mann et al.^28–30^. There is potential support in this dataset for both bottom-up and top-down pressures influencing megafaunal extirpations in the Klondike. Further research is needed to determine whether indications of longer-term top-down pressures are real or an artifact of sedaDNA methodology, and to what degree each Beringian megafaunal species was differentially impacted and/or responsive to shifting ecological pressures.

The degree to which humans may have been involved in any of these transformations is hard to gauge from available evidence (see Supplementary Notes 1.2). Early (>14,000 cal BP) human presence in eastern Beringia is controversial but has been suggested based on possible anthropogenic cutmarks at Bluefish Caves^144–147^ and the identification of allegedly human fecal biomarkers and a coinciding rise of fire activity on the Alaskan North Slope^148–151^. However, these records lack unambiguous artifacts, features, or other clear indications of middle Upper Palaeolithic lifeways as seen in eastern Siberia^152–156^. At this time, there is no clear evidence for an ecologically significant human presence in eastern Beringia prior to ca. 14,000 cal BP (Supplementary Figs. 2–3). Thereafter, low fecundity megafauna^72,157^, who had already undergone millennia of oscillating climatological and ecological pressures, may have been vulnerable to novel anthropogenic forces^25,158–162^ that lack archaeological visibility due to the emergence of post-LGM, high mobility lifeways^156,163^. Currently, evidence of anthropogenic contributions to the ecological turnover in eastern Beringia remain functionally absent, being at most but one enigmatic component in a synergistic set of compounding pressures^25^. SedaDNA analyses of Pleistocene permafrost targeting human DNA may prove key to addressing lingering unknowns in the peopling of Beringia.

### Evidence of a cryptic refugium

**Fig. 7:**
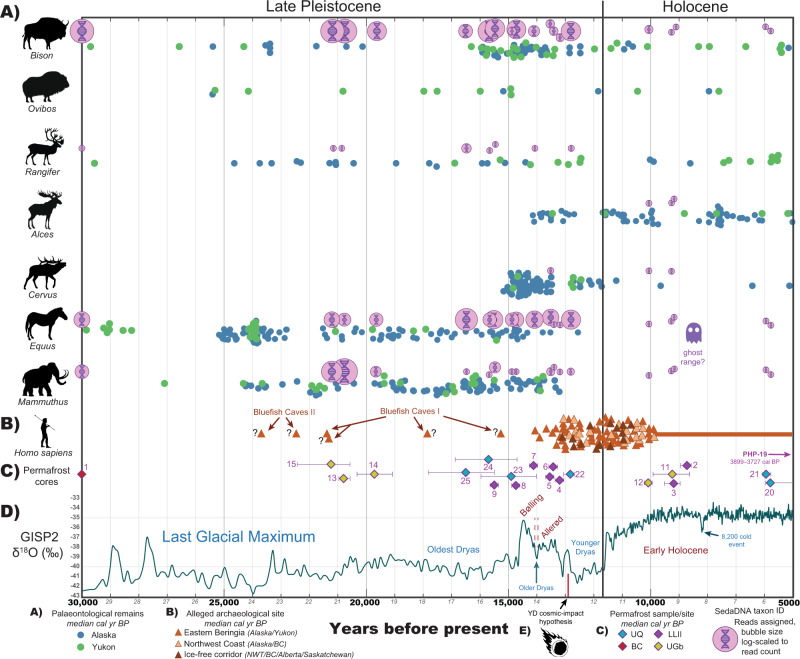
Synthesis of palaeontological, archaeological, climatological, and megafaunal sedaDNA data. Source data are provided as a Source Data file, see the following references for original data sources. **A** Dated fossils of select megafaunal taxa in eastern Beringia^165^, see Supplemental Fig. 1. **B** Earliest archaeological sites in northwestern North America^188^, see Supplemental Figs. 2–3 and Supplemental Table 2. **C** Central Yukon permafrost samples analyzed in this study with 2σ modelled age ranges, see Supplementary Figs. 5–7. **D** Greenland Ice Sheet Project 2 (GISP2) oxygen isotope profiles at 2 m intervals^189^. **E** Onset of the Younger Dryas, and alleged cosmic impact hypothesis^190^.

The persistence of *Equus* and *Mammuthus* until ~9200 cal BP and perhaps as late as ~5700 cal BP (Fig. 2), as suggested by our sedaDNA records, lies well beyond the last dated macrofossils for these taxa (Fig. 7). However, interpreting cryptic populations with sedaDNA necessitates caution. As noted previously, Arnold et al.^91^ found that although permafrost contains a wealth of well-preserved eDNA, the favourable characteristics of perennially frozen ground increases the likelihood for allochthonous organics to survive transport and be redeposited within younger strata. They argue that while reworking is of lesser concern when assessing first appearance dates and “abundant” sedaDNA signals, reworking of older sediments can be an inherent problem when assessing last appearance dates in high-energy fluvial contexts or in areas of thermokarst where older sediments thaw and mobilize followed by potential re-aggradation of permafrost. Arnold and colleagues highlight the careful analysis of loess sediments from the Stevens Village site in central Alaska where Haile et al.^37^ utilized ^14^C, OSL, extensive eDNA sampling on and off site, and careful sedimentological analyses to plausibly infer the late survival of *Mammuthus* and *Equus* to as late as ~10,000 cal BP. While the sediments targeted here are also loessal silts^100^, these materials were not recovered in the field with ancient DNA in mind, but were instead later reselected to follow-up on results presented by Murchie et al.^36^. Although we acknowledge that the signals for late megafaunal persistence should be interpreted with careful skepticism, and require additional supporting evidence for verification (particularly given early Holocene thaw unconformities^97–99^ in the Klondike as identified at Upper Goldbottom and Upper Quartz^100^), these signals are reasonable and worthy of further study for the following reasons.

First, the ghost range signals observed for *Mammuthus* and *Equus* is observed at three different sites in 9 (*Mammuthus*) to 12 (*Equus*) separate permafrost cores and are correlated with substantial and consistent changes in vegetation. In samples younger than ~13,500 cal BP there is a complete restructuring of vegetation. To our knowledge, no plant taxa completely disappeared during the transition, which limits our ability to use the plant data to chronologically test for allochthonous sedaDNA. However, the megafaunal signal observed in the alleged ghost range cores are comparable to those observed during periods of known presence. It is reasonable to ask how many reads are sufficient to say an organism was truly present, but there cannot be any simple answer to that question. At the same time, >50 unique sedaDNA molecules identified as Elephantidae at ~9500 cal BP is significant relative to older cores considering the otherwise substantial ecological turnover.

Certainly in the case of cores with sediments younger than 13,000 cal BP, the presence of *Mammuthus* and *Equus* extending to ~9500 cal BP is highly consistent with other investigations in both northern Asia and northwestern North America^37,41,42,164^. Furthermore, reads for these megafaunal taxa are observed at two sites across all subsampled replicates and are associated with a completely different plant ecological signature. Here the point is that we would expect both floral and faunal sedaDNA would be reworked at roughly similar rates, which is not what is observed. Graminoids and forbs do persist, as would be expected, but there is no obviously mixed ecological signal as woody species dominate the plant metagenomics data (Fig. 4); this is despite otherwise having been observed in modern experiments to be proportionally under-represented genetically compared to their biomass by a factor of ~1:5^95^.

The youngest signatures for *Equus* and *Mammuthus* (ca. 5700 cal BP) are of great interest because they imply local survival of these taxa long after the Pleistocene-Holocene transition. Aside from very late insular occurrences of mammoths from the Bering and Chukchi Seas^41^, there are no accepted radiocarbon dates on mammoth or horse fossils in mainland Beringia that fall anywhere close to the mid-Holocene (Figs. 2, 7, Supplementary Fig. 1). Although the authenticity of the identifications is not in question (Supplementary Fig. 57), the wide temporal gap between these sedaDNA molecules and dated bones is concerning. This difference needs to be evaluated in context. Palaeontological and archaeological records across much of the Arctic and Subarctic are notably sparse. Small refugial populations might have survived in remote pockets at sizes too small to be readily detected by macrofossil collections derived largely from a small set of resource extraction and development sites. The case of *Mammuthus* survival on St. Paul and Wrangel islands, until 5500 and 4000 cal years BP, respectively^41,42^, is interesting because until recent decades neither population was known to have persisted into the mid-Holocene. *Bison priscus* was likewise discovered to have survived throughout the Holocene in southern Yukon^39,40^. Conroy et al.^165^ observed the presence of coprophilous fungi in the Alaskan interior at Windmill and Jan Lakes until ~9000 cal BP and 4500 cal BP. This abundance of spores postdates the megafaunal turnover and is perhaps most likely related to a replacement with browsing cervids. Alternatively, it is also possible that cryptic, refugial populations of Pleistocene grazers were contributing sources. The way forward is through further testing and confirmation, which can be achieved through multiproxy sampling from a broader suite of high latitude sites where reworking is negligible, and conditions favour the widest possible metagenomic spectra.

### Summary

This taxonomically rich sedaDNA dataset tracks the ecological turnover of fauna and flora in central Yukon across the Pleistocene-Holocene transition. We identify the coeval eDNA turnover of megafaunal grazers with forbs and graminoids relative to the rise of woody shrubs and boreal flora at ca. 13,500–13,000 cal BP and after 10,000 cal BP, along with the gradual decline of faunal sedaDNA after 30,000 cal BP. There is also a consistent, multi-site signal of late persistence for *Equus* and *Mammuthus*, perhaps surviving some 7000 years longer than their last dated macrofossils in eastern Beringia would indicate.

Top-down versus bottom-up perspectives on the collapse of the mammoth-steppe cannot be entirely resolved with this dataset, perhaps because both are relevant. The ancient eDNA data presented here have indications of both long term, top-down ecological pressures impacting megafaunal populations in the Klondike, arguably supporting the keystone megaherbivore model of the mammoth-steppe^23,31^ (albeit with unknowns regarding sedaDNA abundance and palaeo-biomass, along with a lag in vegetation turnover). There are also clear, short term, bottom-up indicators signifying climate-induced ecological transformations triggering the megafaunal extirpations between 13,500–10,000 cal BP^16,28–30^. The degree to which a decline of megafaunal DNA prior to the Bølling–Allerød interstadial can be interpreted as indicative of declines in the local populations of grazing megafauna is open for debate. But, as *Equus* sedaDNA remains relatively consistent until the rise of mesic-hydric woody flora during the Allerød warming, this is suggestive of longer-term local declines of woolly mammoths and steppe-bison pre-dating the ecological turnover. The abruptness of the transition thereafter is arguably indicative of both a potential lack of megafaunal engineering controlling woody expansion^4,23,31,121,166^ and the compounding impacts of significant climate change^25^. The local persistence of mammoth-steppe taxa at our single Younger Dryas point sample suggests that the mammoth-steppe locally persisted through the Bølling–Allerød warming, but that subsequent early Holocene transformations significantly shifted the ecological character of the Klondike by 10,000 cal BP.

The late Pleistocene palaeontological record of Beringian megafauna is extensive compared to many other areas of Eurasia and the Americas^27^. However, even with this richness, it is too sparse in well-dated and geographically diverse macrofossils to effectively tease apart the complexity of factors involved in the Pleistocene-Holocene transition and the collapse of the mammoth-steppe. The data presented in this study highlights the power of environmental DNA for the recovery of highly complex signals of ecological change from exceptionally small sediment inputs (0.3–1.35 grams), even in the absence of macro biological tissues. Using targeted enrichment for sedaDNA is also far more cost effective for high-throughput sequencing applications compared to a shotgun approach. Murchie et al.^36^ shotgun sequenced replicates of the same core samples studied here, and in the best sample, a shotgun approach had only 0.007% on-target sequence data (average 0.002%) of ~21 million DNA reads. This contrasts with as much as 10.8% on-target (average 4.2%) here with PalaeoChip enrichments—a 1,538x increase in on-target aDNA. If such a capture enrichment approach were paired with a robust sampling effort focused on sites with clear evidence of stratigraphic integrity across multiple regions and with tight temporal control, these genetic records could prove to be key to unravelling the ambiguities of this extinction event that has confounded Quaternary science since the eighteenth century.

## Methods

Ethical approval was not required for this research as no archaeological or palaeontological materials were processed or analyzed and no human DNA was targeted or analyzed. Cores were acquired with Yukon Science and Explorer License research permits issued to DF by the Yukon Government, Department of Tourism & Culture. A description of the sampling sites, palynology, wet lab master mix recipes, qPCR results, additional bioinformatic analyses and results, and a palaeontological and archaeological background summary are included in the Supplementary Information.

### Summary

Twenty-one core samples of loessal permafrost silts recovered from the Klondike region of Yukon, Canada (Fig. 1, Table 1)—dating between 30,000–4,000 calibrated years before present (cal BP)—were processed for sedimentary ancient DNA (sedaDNA) to evaluate changing biomolecular signals of plants and animals during the late Pleistocene-Holocene transition in eastern Beringia. We used Bayseian age modelling in conjunction with stratigraphic and cryostratigraphic observations to estimate core dates, and a subset of the core samples were processed in parallel for palynology. Despite the exceptional preservation and richness of the sedaDNA, very few pollen grains could be found in the samples (Supplementary Table 4).

For ancient DNA processing, we utilized a sedaDNA modified Dabney et al.^167^ extraction procedure with the long cold spin inhibitor removal technique as described in Murchie et al.^36^, and prepared double stranded, dual-indexed libraries^168,169^ for targeted enrichment. We used the PalaeoChip Arctic-1.0 plant and animal baits^36^ to capture enrich these libraries for chloroplast barcoding loci (*trnL*, *matK*, *rbcL*) of Arctic/Subarctic plants and for whole mitochondrial genomes (or singular loci where mitogenomes were unavailable at the time of bait-design in 2017) of extinct and extant northern animals (focused on megafauna). Libraries were sequenced on an Illumina HiSeq 1500 with 2 × 90 paired-end read chemistry.

After trimming, merging, and filtering the sequenced reads, BLASTn^170^ was used to taxonomically identify the reads to the top 600 hits against a July 2019 local copy of the GenBank database^171^^,172^, which was used as the input for MEGAN Community Edition^173,174^ (v6.19.7, https://github.com/husonlab/megan-ce) and PIA (Phylogenetic Intersection Analysis, v 5.3, https://github.com/Allaby-lab/PIA)^94^. The outputs from MEGAN are plotted in the main text, while plots of individual extraction replicates from both MEGAN and PIA are included in the supplement (Supplementary Figs. 34–55). MapDamage^175^ (v2.0.3, https://ginolhac.github.io/mapDamage/) was used to assess the aDNA damage signals of taxonomically identified taxa (Supplementary Figs. 13–28).

### Field sampling

The cores used in this analysis were previously studied by D’Costa et al.^106^, Mahony^100^, and Sadoway^109^ and have since been kept in cold storage at the McMaster Ancient DNA Centre and the Permafrost Archives Laboratory at the University of Alberta. Permafrost cores were collected between June and August of 2010, 2012, and 2013 with research permits issued to DF from the Yukon Heritage Branch. These cores were sampled at placer gold mining exposures chosen for the quality of the exposure and expected age of the sediments. Prior to sample collection by all three original research teams, the sampling area was cleared of eroded materials back to frozen sediments to create a fresh coring surface for a ~10 cm diameter coring tube ~30 cm in length. Horizontal core samples were drilled with a small portable gas-powered drill (Echo), recovered frozen, stored individually in plastic bags, immediately placed in a −20 °C chest freezer, and transported in the freezer to the University of Alberta or McMaster University for subsampling. Core locations were recorded with a GPS in the field and these locations along with stratigraphic information were recorded in a field notebook at the time of sampling. Horizontal permafrost cores were collected from Bear Creek, Upper Quartz, and Upper Goldbottom. Vertical cores were taken from Lucky Lady II (Fig. 1, Supplementary Methods 2.1).

### Radiocarbon dating and Bayesian age-depth modelling

Plant macrofossils were picked from thawed samples using a dissecting microscope, dried and pre-treated for AMS dating at the University of Alberta along with known-age wood standards (c.f. Mahony^100^). Pre-treatment of all samples followed standard acid-base-acid procedures. Solutions heated to 70 °C and placed in 1 M HCl for 30 min, followed by 60-min washes in 1 M NaOH until the solution became clear. Finally, samples were placed in 1 M HCl for 30 min and rinsed with ultrapure water until they became neutral. Measurements of CO_2_ production, graphitization and radiocarbon abundance in all samples were completed at the Keck-Carbon Cycle AMS facility (UCIAMS).

Age-depth models for Lucky Lady II, Upper Goldbottom and Upper Quartz were first presented by Sadoway^109^ and Mahony^100^. To refine the chronologies of these records we developed new Bayesian age-depth models for each study site using Oxcal v.4.4.2^110^ and the IntCal20 calibration curve^111^. In each case a P_Sequence depositional model was developed along sampling transects or vertical cores using a variable K parameter (increments per unit length)^176^ and a general Outlier_Model, with a 5% prior probability of any ^14^C date being a statistical outlier^177^. Boundaries were placed at the contacts between sediment units where changes in accumulation rates are likely to have taken place.

The Upper Quartz age-depth model (Supplementary Fig. 5) is developed from two P_Sequence depositional models run either side sedimentological boundary at 3.8 m which represents an unconformity of several thousand years. The lower P_Sequence includes two ^14^C dates (UCIAMS-114733 and UCIAMS-114710) and provides chronology for SedaDNA samples; PHP-22, PHP-23, PHP-24, and PHP-25. The upper P_Sequence includes three ^14^C dates (UCIAMS-114899, UCIAMS-114733, and UCIAMS-114710) and provides chronology for SedaDNA samples; PHP-19, PHP-20, and PHP-21.

Lucky Lady II includes three vertical cores which were sampled for SedaDNA (LLII-12, LL2C, and LL2S) (Supplementary Fig. 6). A prominent palaeo-soil that can be traced laterally for hundreds of metres around the exposure is present in the LLII-12 and LL2C cores, and ^14^C dates associated with this horizon were used to combine these cores with a single P_Sequence model (UCIAMS-56390, UCIAMS-114725, UCIAMS-143307, and UCIAMS-143308). Core LLII-12 includes three ^14^C dates (UCIAMS-240139, UCIAMS-122284, and UCIAMS-122273), as well as a palaeo-soil isochron, and provides chronology for SedaDNA samples PHP-9, PHP-8, and PHP-6. Core LL2C includes three ^14^C dates (UCIAMS-142198, UCIAMS-142197, and UCIAMS-143306) as well the palaeo-soil isochron, and provides chronology for SedaDNA samples PHP-4, PHP-5, and PHP-7. A boundary was placed at 3.5 m (at the contact between units 1 and 2) where grey silts including graminoid vegetation are replaced by organic-rich grey and black silts with in situ shrub vegetation. Core LL2-S includes three ^14^C dates (UCIAMS-143296, UCIAMS-142212, and UCIAMS-142211) and provides chronology for SedaDNA samples PHP-3 and PHP-2.

The Upper Goldbottom age-depth model (Supplementary Fig. 7) is developed from two P_Sequence depositional models run either side of a sedimentological boundary at 22.5 m which represents an unconformity of several thousand years. The lower P_Sequence provides chronology for sedaDNA samples PHP-14, PHP-13, and PHP-15, and includes six ^14^C dates (UCIAMS-122282, UCIAMS-114712, UCIAMS-114714, UCIAMS-142208, UCIAMS-114716, and UCIAMS-122274) as well as the Dawson tephra which has been dated to 29,055–29,470 cal BP^178^. The depths of three ^14^C dates obtained from Arctic ground squirrel middens (UCIAMS-122282, UCIAMS-114712 and UCIAMS-114714) were adjusted by 0.8 m to account for burrowing depth. This is likely to be a conservative estimate of active-layer depths during cold stages in the Yukon which were deeper than present. One ^14^C date caused a significant age-reversal and so was excluded from the age-depth model (UCIAMS-240141). Boundaries were placed at 12.6 m and 18 m (at the contacts between units 1, 2, and 3). The upper P_Sequence provides chronology for sedaDNA samples PHP-11 and PHP-12 and includes two ^14^C dates (UCIAMS-114898 and UCIAMS-240143). A further two ^14^C dates were excluded from the model as they caused significant age-reversals (UCIAMS-114910 and UCIAMS-114906).

The single Bear Creek sample (PHP-1) used in this analysis was not re-dated. This core was recovered from immediately beneath the Dawson tephra^179–182^, which is dated to 29,055–29,470 cal BP^178^. As such, this sample can be estimated as being ≳ 30,000 cal BP^106^. See Supplementary Methods 2.1 for a site description.

### Ancient DNA wet lab

Laboratory work was conducted in clean rooms at the McMaster Ancient DNA Centre, which are subdivided into dedicated facilities for sample preparation (separate facilities for subsampling eDNA and discrete materials), stock solution setup, and DNA extraction through library preparation (collectively “pre-amplification rooms”). The post-indexing amplification clean room (enrichment) is in a physically isolated facility from the Centre’s standard aDNA labs, while the subsequent high-copy PCR workspace is in a separate building; the centre has a unidirectional workflow progressing from low-copy to high-copy facilities to reduce the chance of cross-contamination. Each dedicated workspace is physically separated with air pressure gradients between rooms to reduce exogenous airborne contamination. Prior to all phases of laboratory work, dead air hoods and workspaces were cleaned using a 6% solution of sodium hypochlorite (commercial bleach) followed by a wash with Nanopure purified water (Barnstead) and 30 min of UV irradiation at >100 mJ/cm^2^.

### Subsampling

Permafrost sedaDNA subsamples were only taken from core interiors and care was taken to ensure that none of the sampling tools or interior surfaces were exposed any materials that had come in physical contact with the core exteriors (Supplementary Fig. 4). Permafrost samples from Lucky Lady II had been previously subsampled by Sadoway^109^ at the University of Alberta. These subsamples were homogenized by core and transported to McMaster University by Sadoway where they have remained in cold storage. All other subsamples from Upper Quartz and Upper Goldbottom were processed as follows.

Metal sampling tools were cleaned with commercial bleach, rinsed with Nanopure water immediately thereafter, UV irritated on both sides for >30 min, then heated overnight in an oven at ~130 °C. Once the tools had cooled the next day, work surfaces were cleaned with bleach and Nanopure water and covered with sterile lab-grade tin foil. Sediment cores previously split into disks^106,109^ and stored at −20 °C had the upper ~1 mm of external sediment chiselled off to create a fresh sampling area free of exogenous contaminants. For those cores that had not yet been split, a bleach and UV decontaminated handsaw was used to create a groove ~1–2 cm deep around the circumference of the core. A ~1 inch chisel was placed into the groove and a hammer was used to gradually split the core through percussion around the circumference. Once split, this opened a fresh interior surface previously unexposed to sampling equipment. For both the previously split and newly split cores, a small (~1/4 inch) decontaminated chisel was then used to carefully remove interior sediment from the core, which was collected in a weigh boat. After enough material was acquired for multiple extractions (~2–5 g), the core was covered in sterile tin foil and re-frozen. The subsampled material in the weigh boat was homogenized by manually stirring using a small metal chisel as the sediment thawed. This sediment was transferred to a 50 mL falcon tube and refrozen. Thereafter, the work area was thoroughly cleaned with bleach and Nanopure water, all plastic-ware was discarded, and metal tools were placed across the room for decontamination. The now decontaminated workspace was prepared again with sterile tin foil and another core sample. Gloves were changed frequently throughout subsampling (multiple times per core) to minimize cross and exogenous contamination. New metal tools that had been bleach, UV, and heat decontaminated from the previous day were used for each new core and all sterile tools remained isolated in the oven during subsampling. The homogenized sediments for each core were later subsampled for subsequent DNA extractions.

### Lysis and purification

We followed the lysing and sedaDNA extraction procedure described in Murchie et al.^36^. The first round of sediments (PHP) were lysed with an input of 0.3 g. Subsequent experiments determined that this resulted in a higher inhibitor load for certain samples leading to ~10–20% failed or suboptimal adapter ligation efficiencies during library preparations (Supplementary Figs. 8–9). For the second round of extractions (PHP_ii_), we reduced the input to 0.15 g, but used two PowerBead lysing tubes per sample that were pooled on the same Roche column following the long cold spin.

Subsamples were lysed with a digest solution (Supplementary Table 5) preloaded into Dneasy PowerBead tubes, then vortexed for 20 min using a TissueLyser II. Thereafter, the tubes were briefly centrifuged to remove liquid from the lids, and proteinase K was pipetted into each tube individually. The tubes were then briefly finger vortexed to disturb the sediment-bead pellets that had formed at the base and the tubes and were loaded in an incubator to oscillate overnight at 35 °C. The next day, the PowerBead tubes were centrifuged at 10,000 × *g* for 5 min and the supernatant was transferred to a 2 mL MAXYMum Recovery tube and stored at −20 °C for later purifications.

For sedaDNA purification, the digestion supernatant (≈1.25 mL) was thawed, briefly centrifuged, and added to ≳16.25 mL (13 volumes) of high-volume guanidinium binding buffer (Supplementary Table 6) in a 50 mL falcon tube and mixed by repeated inversion. The 50 mL tubes were loaded into a refrigerated centrifuge for the Murchie et al.^36^ long cold spin, where they were centrifuged at 2500 × *g* at 4 °C for ~20 h overnight. Thereafter, the falcon tubes were carefully removed from the centrifuge buckets, and the supernatant was decanted, taking care to not disturb the darkly coloured pellet that had formed during the cold spin. The binding buffer was passed through a high-volume silica-column (High Pure Extender Assembly, Roche Diagnostics) over multiple rounds of centrifugation and extraction proceeded as per Dabney et al.^167^ with binding and wash centrifugation at 3300 × *g*, two rounds of PE wash, followed by two 30 s dry spins at 16,000 × *g* with the tubes rotated 180° between spins to minimize the chance of ethanol retention. Purified DNA was eluted off the silica columns with two volumes of 25 µL EBT (each while waiting 5 min after EBT loading to maximize elution, then centrifuging at 16,000 × *g* for 1 min). Prior to all subsequent experiments the extracts were centrifuged at 16,000 × *g* for ≳5 min to pellet any remaining co-eluted inhibitors. Care was taken when subsampling these extracts to avoid disturbing any pellet precipitates.

### Library preparation, quantitative PCR, and indexing

Doubled stranded libraries were prepared for each extract as described in Meyer and Kircher^168^ with modifications from Kircher et al.^169^ and a modified end-repair reaction to account for the lack of uracil excision (Supplementary Table 7). Samples were purified after blunt-end repair with a QIAquick Nucleotide Removal Kit (QIAGEN) (to maximally retain small fragments) and after adapter ligation (Supplementary Tables 8–9) and indexing (Supplementary Table 10) with a MinElute PCR Purification Kit (QIAGEN). Pre-indexing total library adapted DNA concentrations were estimated as a filtering step to determine whether a sample was successfully convereted into libraries with the short amplification qPCR assay (Supplementary Table 11).

### Targeted capture with PalaeoChip

In-solution enrichments were carried out using the previously designed PalaeoChip Arctic v1.0 bait set^36^. This bait set targets whole mitochondrial genomes from approximately 180 extinct and extant Holarctic fauna, and the chloroplast barcoding loci (trnL, rbcL, and matK) from approximately 2100 species of plants. See Murchie et al.^36^ for further details on the design of PalaeoChip Arctic-1.0.

Enrichments were performed using a modified version of the myBaits v4.1 protocol (Daicel Arbor Biosciences). In summary, hybridization and bait mixes were prepared to the concentrations in Supplementary Table 12. For each library, 7 µL of template was combined with 5 µL of the library block master mix (using xGens, Human Cot-1 DNA, and Salmon Sperm). Hybridization and bait mixes were combined and pre-warmed to 60 °C before being combined with the library-block mixture. The final reaction for batch 1 (PHP) was incubated for 48 h at 55 °C for bait-library hybridization. The second round of libraries (PHP_ii_) were enriched with a hybridization temperature of 60 °C over ~72 h to improve off-target exclusion.

After the hybridization, beads were dispensed (20 µL per reaction [rxn]), washed with 200 µL/rxn of binding buffer, then resuspended in 20 µL/rxn binding buffer and aliquoted into PCR strips. Baits were captured using 20 µL/rxn of the bead binding buffer, incubated at 55 °C for 2.5 min (60 °C for the second round), finger vortexed and spun down, then incubated for another 2.5 min. Beads were pelleted and the supernatant (the non-captured library fraction) was removed and stored at −20 °C as per Klunk et al.^183^. The beads were resuspended in 180 µL of 60 °C Wash Buffer X per tube and washed four times following the Mybaits v4.1 protocol. Beads were resuspended in 18.8 µL EBT, PCR reamplified for 12 cycles (Supplementary Table 10), then purified with MinElute columns following manufacturer’s protocols and eluted in 15 µL EBT.

### Total quantification, pooling, size selection, and sequencing

Libraries were quantified using the long-amplification total library qPCR assay (Supplementary Table 13) and pooled to equimolar concentrations. Pools were size-selected with gel excision following electrophoresis for molecules ranging between 150–500 bp. Gel plugs were purified using the QIAquick Gel Extraction Kit (QIAGEN), according to manufacturer’s protocol, then sequenced on an Illumina HiSeq 1500 with a 2 × 90 bp paired-end protocol at the Farncombe Metagenomics Facility (McMaster University, ON).

### Bioinformatics

Reads were demultiplexed with bcl2fastq (v1.8.4), converted to bam files with fastq2bam (https://github.com/grenaud/BCL2BAM2FASTQ), then trimmed and merged with leeHom^184^ using ancient DNA specific parameters (–ancientdna). Reads were mapped to a concatenation of the PalaeoChip Arctic-1.0 plant and animal probe references with network-aware-BWA^185^ (https://github.com/mpieva/network-aware-bwa) with a maximum edit distance of 0.01 (-n 0.01), allowing for a maximum two gap openings (-o 2), and with seeding effectively disabled (-l 16500). Mapped reads that were merged or unmerged but properly paired were extracted with libbam (https://github.com/grenaud/libbam), collapsed based on unique 5′ and 3′ positions with biohazard (https://bitbucket.org/ustenzel/biohazard) (for PCR deduplication), and converted to fasta files and restricted to a minimum length of 24 bp. Fasta files were additionally filtered to remove any reads with lingering sequence similarity to the Illumina adapter sequences (…/fasta2oneline.pl input.fasta | agrep -v -1 AGATCGGAA | agrep -v -1 TTCCGATCT | tr “\t” “\n” | tail -n + 2 > output.fasta) and were string deduplicated using the NGSXRemoveDuplicates module of NGSeXplore (https://github.com/ktmeaton/NGSeXplore).

These filtered fastas were used as the input for BLASTn^170^, which were aligned against a July 2019 local copy of the GenBank NCBI (National Center for Biotechnology Information;^171^^,172^) nucleotide database set to return the top 600 alignments (unique accession hits) per read with *e*-values less than 1.0E−5 (flags: -num_alignments 600 -max_hsps 1 -evalue 0.00001). The BLASTn outputs were then passed to MEGAN (Community Edition, v.6.19.7)^173,174^ where the BLASTn results were filtered through a lowest common ancestor (LCA) algorithm using the following parameters:

- Min-score = 50 (default)
- Max expected (*e*-value) = 1.0E−5
- Minimum percent identity = 95%
  - Allows 1 base mismatch at 24 bp, 2 at 50 bp, and 3 at 60 bp to account for cytosine deamination and other aDNA characteristic damage or sequencing errors.
- Top percent consideration of hits based on bit-score = 20%.
  - More conservative taxonomic assignments than the 10% default by taking more of the top hits into consideration for LCA assignment.
- Minimum read support = 3
  - Number of unique reads aligning to an NCBI accession sequence for that taxon to be considered for LCA binning.
- Minimum complexity = 0.3
  - Default minimum complexity filter.
- LCA weighted algorithm at 80%.
  - Two rounds of analysis that increases LCA specificity by taking all taxonomic assignments of a library into consideration.

A second set of BLASTn files, identical to those utilized in MEGAN except for being in a different output format (-outfmt “6 std staxids”) were passed to PIA (Phylogenetic Intersection Analysis)^94^ using default inputs. The PIA output was converted to a MEGAN readable format using scripts available on the PIA-accessories GitHub (https://github.com/Allaby-lab/PIA-accessories). All libraries from both the MEGAN-LCA and PIA taxonomic binning approaches were then compiled using MEGAN’s compare feature with absolute read counts. Plant and animal sequences were inspected separately; taxonomic nodes deemed to be ecologically informative (through data exploration prioritizing taxa with high read counts, distinct changes in read abundance through time, or are otherwise known to be ecologically significant organisms) were selected and plotted using MEGAN’s built-in bubble chart feature. In these inter-sample comparative charts, absolute assigned read counts are retained, but the bubble sizes are scaled (either square-root or logarithmic) to visually normalize between samples of differing sequence depths. As these logarithmically scaled bubbles make it difficult to see change through time (whereas linearly scaled bubbles obscure all but the most abundant taxon nodes), a normalized set of MEGAN libraries was used to create stacked proportional charts to more clearly observe changing sedaDNA taxon assignments through time by taking sequencing depth into account. A PCoA was also created through MEGAN with these normalized libraries. All MEGAN charts were exported as EPS files and were visually fine-tuned in Adobe Illustrator^186^.

Thereafter, the closest available genetic references were obtained for notable taxa identified with the MEGAN-LCA and PIA taxonomic binning approaches. All samples were mapped to these references using the same aforementioned procedures but with an additional map-quality filter set to ≥30 with samtools (https://github.com/samtools/samtools), then assessed for ancient DNA typical damage signals using mapDamage^175^ (v 2.0.3).

### Reporting summary

Further information on research design is available in the Nature Research Reporting Summary linked to this article.

## Supplementary information

Supplementary Information

Reporting Summary

## Data Availability

The sedaDNA sequence data generated in this study that can be mapped to the PalaeoChip bait reference sequences have been deposited as bam files in the NCBI SRA database under BioProject: PRJNA722670, Accessions: SRR14265632– SRR14265692. Metagenomic data derived from these mapped reads are provided in the supplementary information and source data files. PalaeoChip reference and bait sequences are available at 10.5281/zenodo.5643845. Source data are provided with this paper.

## Source data

Source Data

## Code availability

All software utilized in this analysis are available online and are referenced and linked to here or in the supplementary information.
