# Supplementary material for: Collapse of the mammoth-steppe in central Yukon as revealed by ancient environmental DNA
Source: Nat Commun. 2021 Dec 8;12:7120. doi: 10.1038/s41467-021-27439-6 (PMC8654998; doi:10.1038/s41467-021-27439-6)
Supplement: Supplementary file 1 — Supplementary Information [file 41467_2021_27439_MOESM1_ESM.pdf]

Supplementary Information

Collapse of the mammoth-steppe in central Yukon as revealed by ancient environmental DNA

List of tables 2
List of figures 3
1. Supplementary Notes 6
1.1 Palaeontology background 6
1.2 Archaeology background 8
2. Supplementary Methods and Results 14
2.1 Site descriptions 15
2.2 Bayesian age-depth modelling figures and tables 18
2.3 Palynology 22
2.4 Ancient DNA wet lab additional methods and results 25
2.4.1 Pre-sequencing qPCR concentration estimates 25
2.4.2 Master mix recipe tables 28
2.5 Bioinformatic supplement 34
2.5.1 Full sample list with core replicates 34
2.5.2 Normalized read proportions by site 36
2.5.3 Assessing ancient DNA damage 39
2.5.4 *mapDamage* plots 42
2.5.5 Depurination and deamination rates 56
2.5.6 Taxonomic binning replicate comparison 63
2.5.7 GC content and ghost range fragment lengths 86
3. Supplementary References 88

List of tables

Supplementary Table 1 Bestiary of Pleistocene-Holocene extinctions based on Stuart[1]. 6

Supplementary Table 2 Potter et al.[14] data of earliest archaeological sites in northwestern North America.. 12

Supplementary Table 3 Permafrost cores and radiocarbon dates used in Bayesian age-depth modelling 21

Supplementary Table 4 Raw palynomorph and non-pollen palynomorph counts 24

Supplementary Table 5 Proteinase K digestion solution 28

Supplementary Table 6 Guanidinium binding buffer 28

Supplementary Table 7 Library preparation: blunt-end repair.. 28

Supplementary Table 8 Library preparation: adapter ligation 29

Supplementary Table 9 Library preparation: adapter fill-in 29

Supplementary Table 10 Indexing PCR 30

Supplementary Table 11 Pre-indexing qPCR short amplification, total quant 31

Supplementary Table 12 Enrichment master-mixes 32

Supplementary Table 13 Post-indexing/enrichment qPCR long amplification, total quant 33

Supplementary Table 14 Sequencing summary and assigned reads of extraction replicates 34

List of figures

Supplementary Figure 1 Radiocarbon dated fossils for eastern Beringian megafauna over the last 30,000 years from Conroy et al. (2020). 7

Supplementary Figure 2 Beringia during the Late Pleistocene. 10

Supplementary Figure 3 Radiocarbon calibration plot of the earliest alleged archaeological sites in northwestern North America. 11

Supplementary Figure 4 Workflow schematic from wet-lab procedures (extraction through sequencing) to *in silico* data analysis. 14

Supplementary Figure 5 Upper Quartz permafrost exposure; age model for horizontal cores collected in 2012. 18

Supplementary Figure 6 Lucky Lady II permafrost exposure. A) Age model for three vertical cores collected at the site. B) Plate from Mahony[34] 19

Supplementary Figure 7 Upper Goldbottom permafrost exposure. A) Age model for horizontal cores collected at the site. B) Plates from Mahony[34] 20

Supplementary Figure 8 Pre-indexing short amplification qPCR assay for estimating total adapted DNA concentrations. 26

Supplementary Figure 9 Post-enrichment long amplification qPCR assay for estimating total adapted DNA concentrations following targeted capture for equimolar pooling. 27

Supplementary Figure 10 Lucky Lady II stacked normalized reads assigned to the rank 'family' in A) Animalia (insects excluded) with read counts, and B) Viridiplantae with proportions. 36

Supplementary Figure 11 Upper Goldbottom stacked normalized reads assigned to the rank 'family' in A) Animalia (insects excluded) with read counts, and B) Viridiplantae with proportions. 37

Supplementary Figure 12 Upper Quartz stacked normalized reads assigned to the rank 'family' in A) Animalia (insects excluded) with read counts, and B) Viridiplantae with proportions. 38

Supplementary Figure 13 On-target mapped reads for *Picea* 40

Supplementary Figure 14 Off-target mapped reads for *Picea* 41

Supplementary Figure 15 *Mammuthus primigenius mapDamage* plots and fragment length distributions. 42

Supplementary Figure 16 *Equus mapDamage* plots and fragment length distributions 43

Supplementary Figure 17 *Bison priscus mapDamage* plots and fragment length distributions. 44
Supplementary Figure 18 *Rangifer tarandus mapDamage* plots and fragment length distributions. 45
Supplementary Figure 19 *Alces alces mapDamage* plots and fragment length distributions. 46
Supplementary Figure 20 *Ovis canadensis mapDamage* plots and fragment length distributions. 47
Supplementary Figure 21 *Lagopus lagopus mapDamage* plots and fragment length distributions. 48
Supplementary Figure 22 *Lepus americanus mapDamage* plots and fragment length distributions. 49
Supplementary Figure 23 *Microtus agrestis mapDamage* plots and fragment length distributions. 50
Supplementary Figure 24 *Uroitellus richardsonii mapDamage* plots and fragment length distributions. 51
Supplementary Figure 25 *Poa palustris mapDamage* plots and fragment length distributions. 52
Supplementary Figure 26 *Artemisia frigida mapDamage* plots and fragment length distributions. 53
Supplementary Figure 27 *Salix interior mapDamage* plots and fragment length distributions............. 54
Supplementary Figure 28 *Picea glauca mapDamage* plots and fragment length distributions............. 55
Supplementary Figure 29 Plotted edit distances for reads mapped to the *Salix interior* chloroplast reference genome 58
Supplementary Figure 30 Fragment misincorporation plots to confirm the presence of hydrolytic deamination for reads mapped to the *Salix interior* chloroplast reference genome 59
Supplementary Figure 31 Deamination rate of *Salix interior* mapped reads against time 60
Supplementary Figure 32 Deamination rate of *Salix interior* mapped reads compared with the temporally nearest Greenland Ice Sheet Project 2 (GISP2) oxygen-18 isotope value 61
Supplementary Figure 33 Depurination rate of *Salix interior* mapped reads against time.................... 62
Supplementary Figure 34 PHP-1 replicates comparison with *PIA* and *MEGAN* taxonomic binning........ 64
Supplementary Figure 35 PHP-2 replicates comparison with *PIA* and *MEGAN* taxonomic binning........ 65
Supplementary Figure 36 PHP-3 replicates comparison with *PIA* and *MEGAN* taxonomic binning....... 66
Supplementary Figure 37 PHP-4 replicates comparison with *PIA* and *MEGAN* taxonomic binning........ 67
Supplementary Figure 38 PHP-5 replicates comparison with *PIA* and *MEGAN* taxonomic binning........ 68
Supplementary Figure 39 PHP-6 replicates comparison with *PIA* and *MEGAN* taxonomic binning........ 69
Supplementary Figure 40 PHP-7 replicates comparison with *PIA* and *MEGAN* taxonomic binning........ 70
Supplementary Figure 41 PHP-8 replicates comparison with *PIA* and *MEGAN* taxonomic binning........ 71

Supplementary Figure 42 PHP-9 replicates comparison with *PIA* and *MEGAN* taxonomic binning........ 72
Supplementary Figure 43 PHP-11 replicates comparison with *PIA* and *MEGAN* taxonomic binning...... 73
Supplementary Figure 44 PHP-12 replicates comparison with *PIA* and *MEGAN* taxonomic binning...... 74
Supplementary Figure 45 PHP-13 replicates comparison with *PIA* and *MEGAN* taxonomic binning...... 75
Supplementary Figure 46 PHP-14 replicates comparison with *PIA* and *MEGAN* taxonomic binning...... 76
Supplementary Figure 47 PHP-15 replicates comparison with *PIA* and *MEGAN* taxonomic binning...... 77
Supplementary Figure 48 PHP-19 replicates comparison with *PIA* and *MEGAN* taxonomic binning...... 78
Supplementary Figure 49 PHP-20 replicates comparison with *PIA* and *MEGAN* taxonomic binning...... 79
Supplementary Figure 50 PHP-21 replicates comparison with *PIA* and *MEGAN* taxonomic binning...... 80
Supplementary Figure 51 PHP-22 replicates comparison with *PIA* and *MEGAN* taxonomic binning...... 81
Supplementary Figure 52 PHP-23 replicates comparison with *PIA* and *MEGAN* taxonomic binning...... 82
Supplementary Figure 53 PHP-24 replicates comparison with *PIA* and *MEGAN* taxonomic binning...... 83
Supplementary Figure 54 PHP-24 replicates comparison with *PIA* and *MEGAN* taxonomic binning...... 84
Supplementary Figure 55 Blanks comparison with *PIA* and *MEGAN* taxonomic binning. 85
Supplementary Figure 56 Assessing GC content of reference sequences and binned reads................. 86
Supplementary Figure 57 Ghost range fragment length comparison (absolute unique read counts).... 87

1. Supplementary Notes

1.1 Palaeontology background

Supplementary Table 1 Bestiary of Pleistocene-Holocene extinctions based on Stuart[1].

Losses from 40,000–15,000 cal BP		
Eurasia	Beringia	North America south of ice sheets
Ursus spelaeus (cave bear)	*Arctodus simus* (short-faced bear)	*Eremotherium rusconii* (E. laurillardi sloth)
Crocuta crocuta (spotted hyaena)	*Homotherium serum* (a sabretooth cat)	*Glyptotherium floridanum* (a glyptodont)
Panthera spelaea (cave lion)		*Holmesina septentrionalis* (‘giant armadillo’)
Megaloceros giganteus (giant deer)		*Paramylodon* (*Glossotherium*) *harlani* (Harlan’s ground sloth)
Palaeoloxodon naumanni (Naumann’s elephant)		*Miracinonyx trumani* (American cheetah)
Homotherium latidens (a sabretooth cat)		*Homotherium serum* (a sabretooth)
		Canis dirus (dire wolf)
**Losses after 15,000 cal BP to mid-Holocene**		
Eurasia	Beringia	North America south of ice sheets
Coelodonta antiquitatis (woolly rhino)	*Mammuthus primigenius* (woolly mammoth)	*Panthera atrox* (American lion)
Panthera spelaea (cave lion)	*Panthera spelaea* (cave lion)	*Arctodus simus* (short-faced bear)
Mammuthus primigenius (woolly mammoth)	*Saiga tatarica* (saiga antelope)	*Megalonyx jeffersonii* (Jefferson’s ground sloth)
Bison priscus (steppe bison)	*Equus caballus* (caballine horse)	*Mammuthus columbi* (Columbian mammoth)
Megaloceros giganteus (giant deer)	*Haringtonhippus francisci* (New World stilt-legged horse)	*Mammuthus primigenius* (woolly mammoth)
	Bootherium bombifrons (Harlan’s musk ox)	*Mammut am*ericanum (mastodon)
	Bison priscus (steppe bison) (DNA evidence suggests persistence until ~400 yrs cal BP)	Equidae (horses)
		Platygonus compressus and *Mylohyus nasutus* (extinct peccaries)
		Palaeolama mirifica (extinct lama)
		Hemiauchenia macrocephala (large-headed lama)
		Cervalces scotti (extinct moose)
		Bootherium bombifrons (Harlan’s musk ox)
		Navahoceros fricki (American mountain deer)
		Stockoceros onusrosagris (a pronghorn)
		Camelops hesternus (western camel)
		Castoroides ohioensis (giant beaver)
		Smilodon fatalis (a sabretooth)
		Nothrotheriops shastensis (Shasta ground sloth)
		Oreamnos harringtoni (extinct mountain goat)
		Bison priscus (steppe bison)

*List is not exhaustive.

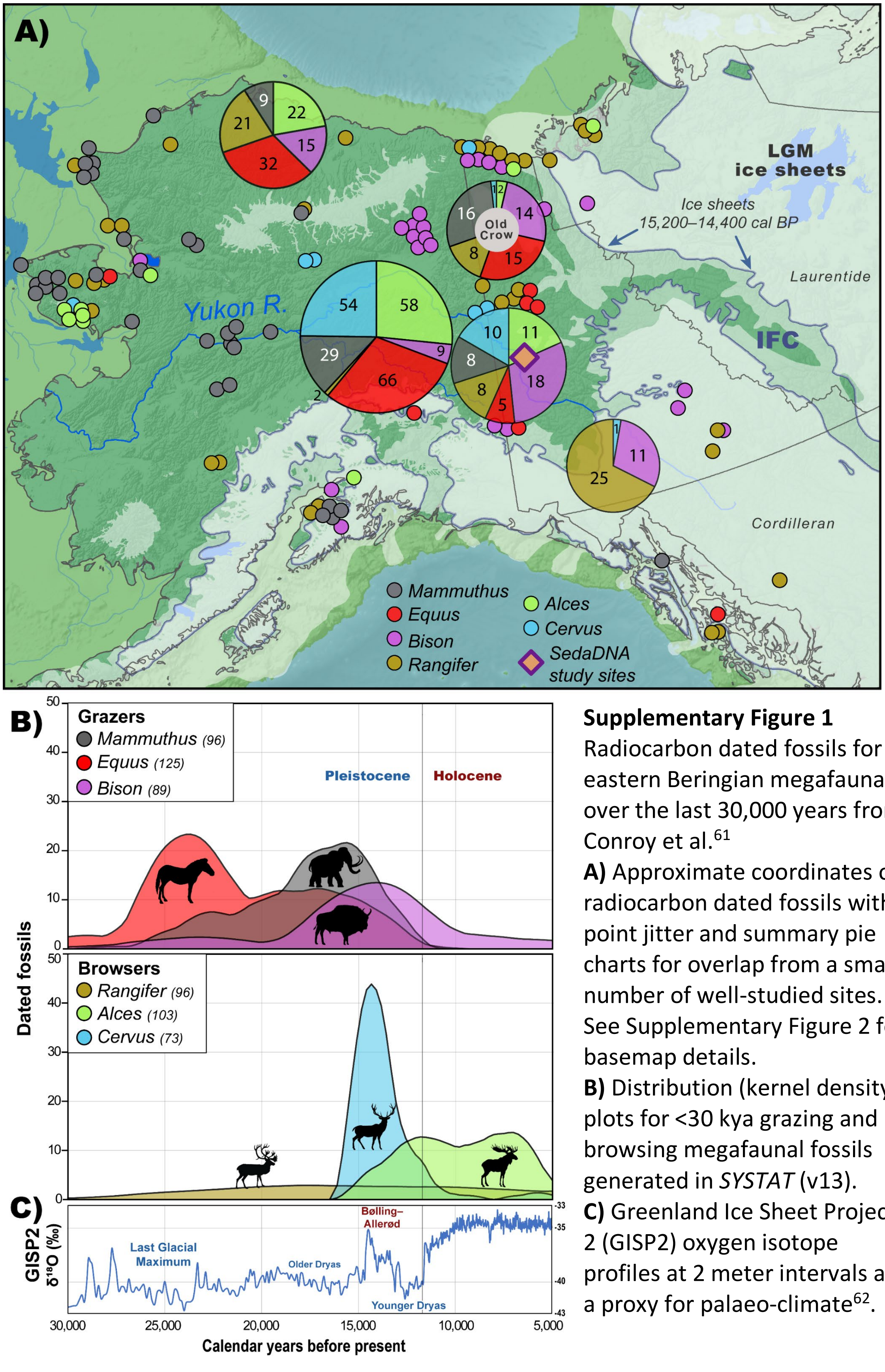

Supplementary Figure 1
Radiocarbon dated fossils for eastern Beringian megafauna over the last 30,000 years from Conroy et al.[61]
A) Approximate coordinates of radiocarbon dated fossils with point jitter and summary pie charts for overlap from a small number of well-studied sites. See Supplementary Figure 2 for basemap details.
B) Distribution (kernel density) plots for <30 kya grazing and browsing megafaunal fossils generated in *SYSTAT* (v13).
C) Greenland Ice Sheet Project 2 (GISP2) oxygen isotope profiles at 2 meter intervals as a proxy for palaeo-climate[62].

1.2 Archaeology background

Assessing when humans became ecologically significant in eastern Beringia is relevant to understanding whether anthropogenic pressures could have contributed to declining megafaunal populations prior to the collapse of the mammoth-steppe ecosystem. The first people to inhabit the northwestern portions of unglaciated North America arrived from Siberia following dispersal across the exposed Bering Isthmus. Humans may have reached the Siberian arctic coast, west of the Yenisey River as early as ~45,000 cal BP[2,3] (Supplementary Figure 2). Evidence during this period is fragmentary, but these first northern peoples had sophisticated Upper Palaeolithic technologies, including prepared lithic blade-cores and abundant bone tools[4,5]. The earliest evidence of humans in western Beringia, east of the Lena River[6], is ~32,000 cal yr BP[7]. This site has an abundance of faunal remains, including lithic and osseous tools, and projectile point fragments embedded in two differently-aged woolly mammoths[7].

Humans appear to have thrived in Siberia until the peak of the Last Glacial Maximum (LGM, 26,500–19,000 BP)[8], although to date it appears as if these populations largely remained in western Beringia[6]. A series of alleged archaeological components at Bluefish Caves I and II in northern Yukon date to as early as ~23,800 years ago[9,10] based on bones that have been debatably interpreted to exhibit human-made cut-marks and other modifications. Sedimentary biomarkers of faecal sterols and polycyclic aromatic hydrocarbons have also been controversially interpreted to be suggestive of a human presence on the Alaskan North Slope from 34,000–16,000 cal BP, along with a rise in fire activity[11]. However, the strongest evidence of sustained human occupations of ecological significance in eastern Beringia comes from sites in the Tanana River Valley, Alaska, chiefly at Swan Point CZ_4[12,13] dating to ~14,000 years ago[14].

Middle Upper Palaeolithic occupations around Lake Baikal and the Yenisey and Lena drainages in Siberia have evidence of semi-subterranean dwellings, storage pits, diverse faunal remains (large-to-small mammals, birds), and a substantial quantity of lithic debris and other artifacts[6,15–18]. These people hunted a variety of megafauna, including: *Mammuthus primigenius* (woolly mammoth), *Coelodonta antiquitatis* (woolly rhinoceros), *Bison priscus* (steppe bison), *Rangifer tarandus* (reindeer), and *Equus caballus* (horse)[6,19]. There may have been an abandonment of much of Siberia and Beringia during the LGM due to the extreme cold climatic conditions[4,17,19–24], although this is contested[25–27]. It

has been hypothesized that people may have taken refuge farther south in the Russian Far East and on the Palaeo-Sakhalin-Hokkaido-Kuril Peninsula in present day northern Japan[4,28,29].

The people who came to reoccupy Beringia after the LGM seem to have had substantially different lifeways from their predecessors. Late Upper Palaeolithic populations in Siberia had developed a mobile settlement system technologically based on a highly economical and portable use of lithic raw material: microblades[30]. Sites after ~21,000 cal BP lack semi-subterranean dwellings and storage pits[6] and have thin occupation layers with single taxon dominant faunal assemblages[4]. This change in lifeways towards high residential mobility may have been adapted to perusing the shifting ranges of mobile herd fauna[17,22].

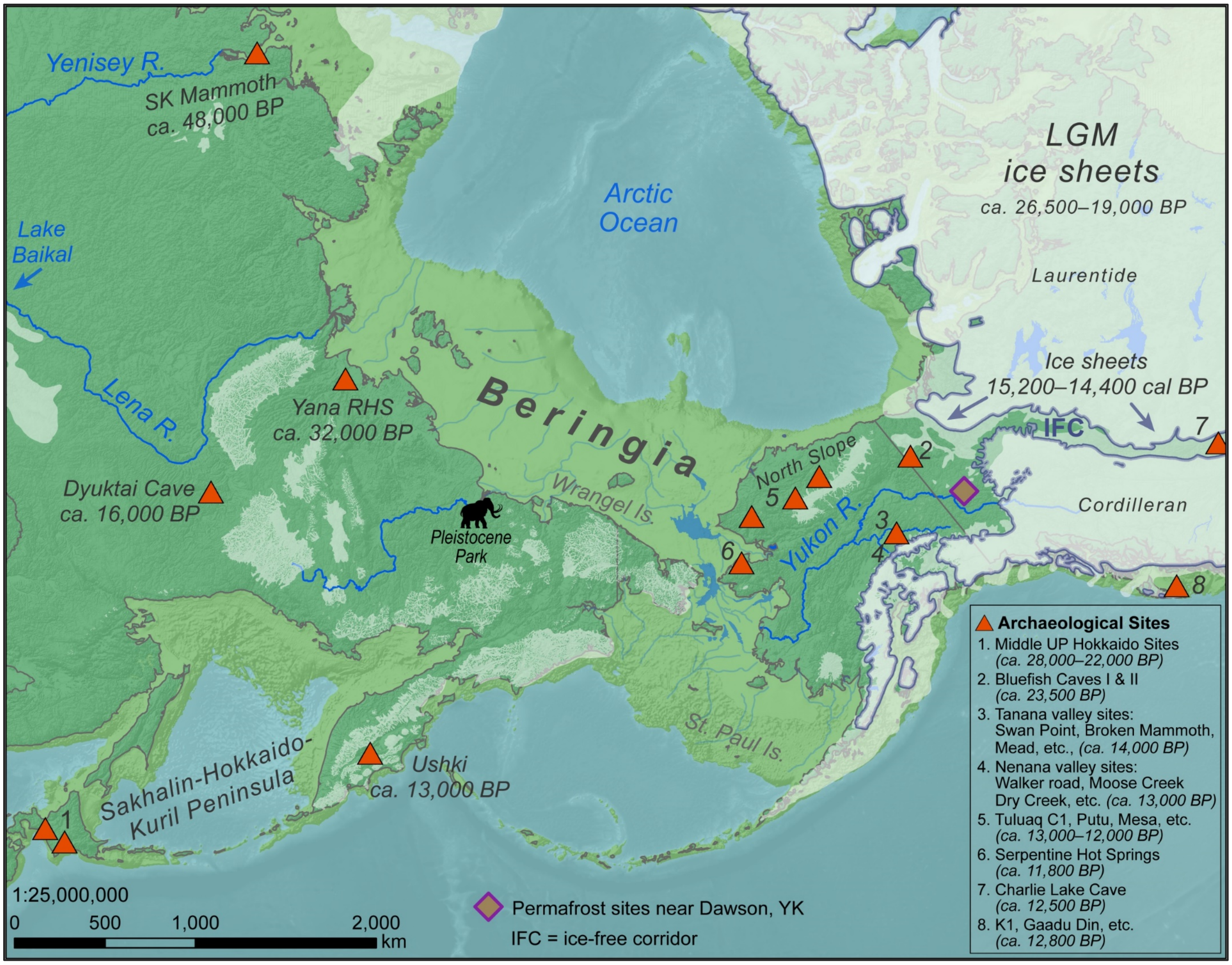

Supplementary Figure 2 Beringia during the Late Pleistocene. Includes select archaeological sites and geographic areas discussed in text. Ice sheet data at Last Glacial Maximum (LGM, 26,500–19,000 BP) (Clark, 2009) and 12,500 ^{14}C BP (15,200–14,400 cal BP) from Dyke (2004). Sea level during LGM set to 126 meters below sea level based on midpoint between maximum and minimum eustatic sea level estimation models in Clark and Mix (2002). Beringian palaeodrainage data from Bond (2019) (http://data.geology.gov.yk.ca/). Archaeological sites with calibrated ages from Potter et al.[14]. Pleistocene Park: https://pleistocenepark.ru/.

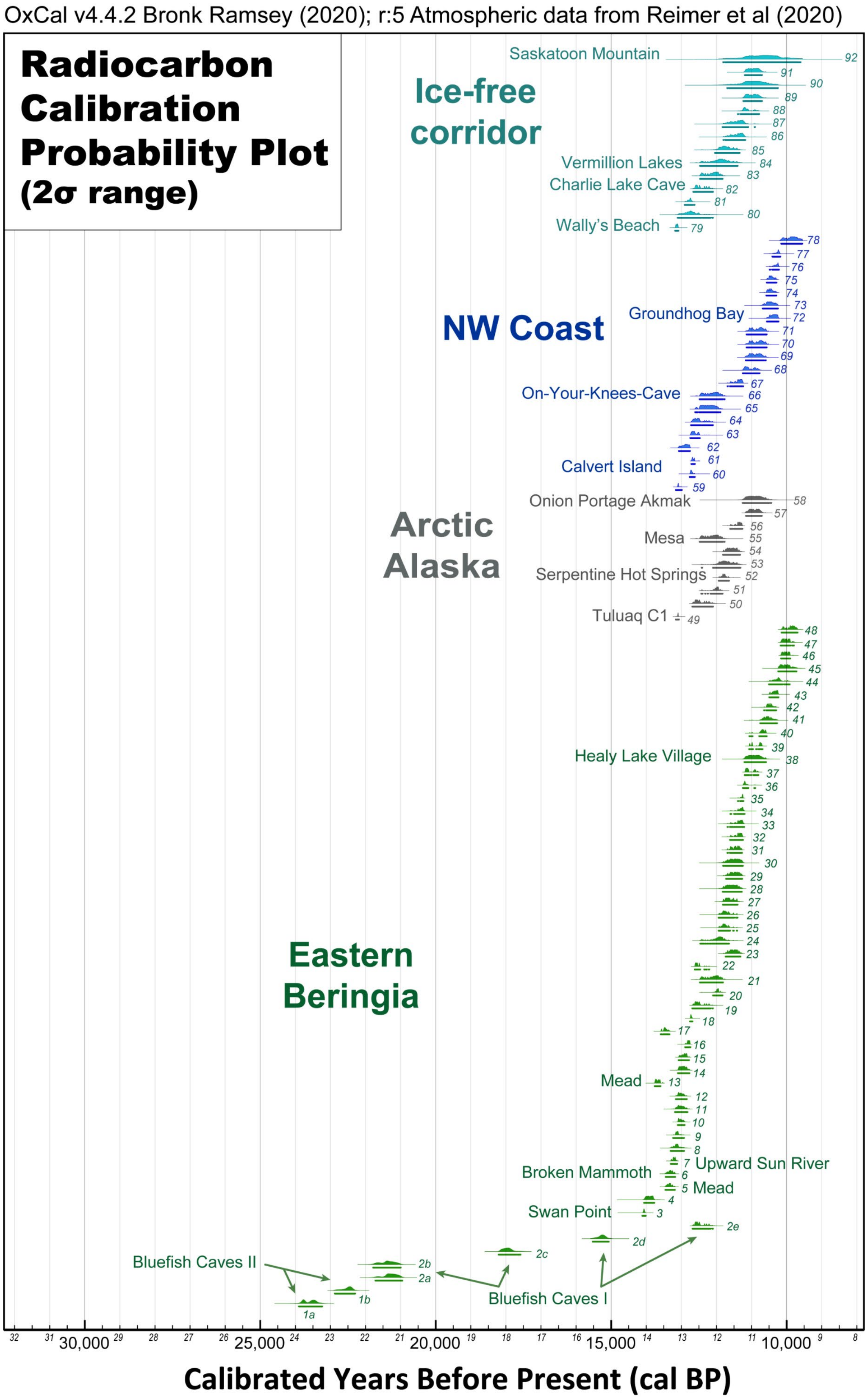

Supplementary Figure 3 Radiocarbon calibration plot of the earliest alleged archaeological sites in northwestern North America. Data compiled by Potter et al.[14]. Calibrated using Oxcal v.4.4.2[31] and the IntCal20 calibration curve[32]. See the supplementary materials of Potter et al.[14] for the original data, and Supplementary Table 2 for the calibrations.

Supplementary Table 2 Potter et al.[14] data of earliest archaeological sites in northwestern North America.

Intcal ID	Site/Component	Radiocarbon age			Calibrated age			Area	Reference in Potter et al. (2018)
		^{14}C yr BP[1]	±	n of dates	From	To	Median cal years BP		
1a	Bluefish Caves II	19,650	130	7	23,900	23,234	23,561	Beringian Interior	49
1b	Bluefish Caves II	18570	110	7	22,873	22,299	22,490	Beringian Interior	49
2a	Bluefish Caves I	17,610	100	7	21,715	20,954	21,268	Beringian Interior	49
2b	Bluefish Caves I	17,660	100	7	21,780	21,000	21,362	Beringian Interior	49
2c	Bluefish Caves I	14,645	75	7	18,205	17,586	17,961	Beringian Interior	49
2d	Bluefish Caves I	12,790	65	7	15,525	15,072	15,262	Beringian Interior	49
2e	Bluefish Caves I	10,490	55	7	12,680	12,103	12,514	Beringian Interior	49
3	Swan Point CZ4	12,160	20	8	14,110	14,015	14,064	Beringian Interior	83
4	Little John sub-paleosol	12,020	70	1	14,061	13,771	13,913	Beringian Interior	84
5	Mead CZ5	11,460	50	1	13,455	13,188	13,337	Beringian Interior	75
6	Broken Mammoth CZ4	11,440	60	2	13,445	13,179	13,313	Beringian Interior	85
7	Upward Sun River C1	11,320	30	3	13,300	13,121	13,211	Beringian Interior	86
8	Walker Road C1	11,220	90	3	13,304	12,925	13,134	Beringian Interior	87
9	Moose Creek C1	11,190	60	1	13,234	12,927	13,114	Beringian Interior	88
10	Linda's Point	11,100	40	2	13,100	12,910	13,021	Beringian Interior	89
11	Dry Creek C1	11,120	90	1	13,180	12,826	13,019	Beringian Interior	90
12	Owl Ridge C1	11,110	60	2	13,157	12,847	13,018	Beringian Interior	91-92
13	Mead CZ4	11,808	20	5	13,760	13,595	13,678	Beringian Interior	75
14	Bachner C1	11,030	70	1	13,095	12,771	12,956	Beringian Interior	93
15	Teklanika West C1	11,000	40	2	13,076	12,785	12,919	Beringian Interior	94
16	Eroadaway	10,890	40	1	12,890	12,744	12,799	Beringian Interior	95
17	MacDonald Creek (FAI-2043)	11,600	50	1	13,585	13,339	13,462	Beringian Interior	96-97
18	MacDonald Creek (FAI-2043)	10,730	40	1	12,753	12,684	12,724	Beringian Interior	96-97
19	Moose Creek C2	10,500	60	1	12,689	12,103	12,524	Beringian Interior	88
20	Mead CZ3	10,270	20	5	12,096	11,830	11,966	Beringian Interior	98
21	Broken Mammoth CZ3	10,290	70	1	12,470	11,818	12,079	Beringian Interior	85
22	Owl Ridge C2	10,490	30	7	12,620	12,206	12,554	Beringian Interior	91-92
23	Owl Ridge C2	10,020	40	6	11,738	11,318	11,516	Beringian Interior	91-92
24	Phipps	10,230	70	1	12,459	11,643	11,928	Beringian Interior	99
25	Upward Sun River C2	10,140	40	2	11,937	11,410	11,769	Beringian Interior	86
26	FAI-2077	10,130	50	1	11,938	11,403	11,742	Beringian Interior	96-97
27	Swan Point CZ3	10,080	40	3	11,816	11,401	11,639	Beringian Interior	83
28	XBD-308	10,050	70	1	11,821	11,280	11,567	Beringian Interior	100
29	XBD-338 C1	>10000	80*	1	11,726	11,269	11,483	Beringian Interior	100
30	XBD-338 C2	10,000	80	1	11,808	11,250	11,500	Beringian Interior	100
31	Upward Sun River C3	9,990	30	3	11,687	11,273	11,454	Beringian Interior	101
32	Whitmore Ridge C1	9,950	40	3	11,612	11,245	11,361	Beringian Interior	102
33	Little Delta River #3	9,920	60	1	11,684	11,212	11,346	Beringian Interior	100
34	Panguingue Creek C1	9,890	50	3	11,603	11,201	11,297	Beringian Interior	103
35	Carlo Creek C1	9,880	20	6	11,390	11,229	11,261	Beringian Interior	104
36	Gerstle River C1	9,740	40	1	11,245	10,897	11,188	Beringian Interior	105
37	Dry Creek C2	9,660	30	9	11,193	10,807	11,101	Beringian Interior	90
38	Healy Lake Village Chindadn	9,580	110	2	11,201	10,589	10,922	Beringian Interior	106
39	Little John Paleosol	9,510	20	6	11,068	10,688	10,771	Beringian Interior	84
40	Gerstle River	9,450	40	2	11,061	10,571	10,681	Beringian Interior	105
41	XBD-303	9,340	80	1	10,751	10,275	10,544	Beringian Interior	100
42	XBD-312	9,290	40	1	10,643	10,297	10,480	Beringian Interior	100
43	Sparks Point	9,170	50	3	10,493	10,235	10,334	Beringian Interior	107
44	Chugwater C2	9,080	90	2	10,502	9,915	10,248	Beringian Interior	108-109
45	XBD-306	8,930	90	1	10,240	9,720	10,024	Beringian Interior	100
46	Gerstle River C3	8880	20	9	10,157	9,900	10,026	Beringian Interior	105
47	Upward Sun River C4	8,870	30	2	10,170	9,793	10,014	Beringian Interior	86
48	Teklanika West C2	8,820	40	1	10,147	9,690	9,863	Beringian Interior	94
49	Tuluaq C1	11,160	20	5	13,157	13,072	13,096	North Alaska	110
50	Putu	10,490	70	1	12,688	12,099	12,487	North Alaska	111
51	Raven Bluff	10,280	30	7	12,432	11,829	11,991	North Alaska	112
52	Serpentine Hot Springs	10,150	30	3	11,935	11,644	11,788	North Alaska	113
53	HAR-006	10,140	90	1	12,421	11,316	11,739	North Alaska	114
54	Irwin Sluiceway	10,050	50	2	11,813	11,330	11,564	North Alaska	115
55	Mesa	10,000	80*		12,478	11,769	12,110	North Alaska	116

Intcal ID	Site/Component	Radiocarbon age			Calibrated age			Area	Reference in Potter et al. (2018)
		^{14}C yr BP[1]	±	n of dates	From	To	Median cal years BP		
56	Nat Pass	9,960	30	2	11,609	11,260	11,364	North Alaska	117
57	NR-5	9,570	60	2	11,158	10,709	10,928	North Alaska	118
58	Onion Portage Akmak	9,570	150	1	11,252	10,440	10,900	North Alaska	119
59	Calvert Island (EjTa-4)	11,140	25	1	13,156	12,991	13,087	NW Coast	39
60	Calvert Island (EjTa-4)	10,720	60	5	12,760	12,620	12,714	NW Coast	39
61	Calvert Island (EjTa-4)	10,625	20	1	12,712	12,618	12,671	NW Coast	39
62	K1 Cave	10,960	80*		13,070	12,755	12,886	NW Coast	120
63	Gaadu Din 1 Cave	10,620	80*	6	12,745	12,475	12,638	NW Coast	121
64	Gaadu Din 2 Cave	10,530	80*	12	12,724	12,102	12,534	NW Coast	122
65	Cardinalis Creek	10,370	80*		12,605	11,890	12,234	NW Coast	123
66	On-Your-Knees Cave	10,300	50	1	12,478	11,769	12,110	NW Coast	124
67	Hunter Island	9,940	50	1	11,683	11,238	11,361	NW Coast	125
68	Namu	9,700	80*		11,247	10,773	11,086	NW Coast	126
69	Kilgii Gwaay	8,540	80*	12	11,165	10,595	10,886	NW Coast	121
70	Hidden Falls	9,500	80*		11,141	10,571	10,811	NW Coast	127-128
71	Arrow Creek 2	9,500	80*		11,141	10,571	10,811	NW Coast	129
72	Ground Hog Bay	9,200	70	2	10,561	10,235	10,370	NW Coast	130
73	Richardson Island	9,290	80*	8	10,680	10,250	10,468	NW Coast	131
74	Irish Creek	9,280	40		10,578	10,295	10,459	NW Coast	132
75	Neck Creek	9,260	30		10,565	10,291	10,437	NW Coast	133
76	Trout Creek	9,130	40		10,482	10,220	10,281	NW Coast	132
77	Rice Creek	9,090	50		10,406	10,181	10,243	NW Coast	132
78	Arrow Creek 1	8,800	80*		10,155	9,556	9,845	NW Coast	129
79	Wally's Beach	11,210	40	4	13,175	13,085	13,128	Ice Free Corridor	53
80	Vermilion Lakes Loc A, C9b	10,770	180	3	13,104	12,103	12,737	Ice Free Corridor	134
81	Lake Minnewanka	10,800	80*		12,905	12,625	12,757	Ice Free Corridor	135
82	Charlie Lake Cave	10,470	50	3	12,659	12,102	12,477	Ice Free Corridor	137
83	Vermilion Lakes Loc A, C9a	10,300	60	4	12,465	11,829	12,092	Ice Free Corridor	134
84	Vermilion Lakes Loc A, C6b	10,200	110	2	12,466	11,399	11,881	Ice Free Corridor	134
85	Twin Pines Layer 2	10,140	80		12,042	11,340	11,742	Ice Free Corridor	138
86	Vermilion Lakes Loc B, C4	9,910	100	3	11,801	11,179	11,388	Ice Free Corridor	134
87	Lindoe (EaOp9)	9,900	120		11,824	10,904	11,391	Ice Free Corridor	139
88	Twin Pines Layer 3	9,750	80		11,388	10,784	11,161	Ice Free Corridor	138
89	Vermilion Lakes Loc A, C6a	9,640	100	2	11,232	10,706	10,965	Ice Free Corridor	135
90	Gap site (DlPo20)	9,600	240		11,690	10,253	10,937	Ice Free Corridor	140
91	J-Crossing (DjPm-16)	9,600	80*		11,190	10,707	10,942	Ice Free Corridor	141
92	Saskatoon Mountain, Level 11	9,380	360	1	11,805	9,605	10,682	Ice Free Corridor	138

[1]Oldest date from site selected.

*^{14}C date with error range not supplied; used median age with ± 80 for calibration using Oxcal v.4.4.2[31] and the IntCal20 calibration curve[32].

2. Supplementary Methods and Results

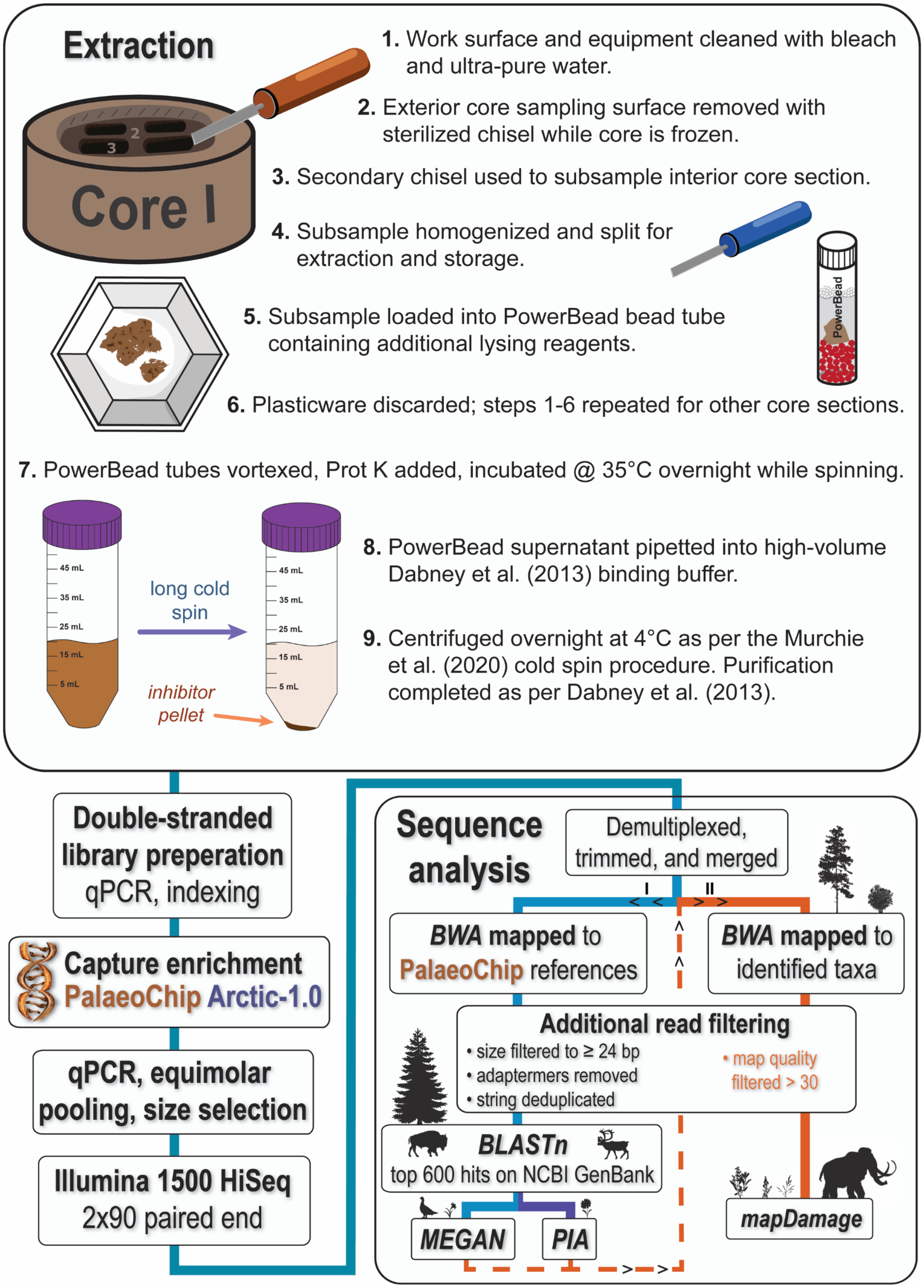

Supplementary Figure 4 Workflow schematic from wet-lab procedures (extraction through sequencing) to *in silico* data analysis.

2.1 Site descriptions

Bear Creek (n=1). The Bear Creek site is located 11 km east of Dawson City, Yukon, in the Klondike placer mining district. Mining activities had exposed a ~10 m vertical surface consisting of 3 m of alluvial sediment overlain by 7 m of ice-rich loessal silt[33]. The Dawson tephra is prominent at the site, dating to 25,300 ^{14}C yr BP (≳30,000 cal BP)[34], and is situated between 5.2 and 6 m from the base of the exposure. Horizontal core sample BC 4-2B was collected 50 cm below the tephra under a stratified lens of ice, likely the remnant of a surface icing similar to other sites associated with Dawson tephra in the area (Froese et al., 2006). The singular core sample used in this analysis from Bear Creek (BC 4-2B) was collected from reddish-brown ice-poor silts that extend below the tephra. These sediments are interpreted as the palaeosurface and include the palaeo-active layer that existed at the time of Dawson tephra deposition. This unit was preserved due to the rapid deposition of the tephra (~80 cm thick at this site) that shifted the active layer upward. Observations of the palaeoactive layer and preservation of the ice body indicate that there was no thawing or water migration in these relict permafrost sediments following deposition of the Dawson tephra[33].

Upper Goldbottom (n=7). This site is located 28 km southeast of Dawson along Goldbottom Creek, a tributary of Hunker Creek and the Klondike River. The 28.5 m exposure was divided by Mahony[35] into five units, dating roughly between 46 ka cal BP near the base, to 6 ka cal BP near the surface. The lowermost unit (0–12.6 m, Unit 1—not represented in this sedaDNA dataset) consists of grey and brown silts with lenses of rust-brown sandy gravel, in addition to *in situ* macrofossils of graminoid vegetation and infrequent wood and shrub remains, as well as *in situ* bones of large mammals, mainly bison and mammoth. This unit is estimated to have been deposited between 43,985 and 35,895 cal BP [35].

Unit 2 (12.6–18.0 m, 32,385–29,400 cal BP) is also not represented in this sample set, but includes brown and grey silts, *in situ* macrofossils in sandy gravel lenses, large ice wedges, and the Dawson tephra (10 cm thick) at 17.7 m[35].

Unit 3 (18.0–22.5 m) consists of grey silts with *in situ* graminoid macrofossils and a few lenses of thin gravels. Four arctic ground squirrel nests were recovered dating between 25,285 to 20,375 cal BP (20,960 ± 150, UCIAMS-131095; 16,895 ± 45, UCIAMS-114712)[35]. The three permafrost samples

from Unit 3 used in this analysis were originally dated to 21,775 cal BP (MM12-117B), 21,000 cal BP (MM12-116B), and 18,510 cal BP (MM12-115B)[35].

Unit 4 (22.5–26.5 m) consist of black and grey organic rich silts with thin (5–15 cm) interbedded lenses of gravels and sand, as well as components of green-grey silts and interbedded humified brown organic silts. *In situ* graminoid and shrub macrofossils were also identified. Unit 4 is estimated to have begun deposition ca. 10,600 cal BP (9,395 ± 25, UCIAMS-114910)[35]. Aggragation rates could not be assessed at the time by Mahony as radiocarbon dated samples were laterally-offset. Core samples MM12-118B and -119B were originally dated to 9,685 and 10,340 cal BP respectively[35].

Upper Quartz (n=7). The Upper Quartz site is located 33 km southeast of Dawson in the narrow valley of Quartz Creek, a tributary to the Indian River. Mahony[35] divided the 7 m vertical section into three units. The lowermost unit (0–2.8 m) consists of grey and brown silts with several thin (1–3 cm) discontinuous black organic lenses in the upper ~1 m with abundant *in situ* macrofossils of graminoid vegetation and rare detrital woody macrofossils. Mahony's calibrated radiocarbon dates from this unit indicated a deposition between 16,560–13,510 cal BP (13,680 ± 390, UCIAMS-114710; 11,680 ± 35, UCIAMS-131100). Two arctic ground squirrel nests were identified at 0 and 0.75 m from the base in this unit, with ^{14}C dates of 16,270 and 15,740 cal BP respectively (13,510 ± 45, UCIAMS-131096; 13,110 ± 35, UCIAMS-142195). Aggradation rates for this unit were estimated by Mahony[35] to range from 0.06–0.14 cm/year. Permafrost cores MM12-QC-2, -3, and -4 were sampled from this unit, and were originally dated to 16,560 cal BP (13,680 ± 390, UCIAMS-114710), 15,745 cal BP, and 14,925 cal BP respectively[35].

The second unit (2.8–3.8 m) consists of grey and black silts, a laterally continuous black organic rich layer with *in situ* macrofossils of shrubby roots and graminoid vegetation. The unit also contains several aggregational ice lenses (2 cm thick), one syngenetic ice wedge, and abundant non-parallel, wavy microlenticular cryostructures[35]. Shrub macrofossils had date ranges between 13,685 and 13,160 cal BP (11,885±35, UCIAMS-142206; 11,315±35, UCIAMS-142205), although aggradation rates could not be estimated. Core MM12-QC-6 was sampled from this unit, and was ^{14}C dated to 12,805 cal BP (10,960 ± 35, UCIAMS-114733).

The uppermost unit (3.7–7.0 m), consists of organic-rich black and grey silts, interbedded discontinuous diamict and sand lenses (10–15 cm thick) with *in situ* macrofossils of fibrous organics and wood. Cryostructures are generally non-parallel wavy lenticular (2–6 mm) with microlenticular ice, crustal ice, and a syngenetic ice wedge (~50 cm wide). Aggradation rates were estimated at 2.0 cm/year[35]. Radiocarbon samples had dates ranging from 5,925 to 5,165 cal BP (5,160 ± 20, UCIAMS-114899; 4,520 ± 25, UCIAMS-142207). Permafrost cores MM12-QC-8, -9, and -10 were sampled from this unit, and were originally dated to 5,915, 5,840, and 5,765 cal BP[35]. The youngest core has been found to be much younger with redating (Supplementary section 2.2), with a new median age of 3849 cal BP.

Lucky Lady II (n=8). The Lucky Lady II site is located 46 km southeast of Dawson along the Sulphur Creek tributary of the Indian River. Core samples from this site were previously analyzed by both Mahony[35] and Sadoway[36], with Sadoway using PCR metabarcoding and targeted enrichment techniques.

An 11.5 m section of the Lady Lady II permafrost exposure was sampled by Mahony[35], which was divided into two units; five vertical cores were taken at the site for high-resolution isotopic and radiocarbon analyses. The lowermost unit (0–3.5 m) unit consists of grey silts with a thick black-organic rich horizon (palaeosol) at 2.7 m from the base dating from 13,410 to 13,140 cal BP (11,580 ± 35 ^{14}C yr BP, UCIAMS-143308; 11,290 ± 160 ^{14}C yr BP, UCIAMS-56390) with several thinner palaeosol horizons above. The unit includes *in situ* graminoid macrofossils and multiple arctic ground squirrel nests; several horse and bison bones were also identified. The unit is suggestive of a steppe-tundra landscape, which is estimated to have been deposited from ca. 16,500 to 13,140 cal BP (13,680 ± 35 ^{14}C yr BP, UCIAMS-51324 to 11,290 ± 160 ^{14}C yr BP, UCIAMS-56390), with an aggradation rate of 0.12–0.62 cm/year[35] and with deep active layers. Samples PHP-4 through PHP-9 were recovered from this unit, dating between 15,865 and 13,105 (11,250 ± 20 ^{14}C BP) cal BP[36].

The uppermost unit (3.5–11.5 m) consists of organic-rich grey and black silts with more organic-rich sediments towards the top of the unit exposure. Several black organic-rich horizons (2–6 cm thick) are interspersed throughout the unit. The defining contact between the upper and lower units at Lucky Lady II is the appearance of shrub vegetation macrofossils in the uppermost unit. This upper unit contains abundant *in situ* shrub and graminoid macrofossils, but no *in situ* animal remains were

identified. The unit is considered to have been deposited between 13,150 and 8,525 cal BP (11,300 ± 35, UCIAMS-142197; 7,750 ± 25, UCIAMS-143296) at an aggradation rate of ~0.12–0.19 cm/year[35]. This unit suggests a shift away from a steppe-tundra ecosystem towards one with an abundance of shrub vegetation. Permafrost samples PHP-2 and -3 were recovered from this unit and dated to 10,220 and 10,340 (9195 ± 20 ^{14}C BP) cal BP respectively[36].

2.2 Bayesian age-depth modelling figures and tables

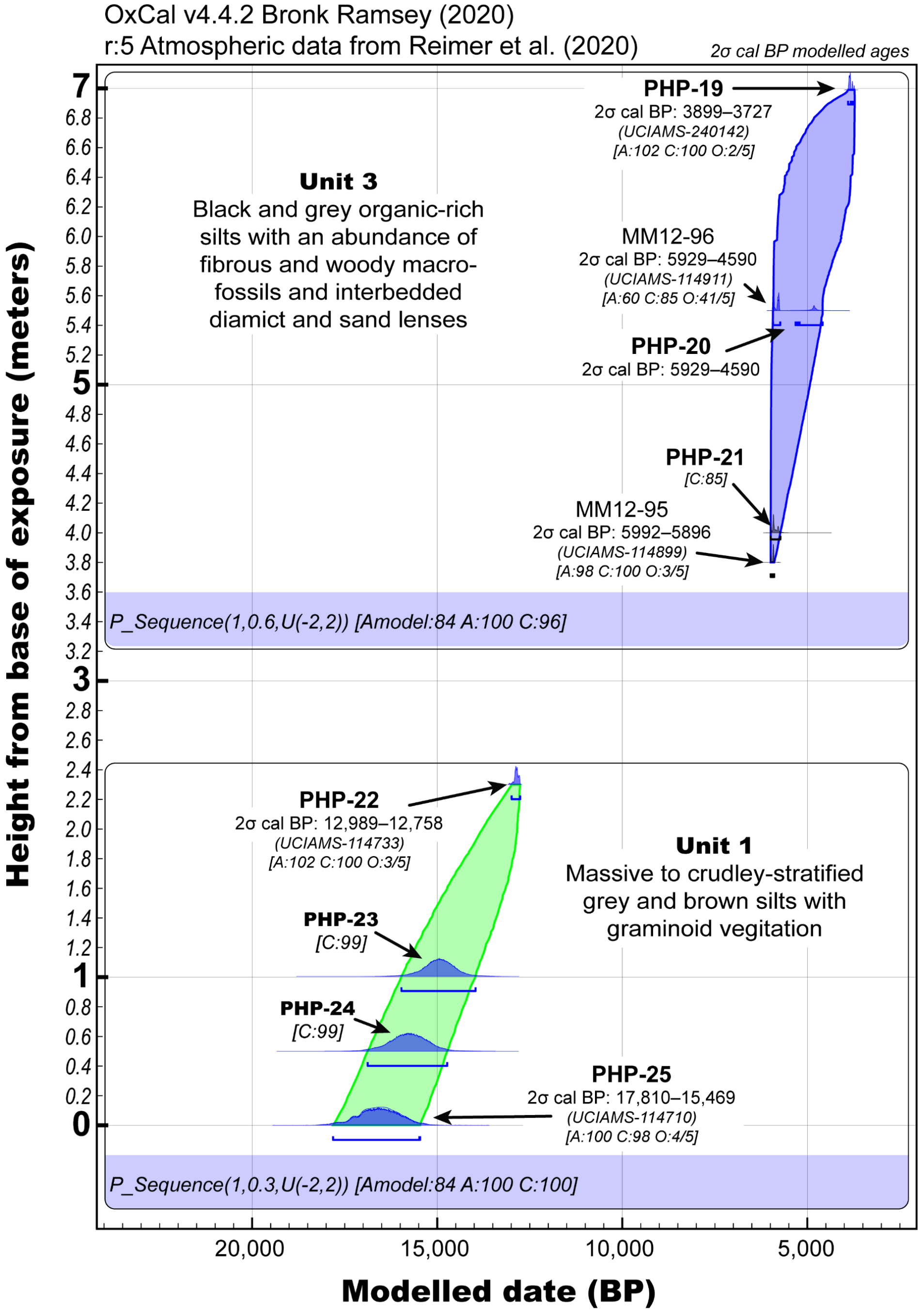

Supplementary Figure 5 Upper Quartz permafrost exposure; age model for horizontal cores collected in 2012.

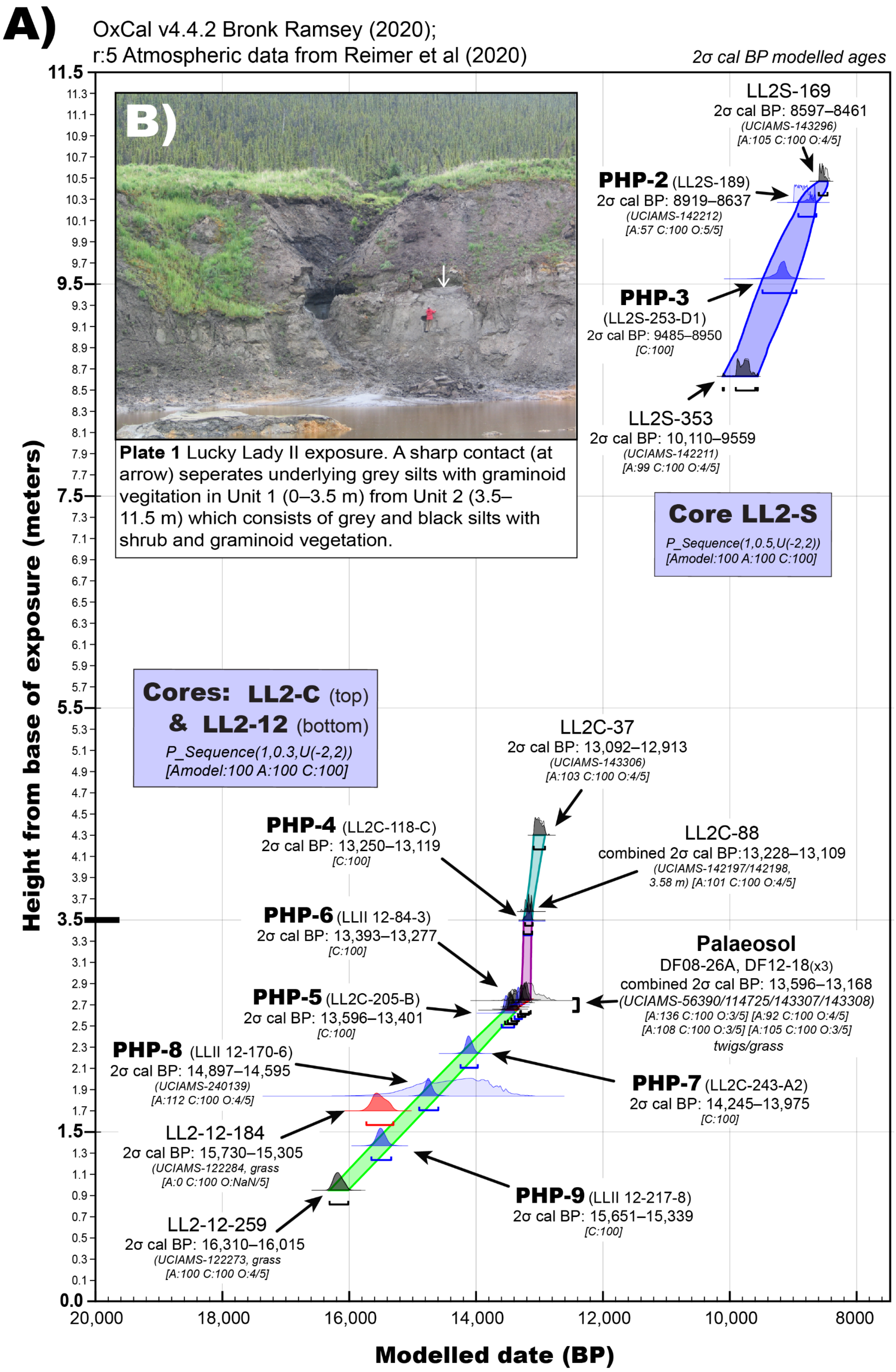

Supplementary Figure 6 Lucky Lady II permafrost exposure. A) Age model for three vertical cores collected at the site. B) Plate from Mahony[35].

A)

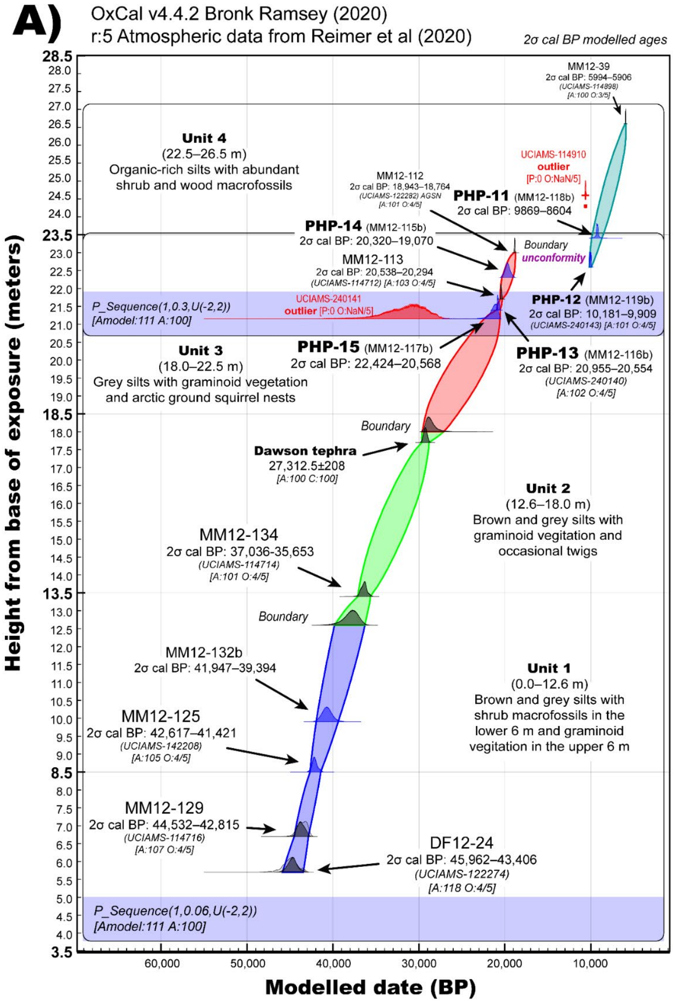

B)

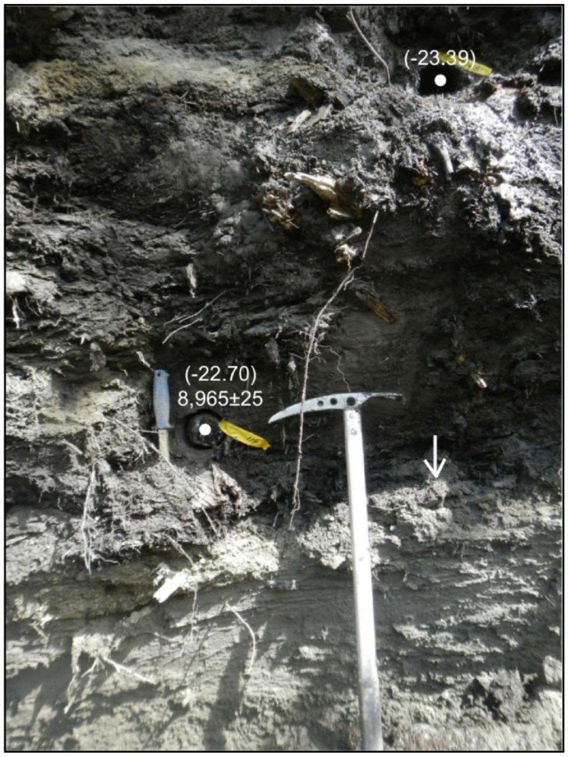

Plate 1 Unit 4 exposure (22.5–26.5 m). Sharp basal contact at arrow with underlying grey silts. Width of ice axe head is 21 cm.

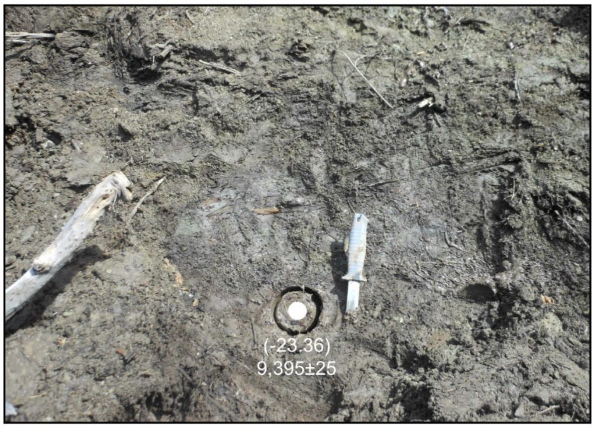

Plate 2 Upper 2 m of unit 4 (24.5–26.5 m). Knife handle is 11 cm long.

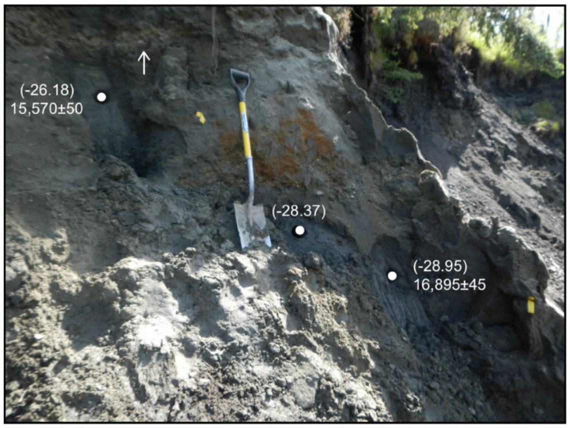

Plate 3 Unit 3 exposure (18–22.5 m). Overlying sandy gravels indicated by arrow. Shovel is ~1.2 m in length.

Supplementary Figure 7 Upper Goldbottom permafrost exposure. A) Age model for horizontal cores collected at the site. B) Plates from Mahony[35].

Supplementary Table 3 Permafrost cores and radiocarbon dates used in Bayesian age-depth modelling

Site & exposure height	Core ID[1]	Height from base (m)	SedaDNA ID	^{14}C Lab ID	^{14}C age		Calibrated age (cal yr BP)			Modelled age (cal yr BP)			Low mass samples (mgC)	Material	Notes
					Median	±	From	To	Median	From	To	Median			
**Upper Goldbottom** 28.5 m	MM12-39	26.6		UCIAMS-114898	5,185	20	5,994	5,907	5,938	5,994	5,906	5,937		wood	
	MM12-124	24.6		UCIAMS-114910	9,395	25	10,700	10,515	10,626	-	-	-		wood	Outlier
	MM12-118b	23.4	PHP-11							9,869	8,604	9,246			
	MM12-121	22.85		UCIAMS-114906	8,965	25	10,227	9,921	10,166	-	-	-		wood	Outlier
	MM12-119B	22.6	PHP-12	UCIAMS-240143	8,905	25	10,178	9,908	10,036	10,181	9,909	10,051		twig	
	MM12-115b	22.3	PHP-14							20,320	19,070	19,704			
	MM12-112	22.2		UCIAMS-122282	15,570	50	18,940	18,770	18,852	18,943	18,764	18,850		AGSN[2], FP[3]	adj 23.0[4]
	MM12-116b	21.4	PHP-13	UCIAMS-240140	17,230	80	20,952	20,556	20,786	20,955	20,554	20,787	0.200	twig	
	MM12-117b	21.15	PHP-15	UCIAMS-240141	26,420	1,920	36,870	27,275	31,228	22,424	20,568	21,232	0.029	twig	^{14}C an outlier
	MM12-113	20.9		UCIAMS-114712	16,895	45	20,530	20,294	20,425	20,538	20,294	20,430		AGSN	adj 21.7
	MM12-134	12.6		UCIAMS-114714	32,000	320	37,058	35,620	36,367	37,036	35,653	36,363		AGSN	adj 13.4
	MM12-132b	9.9								41,947	39,394	40,703			
	MM12-125	8.5		UCIAMS-142208	37,770	660	42,773	41,379	42,159	42,617	41,421	42,123		wood	
	MM12-129	6.7		UCIAMS-114716	40,270	770	44,620	42,580	43,562	44,532	42,815	43,715		wood	
	DF12-24	5.7		UCIAMS-122274	42,070	980	46,771	43,155	44,909	45,962	43,406	44,769		wood	
**Upper Quartz** 7 m	MM12-QC-10	7	PHP-19	UCIAMS-240142	3,550	15	3,896	3,727	3,847	3,899	3,727	3,849		twig	
	MM12-96	5.5		UCIAMS-114911	5,115	25	5,926	5,753	5,814	5,929	4,590	5,776		wood	
	MM12-QC-9	5.5	PHP-20	UCIAMS-114911						5,929	4,590	5,776			
	MM12-QC-8	4	PHP-21							5,992	5,744	5,909			
	MM12-95	3.8		UCIAMS-114899	5,160	20	5,989	5,897	5,921	5,992	5,896	5,923		wood	
	MM12-QC-06	2.3	PHP-22	UCIAMS-114733	10,960	35	12,990	12,758	12,860	12,989	12,758	12,860	0.190	grass	
	MM12-QC-4	1	PHP-23							15,958	13,988	14,939			
	MM12-QC-3	0.5	PHP-24							16,862	14,720	15,759			
	MM12-QC-02	0	PHP-25	UCIAMS-114710	13,680	390	17,785	15,499	16,565	17,810	15,469	16,563	0.021	grass	
**Lucky Lady II** 11.5 m	LL2S-169	10.47		UCIAMS-143296	7,750	25	8,593	8,448	8,524	8,597	8,461	8,545		twigs	
	LL2S-189	10.27	PHP-2	UCIAMS-142212	7,975	25	8,993	8,652	8,862	8,919	8,637	8,707		needle/ leaves	
	LL2S-253-D1	9.55	PHP-3							9,485	8,950	9,177			
	LL2S-353	8.63		UCIAMS-142211	8,770	30	9,903	9,560	9,767	10,110	9,559	9,776		leaves/twigs	
	LL2C-37	4.3		UCIAMS-143306	11,070	30	13,090	12,909	13,006	13,092	12,913	13,015		twigs/grass	
	LL2C-88(2)	3.58		UCIAMS-142198	11,290	40	13,295	13,100	13,176	13,228	13,109	13,160		shrub/twigs	
	LL2C-88	3.58		UCIAMS-142197	11,300	35	13,297	13,111	13,187	13,228	13,109	13,160		shrub/twigs	
	LL2C-118-C	3.49	PHP-4							13,250	13,119	13,176			
	DF08-26A	2.7		UCIAMS-56390	11,290	160	13,486	12,843	13,194	13,307	13,168	13,241	0.052	twigs/grass	palaeosol
	DF12-18	2.7		UCIAMS-114725	11,360	40	13,314	13,166	13,238	13,339	13,225	13,288	0.160	twigs/grass	palaeosol
	DF12-18	2.7		UCIAMS-143307	11,535	35	13,475	13,318	13,401	13,443	13,324	13,386		twigs/grass	palaeosol
	DF12-18	2.7		UCIAMS-143308	11,580	35	13,571	13,345	13,446	13,499	13,353	13,438		twigs/grass	palaeosol
	LLII 12-84-3	2.7	PHP-6							13,393	13,277	13,335			palaeosol
	LL2C-205-B	2.62	PHP-5							13,596	13,401	13,510			
	LL2C-243-A2	2.24	PHP-7							14,245	13,975	14,113			
	LL2-12-170-6	1.84	PHP-8	UCIAMS-240139	12,200	340	15,389	13,449	14,337	14,897	14,595	14,750	0.250	twig	
	LL2-12-184	1.7		UCIAMS-122284	12,980	60	15,727	15,307	15,531	15,730	15,305	15,532	0.190	grass	
	LLII 12-217-8	1.37	PHP-9							15,651	15,339	15,500			
	LL2-12-259	0.95		UCIAMS-122273	13,430	35	16,310	16,030	16,178	16,310	16,015	16,171		grass	

[1]As per Mahony[35]. [2]AGSN: Arctic ground squirrel nest. [3]FP: fecal pellets. [4]AGSN height adjustment.

2.3 Palynology

Samples were processed and analysed for palynomorphs and non-pollen palynomorphs (NPP) at the University of Alberta. Palynological methods followed slightly modified procedures of Faegri et al.[37] with the addition of high-density separation. Each 4 cm^3 sample was spiked with a *Lycopodium clavatum* spore tablets of known quantity (Batch Number: 483216 [Sep. 2004]; 18,582 spores per tablet ± 3,820) before processing to allow calculations of palynomorphs and NPP concentrations. Sediment was processed with 10% hydrochloric acid, 10% sodium hydroxide, high density separation with 1.9 g cm^{-1} density sodium polytungstate (SPT), 50% hydrofluoric acid, acetolysis (10:1 acetic anhydride: concentrated sulphuric acid), dehydrated using 95% ethyl alcohol and tert-butanol and mounted in (2000 cs) silicone oil. Due to the limited presence of pollen grains throughout the samples, a minimum of 50 *L. clavatum* were counted instead of the 300 pollen grains typical of palynological analysis. This allowed calculation of palynomorph and NPP concentrations and to determine if samples are barren or near absent of pollen grains. Identifications of pollen followed McAndrews et al.[38] and were identified at least to family and more typically to genus. NPP identifications followed[39–45]. NPP taxa are also identified by their Hugo de Vries (HdV) designation where possible to enable cross study comparison[46].

Samples were selected to cover the last ca. 30,000 years. Thirteen samples were counted from the Bear Creek, Upper Goldbottom and Upper Quartz sites (Supplementary Table 5). Pollen counts average at 23 grains (range 0–121.5) with 17 palynomorph taxa identified. Of the 15 samples analyzed, only PHP-19 (3849 cal BP) reached a pollen count greater than 100 grains. The remaining samples were near absent of pollen grains with PHP-22 (12,860 cal BP) and PHP-24 (15,759 cal BP) being completely barren of any palynomorphs. Holocene samples PHP-19, PHP-20 (5776 cal BP), and PHP-11 (9246 cal BP) were the only samples to preserve bisaccate pollen grains of spruce (*Picea*) and pine (*Pinus*) with the addition of a singular spruce stomata.

Non-pollen palynomorph were represented primarily by (semi-)coprophilous fungal taxa (n=315), *Sordaria* (HdV-55), an unknown ellipsoidal ascospore assigned as HdV-834 by Langeveld et al. [47] that likely represents the genus *Arnium*, *Sporormiella* (HdV-113), *Arnium* (HdV-261), *Cercophora* (HdV-112), *Delitschia* (no HdV designation), *Chaeotomium* (HdV-7) and *Gelasinospora* (HdV-1). Other fungi include the arbuscular mycorrhizal genus, *Glomus* (HdV-1103) and the saprophytic genus

Byssothecium (HdV-1030). The NPP is not limited to fungi, a singular oocyte of the neorhabodocoela, *Gyratrix*, was preserved in the Holocene sample MM12-QC-10 (PHP-19) and the colonial chlorophytes *Pediastrum* (HdV-760) and *Botryococcus* (HdV-766) occurred in PHP-23 and PHP-1, respectively.

Due the exceptional preservation and abundance of NPP from the samples, it is unclear why palynomorph concentrations are comparatively so low. There is no indication of palynomorph loss during processing given the presence of the NPP taxa and more importantly, the continued presence of *Lycopodium clavatum* tracers. If *L. clavatum* was missing from the palynological residue, we may then consider a loss of palynomorphs during heavy-density separation, but this is not the case. Similarly, we may consider taphonomic processes leading to loss of the palynomorph grains/spores, but the excellent preservation of NPP in the residue suggests this is unlikely. Further study is required to fully understand what factors may be influencing the occurrence of palynomorphs in these permafrost samples.

Supplementary Table 4 Raw palynomorph and non-pollen palynomorph counts

SedaDNA ID		PHP-19	PHP-20	PHP-21	PHP-11	PHP-12	PHP-22	PHP-23	PHP-24	PHP-25	PHP-14	PHP-13	PHP-15	PHP-1
Site		UQ	UQ	UQ	UGB	UGB	UQ	UQ	UQ	UQ	UGB	UGB	UGB	BC
Median age (2σ)		3849	5776	5909	9246	10,051	12,860	14,939	15,759	16,563	19,704	20,787	21,232	30,000
Target	Element													
**Palynomorphs**														
Lycopodium clavatum spike	Spore	50	50	50	50	51	57	71	70	126	60	114	107	68
**Arboreal Pollen**														
Picea	Pollen	44.5	6	13	50									
Picea mariana	Pollen	9			1									
Pinus	Pollen	2.5	1	1										
Salix	Pollen	0			1							2	2	
Betula	Pollen	1	1	1	3	22				1			1	
Alnus	Pollen	4	2											
Poaceae	Pollen	1	1		2	2					3	1	1	1
Cyperaceae	Pollen				1							1		
Artemisia	Pollen													
Asteraceae cf. *Taraxacum*	Pollen				1							2	3	
Polygonaceae	Pollen				1						1		1	
Chenopodium	Pollen			1				2					1	
Shepherdia canadensis	Pollen			1	1						1			
Indet. Pollen	Pollen	6									1	4	2	2
**Stomata**														
Picea Stomate	Stomata	1		3	2	6								
**Aquatic Pollen**														
Isoetes	Pollen	1				1								1
**Spores**														
Sphagnum	Spore	77	2			1								
Dryopteris-type	Spore	1	3											
Trilete	Spore			4									1	
**Non-Pollen Palynomorphs (NPP)**														
**Fungi**														
Sordaria (HdV-55)	Ascospore	1	2	5	6	3	1	5	10	5	1	15	191	
Sordariale (HdV-834)	Ascospore				4								6	
Sporormiella (HdV-113)	Ascospore									4		11	11	
Arnium (HdV-261)	Ascospore			2		1						5	16	
Cercophora (HdV-112)	Ascospore			1	2							1	4	
Delitschia	Ascospore	1			3					1			1	
Gelasinospora (HdV-1)	Ascospore			1	2					1				
Chaetomium (HdV-7)	Ascospore				2								2	
Byssothecium (HdV-1030)	Ascospore			1	5	1								
Glomus (HdV-1103)	Chlamydospore	1			9		4	3		3	1	7	21	38
**Chlorophytes**														
Pediastrum (HdV-760)	Colony							1						
Botryococcus (HdV-766)	Colony													1
**Other**														
Gyratrix	Oocyte	1												

UQ = Upper Quartz, UBG = Upper Goldbottom, BC = Bear Creek

2.4 Ancient DNA wet lab additional methods and results

2.4.1 Pre-sequencing qPCR concentration estimates

In the initial batch of subsamples (PHP; Supplementary Table 15) sedaDNA recovery and isolation was high, although success was somewhat inconsistent. For example, PHP-2, PHP-3, and PHP-12 had less than 1,000,000 total library adapted DNA molecules (based on a quantitative PCR [qPCR] assay prior to indexing) (Supplementary Figure 8). Generally, low pre-indexing estimates are suggestive of poor ligation success during library preparation—likely as a result of co-eluted inhibitory substances during extraction[48]—or poor aDNA preservation or recovery. This pattern was observed again in the post-enrichment qPCR where almost no PalaeoChip Arctic-1.0 on-target DNA could be isolated from those challenging libraries (Supplementary Figure 9), whereas most other libraries retained sufficient concentrations for pooling and sequencing. A subsequent batch of subsamples (PHP_{ii}) with lower sediment inputs (0.15 g per PowerBead tube, duplicated for each core) improved the DNA isolation of those challenging samples, which enabled the recovery of sedaDNA from all targeted permafrost cores. These duplicated lysed extracts were combined by core following the cold spin during binding to the silica spin column in the high-volume Roche tubes.

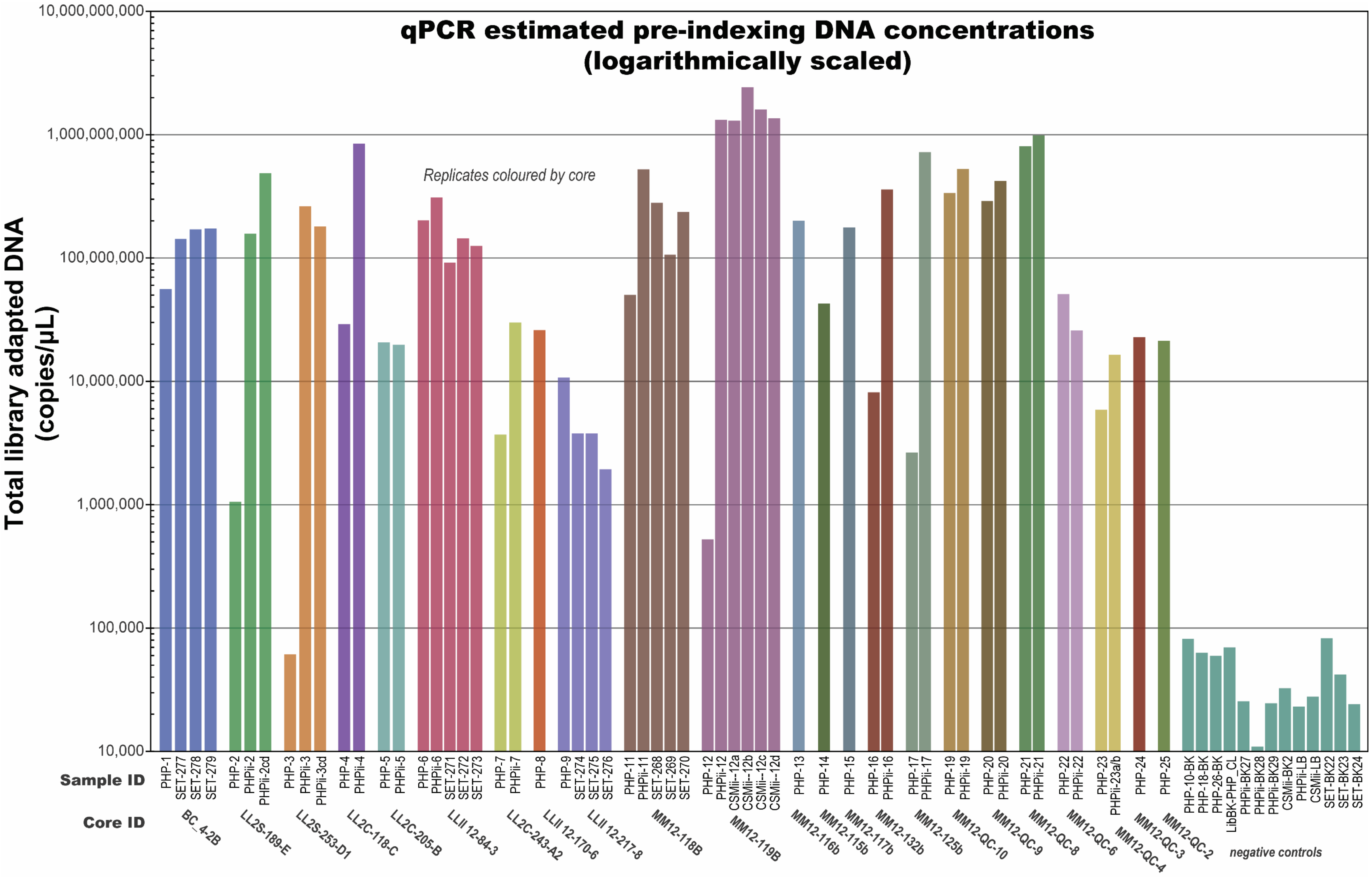

Supplementary Figure 8 Pre-indexing short amplification qPCR assay for estimating total adapted DNA concentrations.

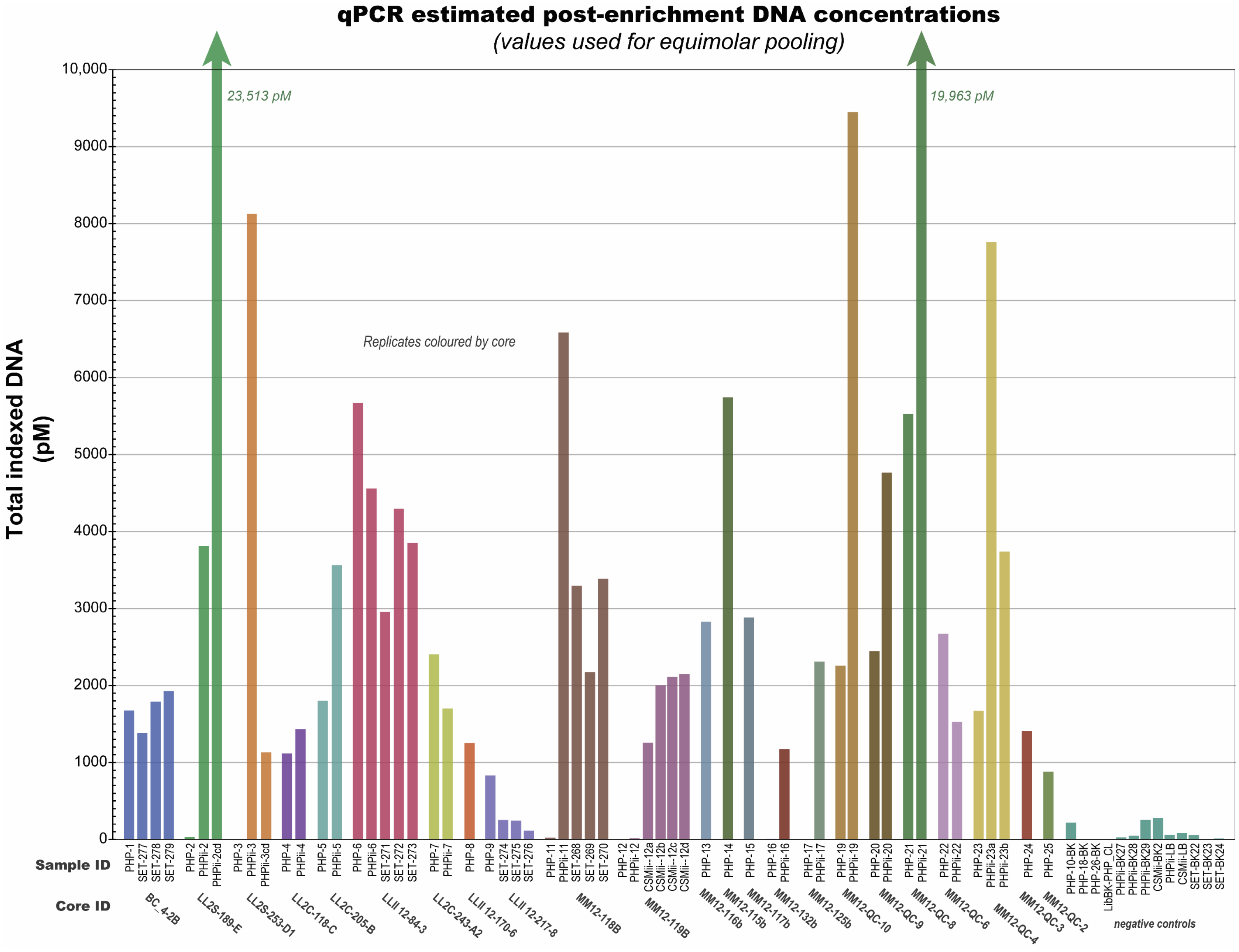

Supplementary Figure 9 Post-enrichment long amplification qPCR assay for estimating total adapted DNA concentrations following targeted capture for equimolar pooling.

2.4.2 Master mix recipe tables

Supplementary Table 5 Proteinase K digestion solution

Proteinase K Digestion Solution	
Component	Final Concentration
Tris-Cl (pH 9.0)	0.02 M
SDS	0.5 %
Proteinase K	0.25 mg/ml
CaCl2	0.01 M
DTT	100 mM
PVP	2.5 %
PTB	5 mM
Volume per rxn	0.5 mL

These are the lysis buffer concentrations after being combined with 0.75 mL of the pre-loaded PowerBead solution (garnet beads and 750 µL 181 mM NaPO4, 121 mM guanidinium isothiocyanate). These concentrations were multiplied by 2.5x prior to loading to account for the pre-loaded PowerBead dilution. Protenaise K added individually (15.53 µL) after vortexing for >20 minutes. Samples digested overnight at 35°C with rotation. Nanopure Barnstead water was used to bring up the volume to the desired concentration. Concentrations based on Karpinski et al.[49].

Supplementary Table 6 Guanidinium binding buffer

Guanidinium Binding Buffer	
Component	Final Concentration
Guanidine Hydrochloride	5 M
Isopropanol (100%)	40 %
Tween-20	0.05 %
3 M Sodium Acetate (pH 5.2)	0.09 M

Nanopure Barnstead water was used to bring up the volume to the desired concentration. Concentrations based on Dabney et al.[50]. Utilized a ratio of 13 volumes of binding buffer to 1 volume of lysis supernatant. With a typical expected volume of 1.25 mL of lysis, 16.25 mL of binding buffer was used.

Supplementary Table 7 Library preparation: blunt-end repair

Blunt-End Repair Mixture	
Component	Final Concentration
NE Buffer 2.1	1X
DTT	1 mM
dNTP mix	100 µM
ATP	1 mM
T4 polynucleotide kinase	0.5 U/µL
T4 DNA polymerase	0.1 U/µL
**Template (DNA extract)**	**10 µL**

A final volume of 40 µL per rxn was used for the master mix (30 µL) and sedaDNA extract. Nanopure Barnstead water was used to bring up the volume to the desired concentration.

Supplementary Table 8 Library preparation: adapter ligation

3. Adapter Ligation Mixture	
Component	Final Concentration
T4 DNA Ligase Buffer	1X
PEG-4000	5%
Adapter Mix	0.5 µM
T4 DNA Ligase	0.125 U/µl
2. Adapter Mix	
IS1_adapter_P5.F	200 µM
IS2_adapter_P7.F	200 µM
IS3_adapter_P5+P7.R	200 µM
Oligo Hybridization Buffer	1X
1. Oligo Hybridization Buffer	
NaCl	500 mM
Tris-Cl, pH 8.0	10 mM
EDTA, pH 8.0	1 mM

Oligo Hybridization Buffer was prepared prior to the Adapter Mix (2), which was prepared separately for IS1 adapter_P5.F and IS2_adapter_P7.F. These two mixes were then combined after an incubation at 95°C for 10 seconds, and a ramp from 95°C to 12°C at a rate of 0.1°C/sec. A final volume of 40 µl was used for the mixture and template DNA. Nanopure Barnstead water (not listed) was used to bring the volume up to the desired concentration.

Supplementary Table 9 Library preparation: adapter fill-in

Adapter Fill-In Mixture	
Component	Final Concentration
ThermoPol Reaction Buffer	1X
dNTP Mix	250 µM
BST Polymerase (large fragment)	0.4 U/µl

A final volume of 40 µl was used for the mixture and template DNA with the addition of Nanopure Barnstead water to bring the mix up to the desired concentration and volume.

Supplementary Table 10 Indexing PCR

Indexing PCR Master Mix	
Component	Final Concentration
KAPA SYBR®FAST qPCR Master Mix (2X)	1X
Forward primer	750 nM
Reverse primer	750 nM
Primer Sequences	
Forward Primer	AATGATACGGCGACCACCGAGATCTACAC***NNNNNNN***ACACTCTTTCCCTACACGACGCTCTT
Reverse Primer	CAAGCAGAAGACGGCATACGAGATTAT***NNNNNNN***ACTGGAGTTCAGACGTGT

Indexing PCR Protocol			
Phase	Temperature (°C)	Time	Cycles
Initial Denaturation	95	5 min	
Denaturation	95	20 sec	Repeated for 8-12 cycles
Annealing + Extension	*60*	*45 sec*	
Final Extension	60	5 min	

The N in each primer sequence represents the 7 bp index specific to each primer. A final reaction volume of 40 µl was used for the assay, with 12.5 µl of the adapter ligated DNA libraries. Nanopure Barnstead water (not listed) was used to bring the volume up to the desired concentration. Fluorescence readings were recorded post-annealing as indicated above with asterisks.

Supplementary Table 11 Pre-indexing qPCR short amplification, total quant

PCR Master Mix	
**Component**	**Final Concentration**
KAPA SYBR®FAST qPCR Master Mix (2X)	1X
Forward primer	0.2 µM
Reverse primer	0.2 µM
**Oligos**	**Sequence (5'–3')**
Forward primer (ILPr_shortampP5F_MeyerIS7)	ACACTCTTTCCCTACACGAC
Reverse primer (ILPr_shortampP7R_MeyerIS8)	GTGACTGGAGTTCAGACGTGT
Library adapted oligo based on the mammoth *12S* mitochondrial gene (Priming sites with reverse-complement bolded)	**ACACTCTTTCCCTACACGAC**GCTCTTCCGATCTCCCTAAACTTTGATAGCTACCTTTACAAAGCTATCCGCCAGAGAACTACAGATCGGAAGAGC**ACACGTCTGAACTCCAGTCAC**
**Input**	**Volume**
PCR master mix	6 µL
Library adapted template	4 µL

PCR Protocol			
**Phase**	**Temperature (°C)**	**Time**	**Cycles**
Initial Denaturation	95	5 min	
Denaturation	95	30 sec	Repeated for 30 cycles
Annealing + Extension	60	45 sec	
Melt Curve	**65–95**	**5 sec per degree**	

Nanopure Barnstead water was used to bring the mix up to the desired concentration and volume. Oligo based on Enk et al.[51]; primers based Meyer and Kircher[52].

Supplementary Table 12 Enrichment master-mixes

Hybridization MasterMix	
**Component**	**Final Concentration**
Hyb N (19.46X SSPE, 13.5 mM EDTA)	9X, 6.25mM
Hyb D (50X Denhardt's Solution)	8.75X
Hyb S (10% SDS)	0.25 %
Hyb R RNAsecure	1.56X
Bait Mixture (200 ng baits per reaction)	11.11 ng/µL
**Bait Mixture**	
**Component**	**Final Concentration**
Plant: 18,672 baits	83.33 ng/rxn
Animal: 57,588 baits	138.89 ng/rxn
**Library MasterMix (blocks)**	
**Component**	**Final Concentration**
Block A (xGens)	0.04 ng/µL
Block C (Human Cot-1 DNA)	0.19 ng/µL
Block O (Salmon Sperm DNA)	0.19 ng/µL
Library template input	7 µL
**Wash Buffer X (0.2X WB)**	
**Component**	**Final Concentration**
HYB S (10% SDS)	0.08 %
Wash Buffer<br>(0.1X SSC; 0.1% SDS; 1mM EDTA)	0.2X

Nanopure Barnstead water was used to bring mixes up to the desired concentration and volume.

Supplementary Table 13 Post-indexing/enrichment qPCR long amplification, total quant

<table>
<tr><th colspan="4">PCR Master Mix</th></tr>
<tr><th colspan="2">Component</th><th colspan="2">Final Concentration</th></tr>
<tr><td colspan="2">KAPA SYBR®FAST qPCR Master Mix (2X)</td><td colspan="2">1X</td></tr>
<tr><td colspan="2">Forward primer</td><td colspan="2">0.2 µM</td></tr>
<tr><td colspan="2">Reverse primer</td><td colspan="2">0.2 µM</td></tr>
<tr><th colspan="2">Oligos</th><th colspan="2">Sequence (5’–3’)</th></tr>
<tr><td colspan="2">Forward primer
(ILPr_shortampP5F_MeyerIS5)</td><td colspan="2">AATGATACGGCGACCACCGA</td></tr>
<tr><td colspan="2">Reverse primer
(ILPr_shortampP7R_MeyerIS6)</td><td colspan="2">CAAGCAGAAGACGGCATACGA</td></tr>
<tr><td colspan="2">PhiX library adapted control standard from 100 pM to 62.6 fM</td><td colspan="2">AATGATACGGCGACCACCGA
ADAPTER INSERT
TCGTATGCCGTCTTCTGCTTG</td></tr>
<tr><th colspan="2">Input</th><th colspan="2">Volume</th></tr>
<tr><td colspan="2">PCR master mix</td><td colspan="2">6 µL</td></tr>
<tr><td colspan="2">Library adapted and indexed template</td><td colspan="2">4 µL</td></tr>
<tr><th colspan="4">PCR Protocol</th></tr>
<tr><th>Phase</th><th>Temperature (°C)</th><th>Time</th><th>Cycles</th></tr>
<tr><td>Initial Denaturation</td><td>95</td><td>5 min</td><td>1</td></tr>
<tr><td>Denaturation</td><td>95</td><td>30 sec</td><td rowspan="2">Repeated for 35 cycles</td></tr>
<tr><td>Annealing + Extension</td><td>60</td><td>45 sec</td></tr>
<tr><td>Cooldown</td><td>8</td><td>30 sec</td><td>1</td></tr>
</table>

Nanopure Barnstead water was used to bring the mix up to the desired concentration and volume. Primers from Meyer and Kircher[52].

2.5 Bioinformatic supplement

2.5.1 Full sample list with core replicates

Supplementary Table 14 Sequencing summary and assigned reads of extraction replicates

Site	Core ID	SedaDNA ID	Age[1]	Input (grams)	Total raw reads	PalaeoChip mapped & MEGAN assigned
Bear Creek[33]	BC 4-2B	PHP-1	30,000	0.25	1,619,859	58,020 (3.58%)
		SET-277		0.25	1,235,759	76,001 (6.15%)
		SET-278		0.25	1,275,219	47,127 (3.70%)
		SET-279		0.25	1,519,972	59,995 (3.95%)
Lucky Lady II[36]	LL2S-189-E	PHP-2	8707	0.3	63,685	250 (0.39%)
		PHPii-2		0.3	4,141,976	8716 (0.21%)
		PHPii-2cd		0.3	4,461,835	942 (0.02%)
	LL2S-253-D1	PHP-3	9177	0.3	not sequenced	
		PHPii-3		0.3	5,445,860	111,232 (2.04%)
		PHPii-3cd		0.3	3,857,668	380,255 (9.86%)
	LL2C-118-C	PHP-4	13,176	0.3	1,619,858	83,631 (5.16%)
		PHPii-4		0.3	6,129,319	95,342 (1.56%)
	LL2C-205-B	PHP-5	13,335	0.3	1,126,060	25222 (2.24%)
		PHPii-5		0.3	3,871,764	23004 (0.60%)
	LLII 12-84-3	PHP-6	13,510	0.3	1,571,297	127,515 (8.12%)
		PHPii-6		0.3	4,287,316	246,751 (5.76%)
		SET-271		0.25	1,807,078	150,779 (8.34%)
		SET-272		0.25	1,322,559	137,744 (10.42%)
		SET-273		0.25	1,247,471	143,812 (11.53%)
	LL2C-243-A2	PHP-7	14,113	0.3	1,449,830	6395 (0.44%)
		PHPii-7		0.3	4,370,642	48,305 (1.11%)
	LLII 12-170-6	PHP-8	14,750	0.3	1,331,181	17,628 (1.33%)
	LLII 12-217-8	PHP-9	15,500	0.3	958,126	15,200 (1.59%)
		SET-274		0.25	845,348	10,397 (1.23%)
		SET-275		0.25	970,115	8778 (0.91%)
		SET-276		0.25	341,707	4191 (1.28%)
Upper Goldbottom[35]	MM12-118B	PHP-11	9236	0.3	79,090	6035 (7.63%)
		PHPii-11		0.3	3,221,976	60152 (1.87%)
		SET-268		0.25	1,678,166	182,089 (10.85%)
		SET-269		0.25	1,754,121	148,670 (8.48%)
		SET-270		0.25	1,249,732	144,575 (11.57%)
	MM12-119B	PHP-12	10,626	0.3	not sequenced	
		PHPii-12		0.3	61,637	296 (0.48%)
		CSMii-PHP-12a		0.3	5,234,065	587,919 (11.23%)
		CSMii-PHP-12b		0.3	3,464,073	441,102 (12.73%)
		CSMii-PHP-12c		0.3	4,649,222	366,785 (7.89%)
		CSMii-PHP-12d		0.3	5,000,444	427,516 (8.55%)
	MM12-116b	PHP-13	19,879	0.3	1,624,580	174,890 (10.77%)
	MM12-115b	PHP-14	18,768	0.3	2,320,965	35,539 (1.53%)
	MM12-117b	PHP-15	20,212	0.3	1,910,387	164,997 (8.64%)
Upper Quartz[35]	MM12-QC-10	PHP-19	3849	0.3	2,279,133	168,768 (7.41%)
		PHPii-19		0.3	6,965,795	21,787 (0.31%)
	MM12-QC-9	PHP-20	5776	0.3	1,883,409	186,238 (9.89%)
		PHPii-20		0.3	4,030,038	126,359 (3.14%)
	MM12-QC-8	PHP-21	5909	0.3	1,590,777	229,319 (14.42%)

Site	Core ID	SedaDNA ID	Age[1]	Input (grams)	Total raw reads	PalaeoChip mapped & MEGAN assigned
		PHPii-21		0.3	3,551,677	36,570 (1.03%)
	MM12-QC-6	PHP-22	12,860	0.3	1,135,493	41,668 (3.67%)
		PHPii-22		0.3	4,944,689	73,310 (1.48%)
	MM12-QC-4	PHP-23	14,939	0.3	1,207,782	26,776 (2.22%)
		PHPii-23a		0.3	5,380,376	17,206 (0.32%)
		PHPii-23b		0.3	8,141,488	24,204 (0.30%)
	MM12-QC-3	PHP-24	15,759	0.3	1,129,623	17,162 (1.52%)
	MM12-QC-2	PHP-25	16,563	0.3	1,568,411	29,419 (1.87%)
Blanks	Extraction Blanks (Batch 1)	PHP-10-BK	na		9,882	81 (0.82%)
		PHP-18-BK	na		1,495	
		PHP-26-BK	na		7,351	
	Library Blanks (Batch 1)	LibBK-PHP_CL	na		4,451	
	Extraction Blanks (Batch 2)	PHPii-BK27	na		2,316	
		PHPii-BK28	na		4,045	
		PHPii-BK29	na		2,170	
		CSMii-BK2	na		8,681	
	Library Blanks (Batch 2)	PHPii-LB	na		4,570	
		CSMii-LB	na		275	
	Extraction Blanks (Batch 3)	SET-BK22	na		55,877	
		SET-BK23	na		852	
	Library Blanks (Batch 3)	SET-BK24	na		475	

[1]Median modeled ages.
Cells shaded by core ID.

2.5.2 Normalized read proportions by site

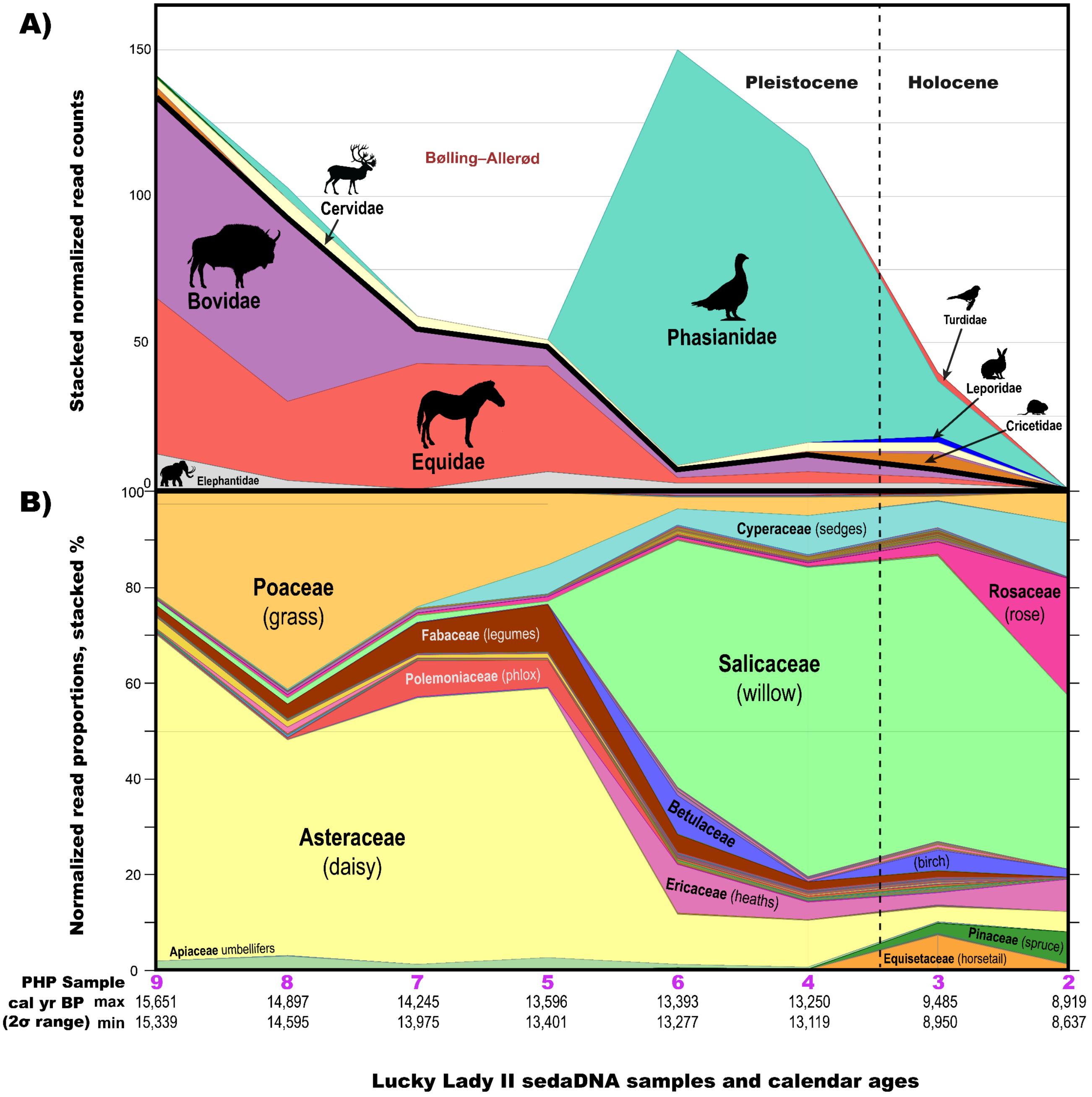

Supplementary Figure 10 Lucky Lady II stacked normalized reads assigned to the rank 'family' in A) Animalia (insects excluded) with read counts, and B) Viridiplantae with proportions.

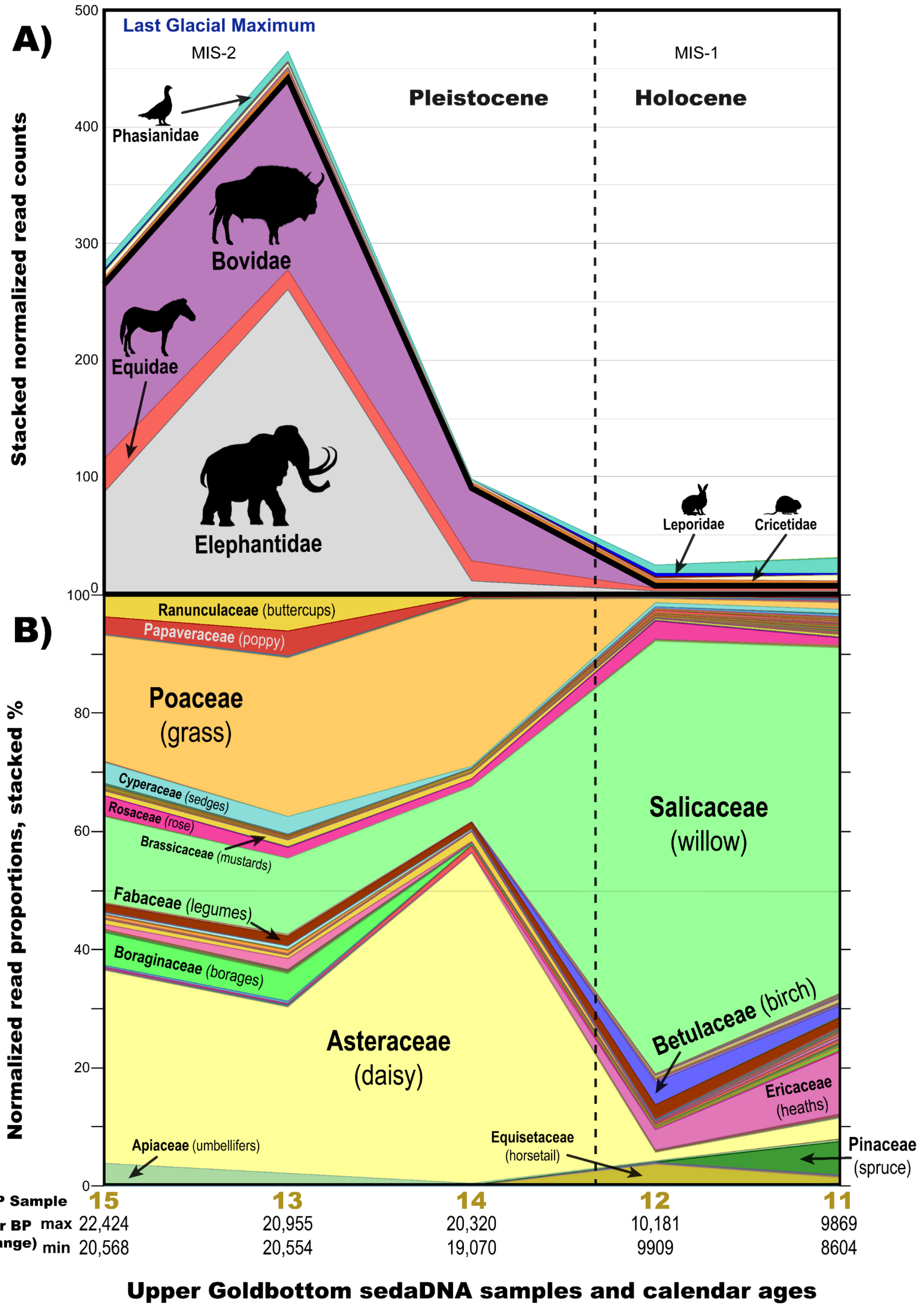

Supplementary Figure 11 Upper Goldbottom stacked normalized reads assigned to the rank 'family' in A) Animalia (insects excluded) with read counts, and B) Viridiplantae with proportions.

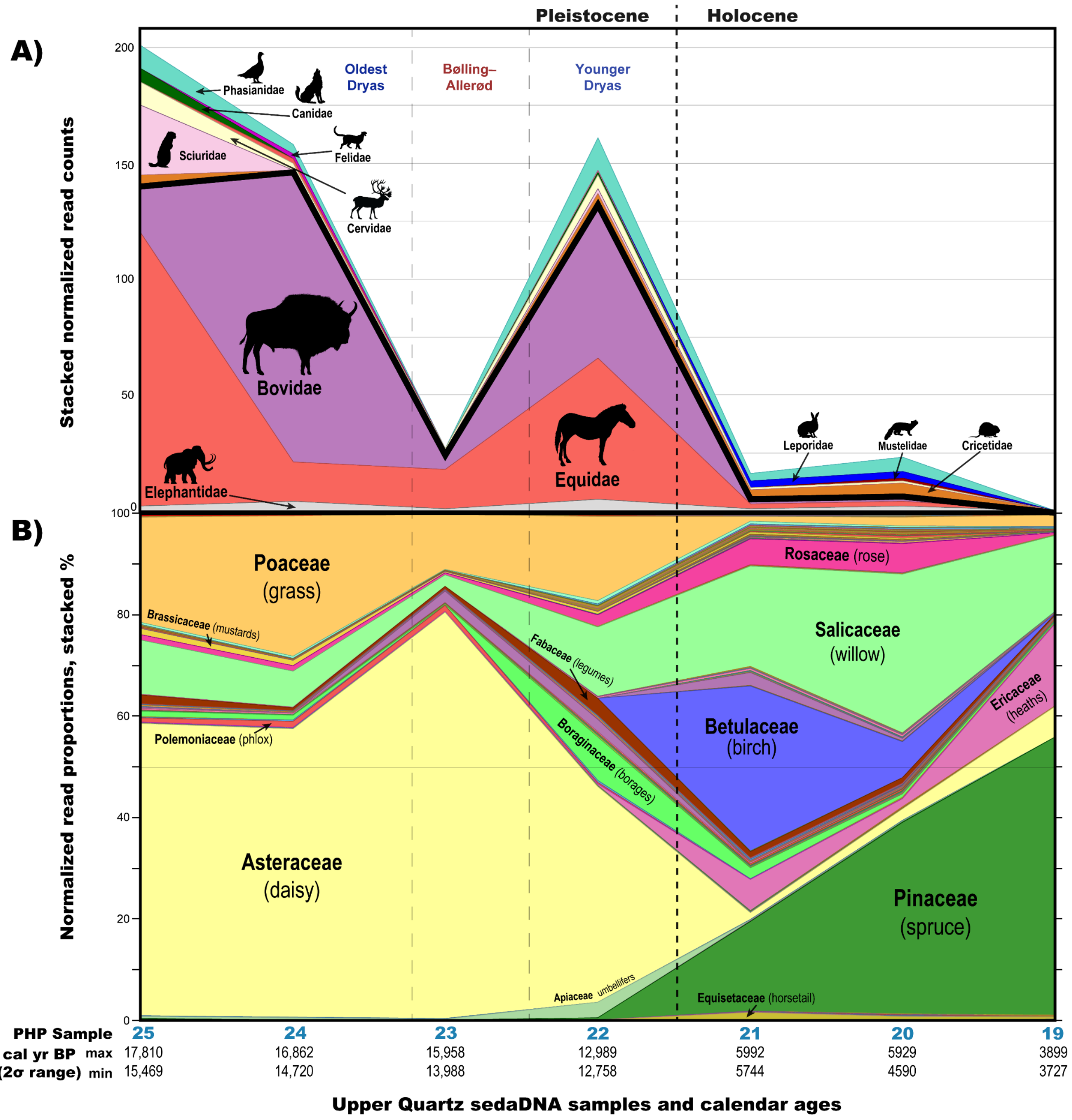

Supplementary Figure 12 Upper Quartz stacked normalized reads assigned to the rank 'family' in A) Animalia (insects excluded) with read counts, and B) Viridiplantae with proportions.

2.5.3 Assessing ancient DNA damage

When mapping to a specific reference, *mapDamage*[53] quantifies the proportion of polymorphic nucleotides in a read relative to the reference to assess whether reads are characteristically damaged on their termini, and as such, are more likely to be ancient. The damage signal being quantified is deamination, which results in C-to-T (or G-to-A) substitutions. These substitution errors rarely occur with modern contaminants. This trend is observed consistently where sufficient read depth is achieved, supporting the argument that these reads are ancient and originate from the samples themselves.

Mapped read counts are inflated however when mapping to a specific organism as compared to taxonomically binned read counts that can be confidently *MEGAN* or *PIA* assigned. This is due to a degree of non-specific mapping of conserved regions of the mitochondrial and chloroplast genomes in highly complex metagenomic samples. This, in some instances, results in stacks of non-specific mapped reads at these conserved sites that contain internal polymorphisms, which causes jagged misincorporation plots and fragment length distributions. Conversely, reads that can be *BLASTn* and *MEGAN/PIA* assigned are individually diagnostic of the identified organism, and hence have smaller counts. Regardless of whether all of the mapped reads utilized for *mapDamage* originate from the specific organism to which they were mapped, or more likely originate from closely related species in the same genus or family, these damage plots show abundant terminal deamination patterns in both plant and animal sequences that are characteristic of ancient DNA. This again suggests that these molecules originate from the samples themselves and are not the result of contamination. Supplemental Figures 13 and 14 provide examples of on-target versus off-target mapping.

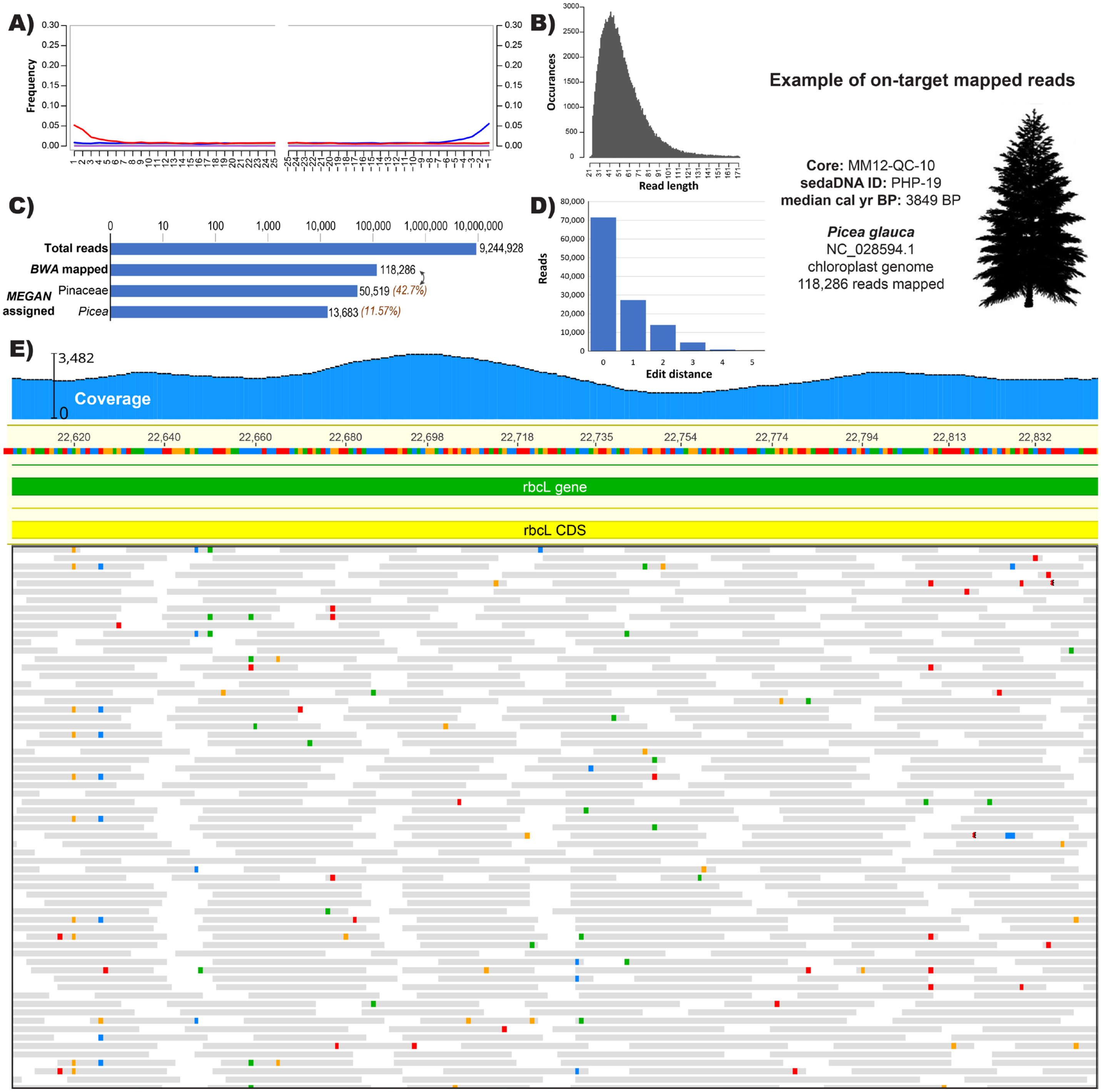

Supplementary Figure 13 On-target mapped reads for *Picea*

A) *mapDamage* fragment misincorporation plot[53] with a flat baseline. **B)** Ancient DNA typical fragment length distribution. **C)** Read mapping and binning specificity on a log scale. **D)** Edit distance distribution plot. Most reads have 0 polymorphisms relative to the mapped reference. **E)** BWA mapped reads in a portion of the rbcL gene with a coverage plot. Coloured squares indicate polymorphisms relative to the reference. Visualized in Geneious Prime (2019.2.3, www.geneious.com).

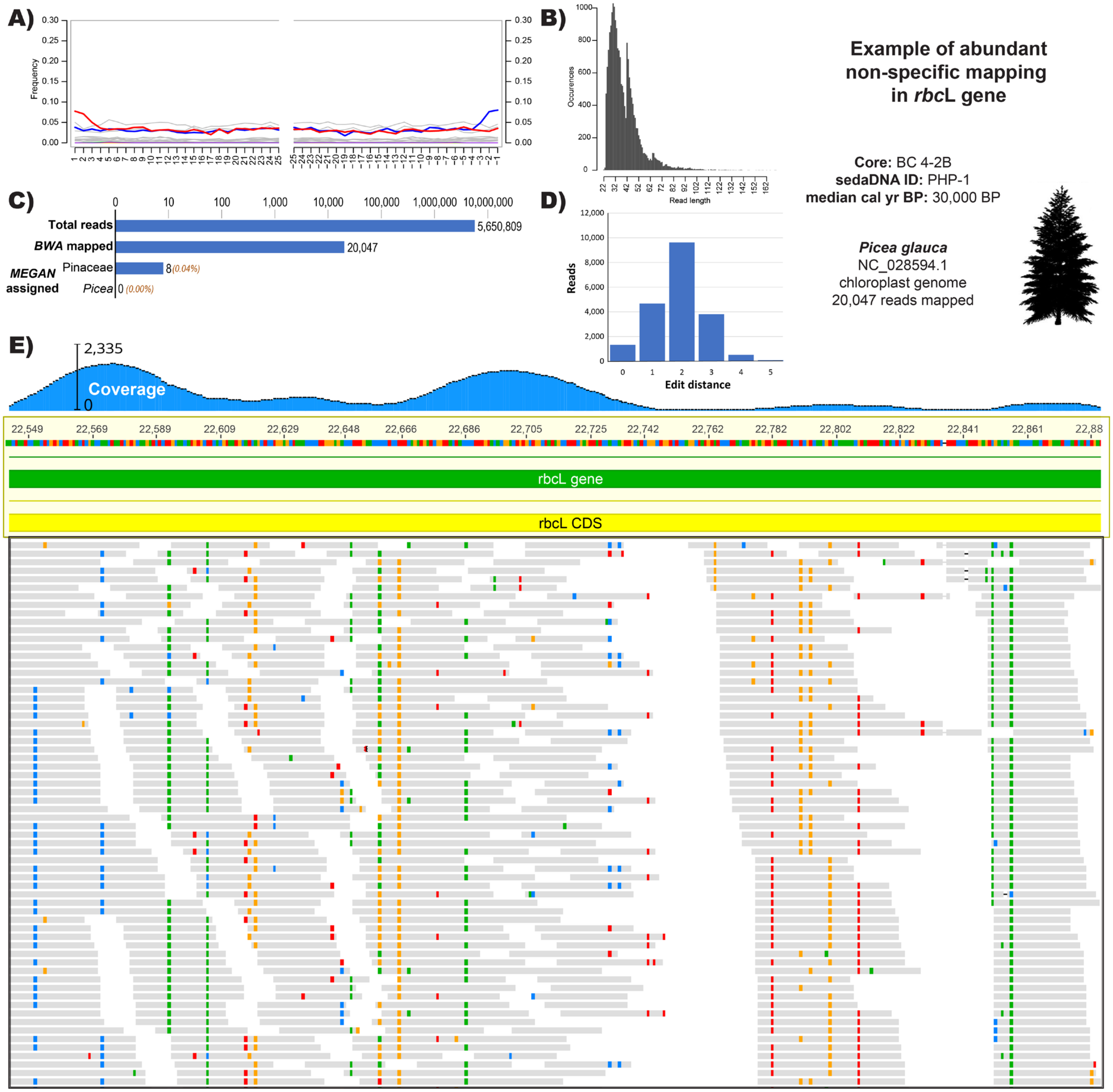

Supplementary Figure 14 Off-target mapped reads for *Picea*

A) *mapDamage* fragment misincorporation plot[53] with jagged baseline, but still containing clearly deaminated terminal bases. **B)** Bimodal fragment length distribution. **C)** Read specificity on a log scale. Note the significant drop in MEGAN read assignment compared with Supplemental Figure 13. **D)** Edit distance distribution plot. Note the increased average mismatches compared with Supplemental Figure 13. **E)** BWA mapped reads in a portion of the rbcL gene. Coloured squares indicate polymorphisms relative to the reference. This off-target mapping does not affect the *BLASTn*/*MEGAN* assignments. However, it does inflate the number of reads that map to a reference when assessing *mapDamage* plots. Regardless of off-target reads, there is still a pronounced signal of damage on the ends of these molecules even with polymorphic interior sites. The number of mapped reads here should not be used to infer taxonomic presence, but rather to confirm terminal base damage, supporting the argument that these molecules are ancient.

2.5.4 *mapDamage* plots

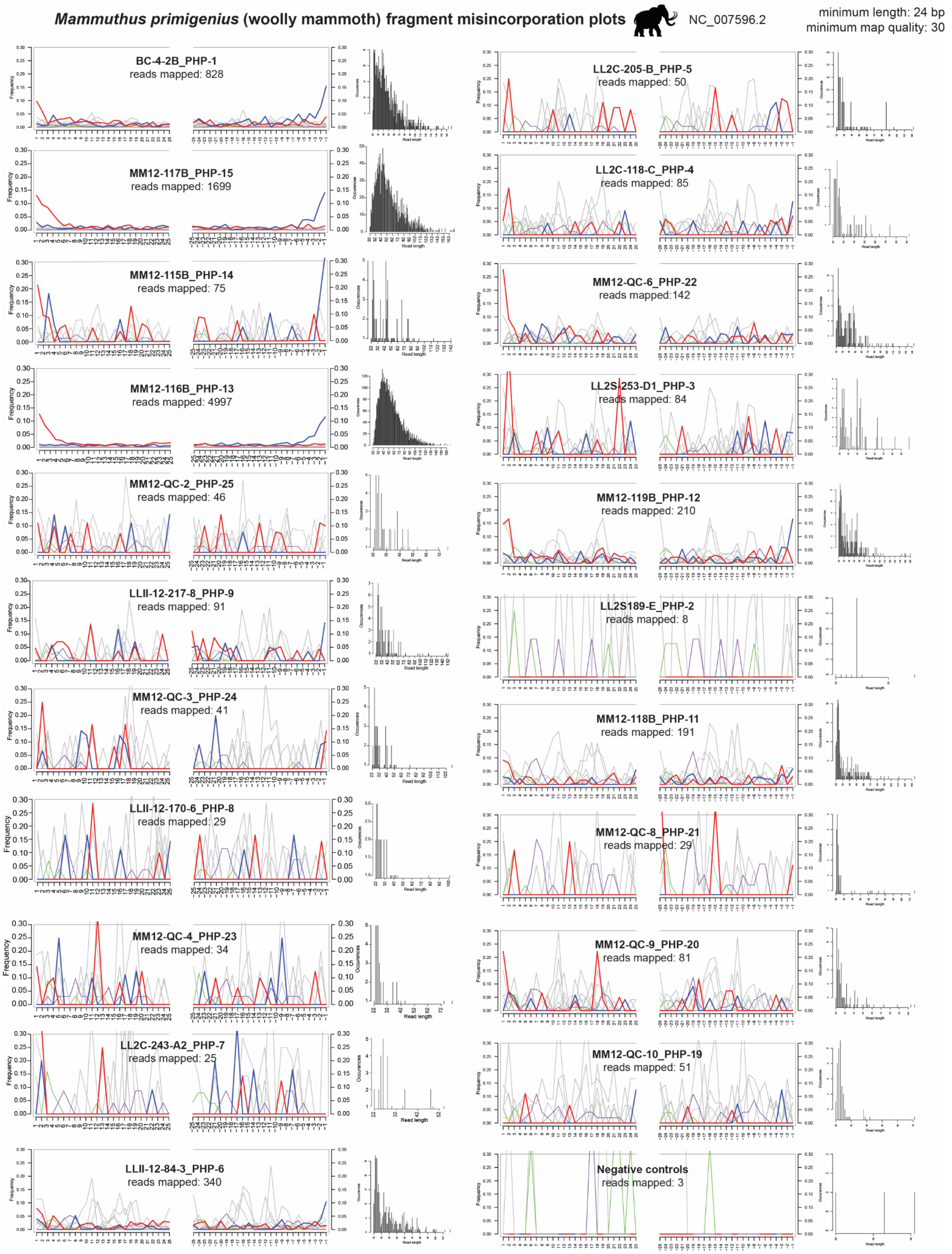

Supplementary Figure 15 *Mammuthus primigenius mapDamage* plots and fragment length distributions. https://www.ncbi.nlm.nih.gov/nuccore/NC_007596.2

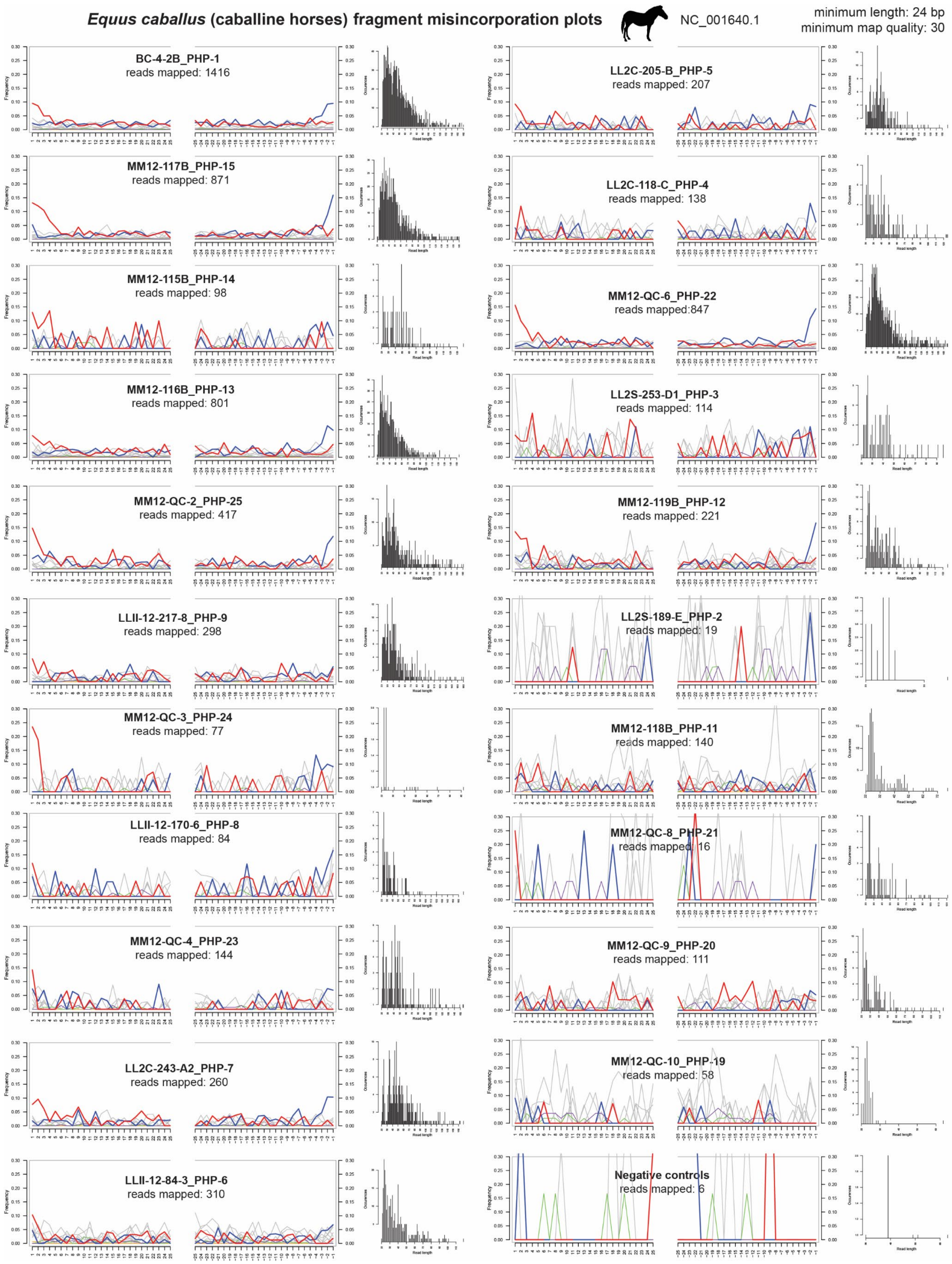

Supplementary Figure 16 *Equus mapDamage* plots and fragment length distributions. Jagged baseline caused by mapping to Eurasian *Equus caballus* whereas the *Equus caballus* lambei clade (Yukon wild horse) is a better mapping target.
https://www.ncbi.nlm.nih.gov/nuccore/NC_001640.1

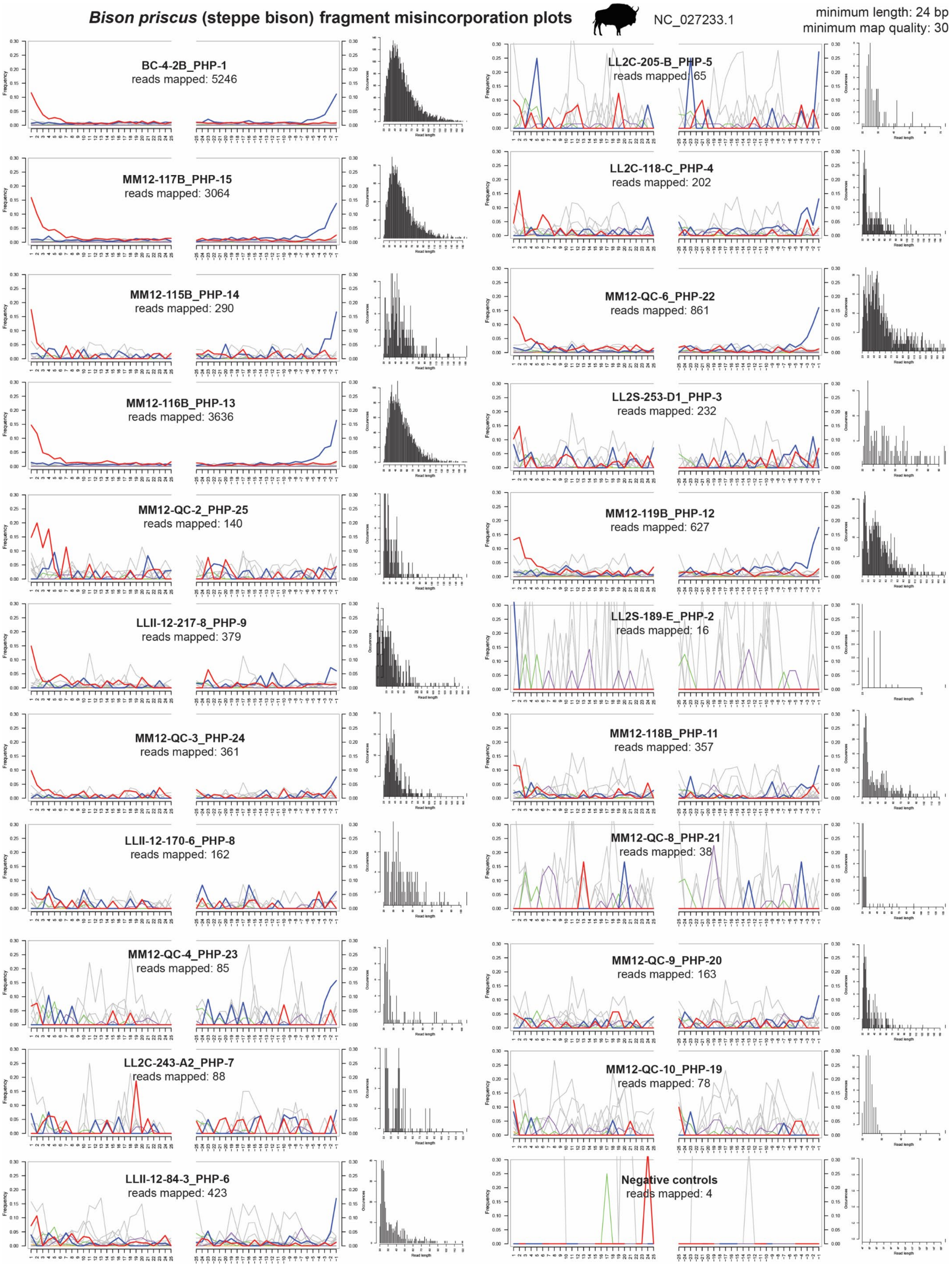

Supplementary Figure 17 *Bison priscus mapDamage* plots and fragment length distributions.
https://www.ncbi.nlm.nih.gov/nuccore/NC_027233

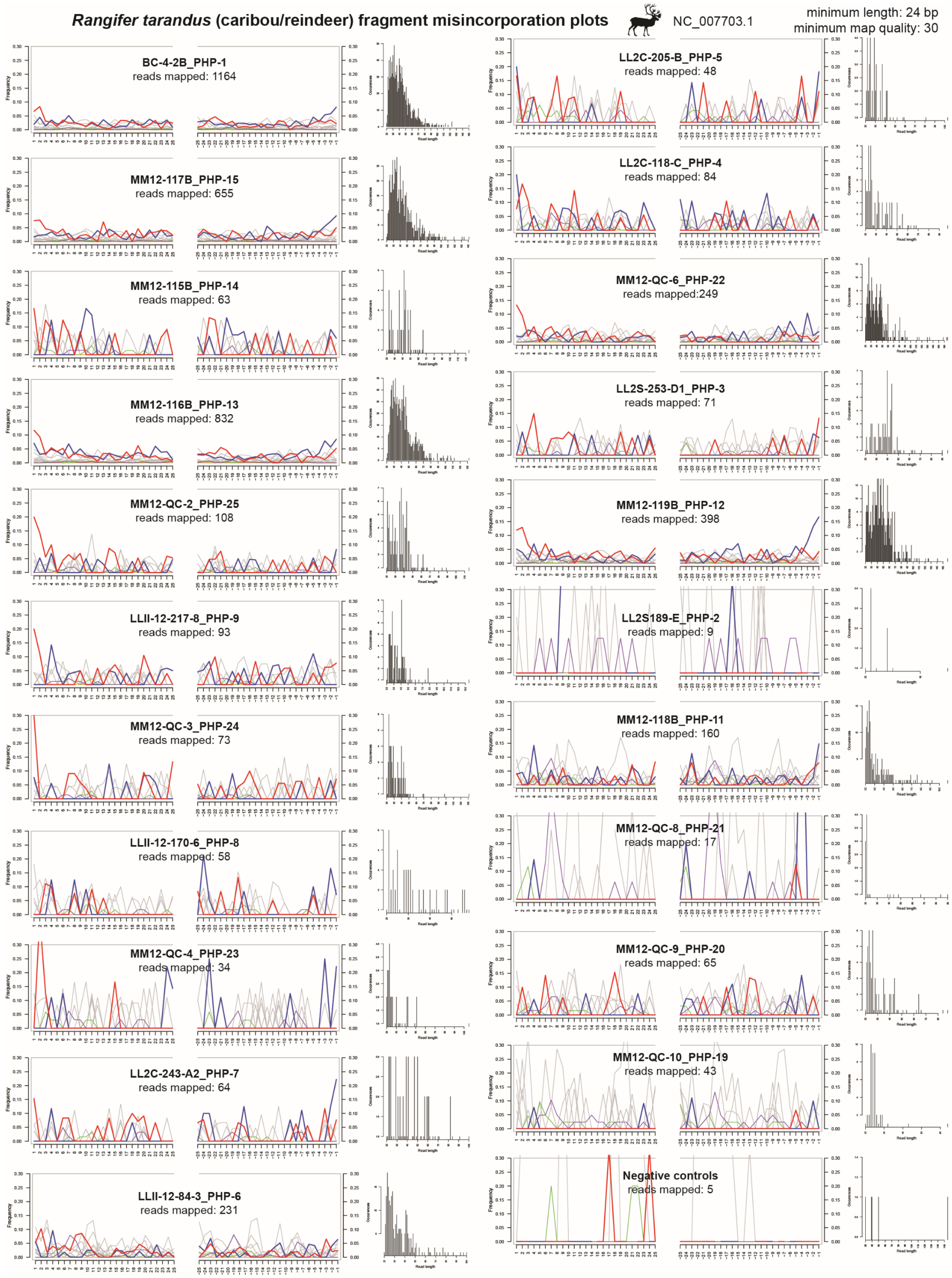

Supplementary Figure 18 *Rangifer tarandus mapDamage* plots and fragment length distributions. https://www.ncbi.nlm.nih.gov/nuccore/NC_007703

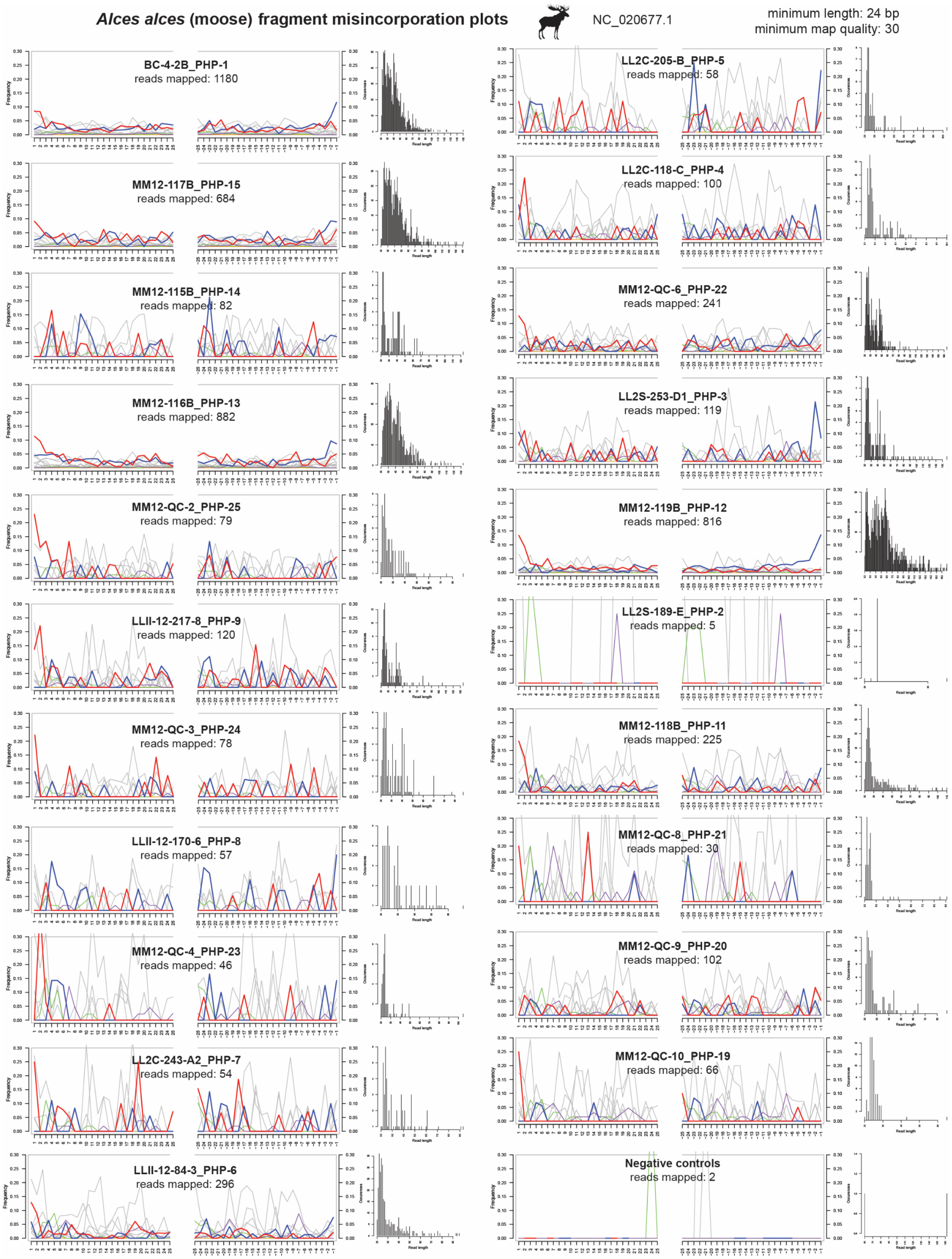

Supplementary Figure 19 *Alces alces mapDamage* plots and fragment length distributions. Abundant mapped reads in PHP-1/15/13 due to off-target mapping within Cervidae. https://www.ncbi.nlm.nih.gov/nuccore/NC_020677

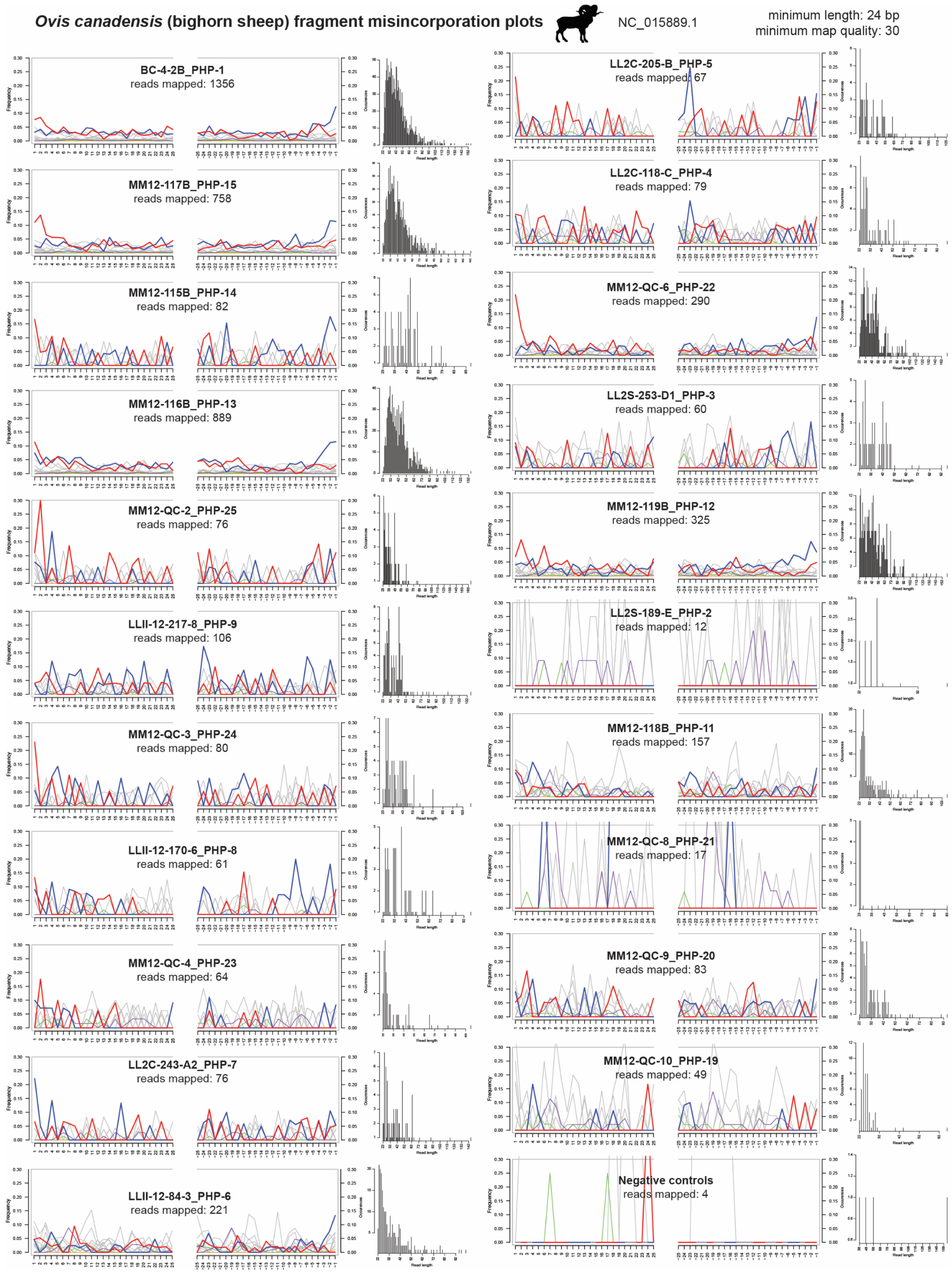

Supplementary Figure 20 *Ovis canadensis mapDamage* plots and fragment length distributions. https://www.ncbi.nlm.nih.gov/nuccore/NC_015889

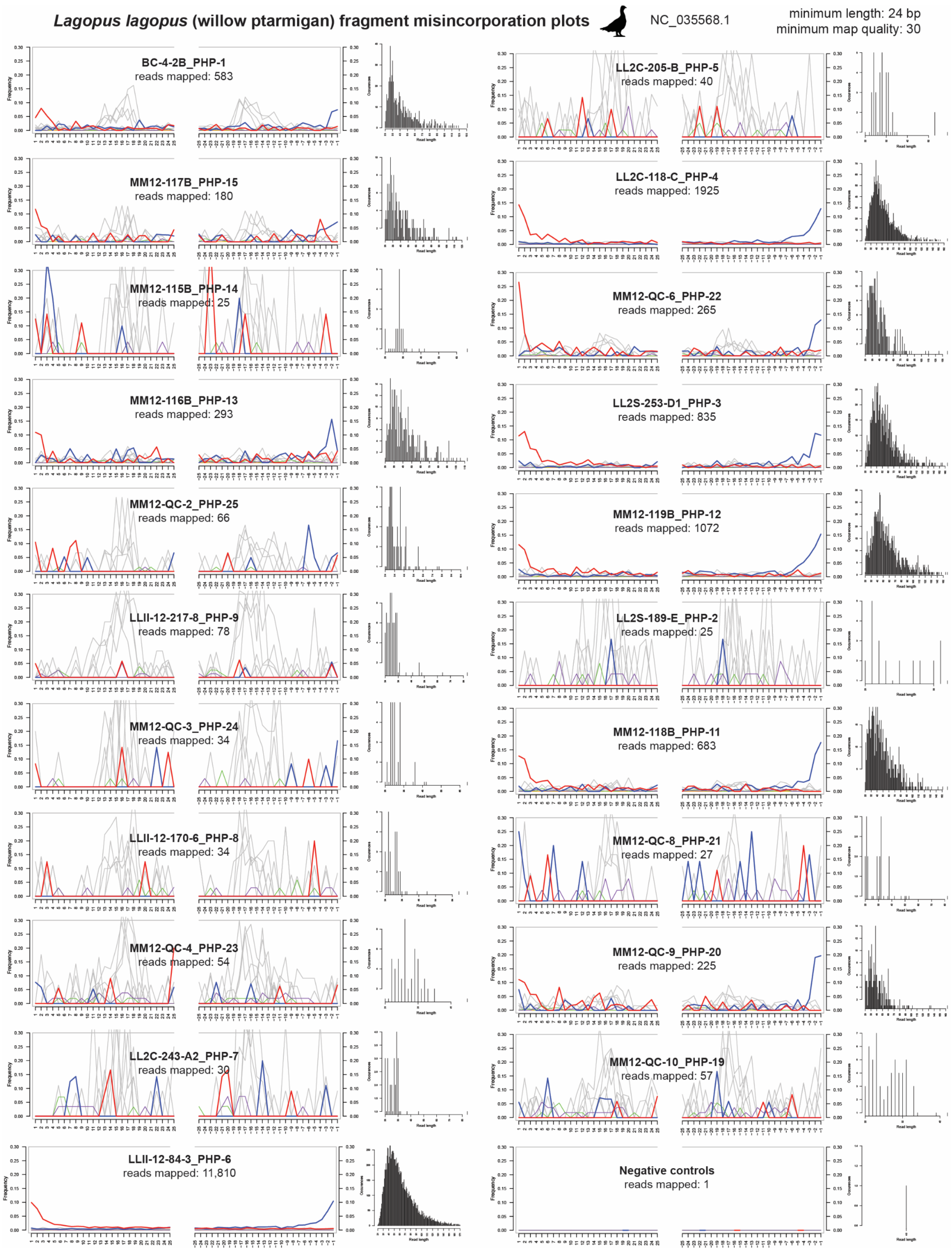

Supplementary Figure 21 *Lagopus lagopus mapDamage* plots and fragment length distributions. https://www.ncbi.nlm.nih.gov/nuccore/NC_035568

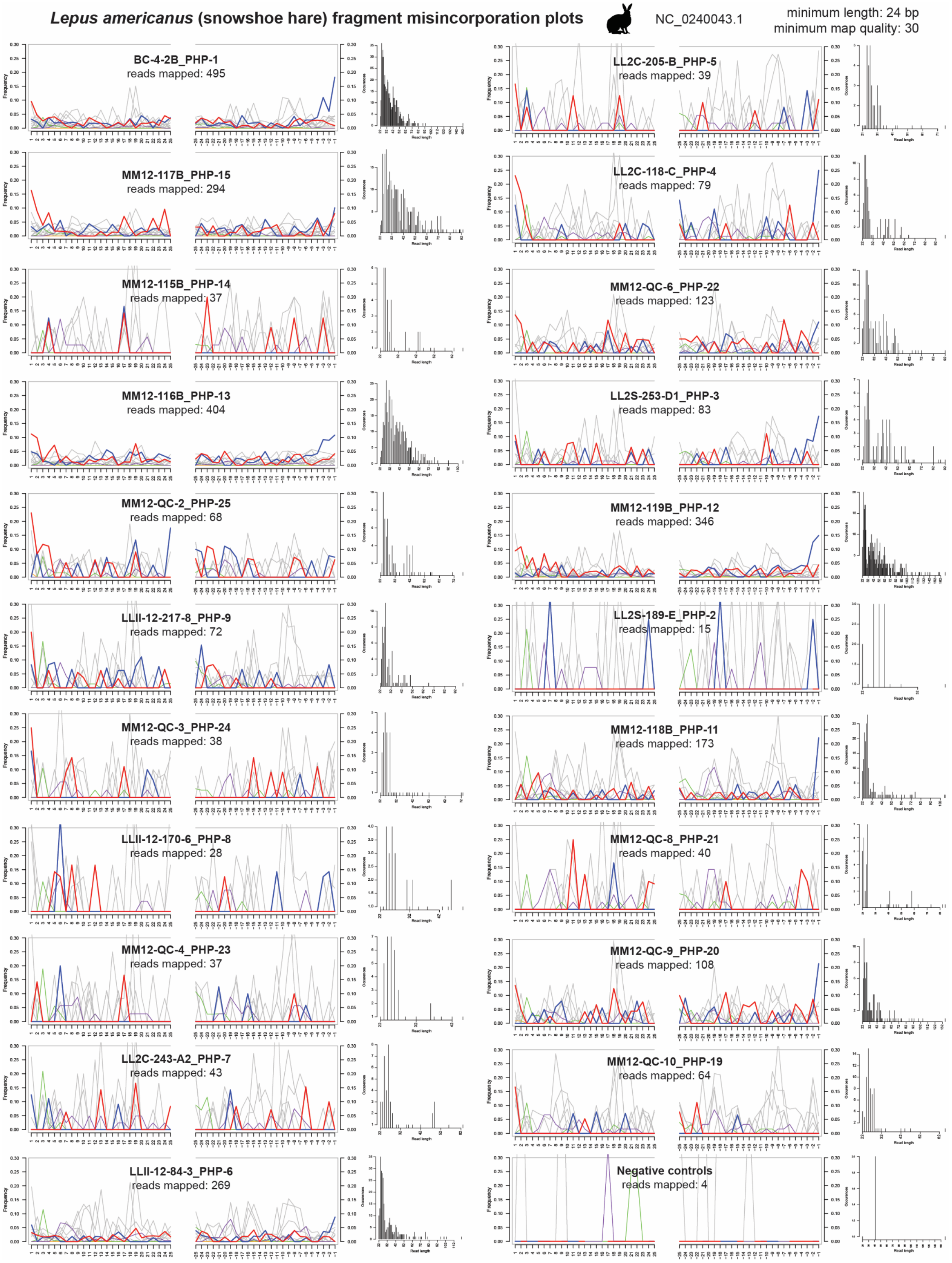

Supplementary Figure 22 *Lepus americanus mapDamage* plots and fragment length distributions. https://www.ncbi.nlm.nih.gov/nuccore/NC_024043.1

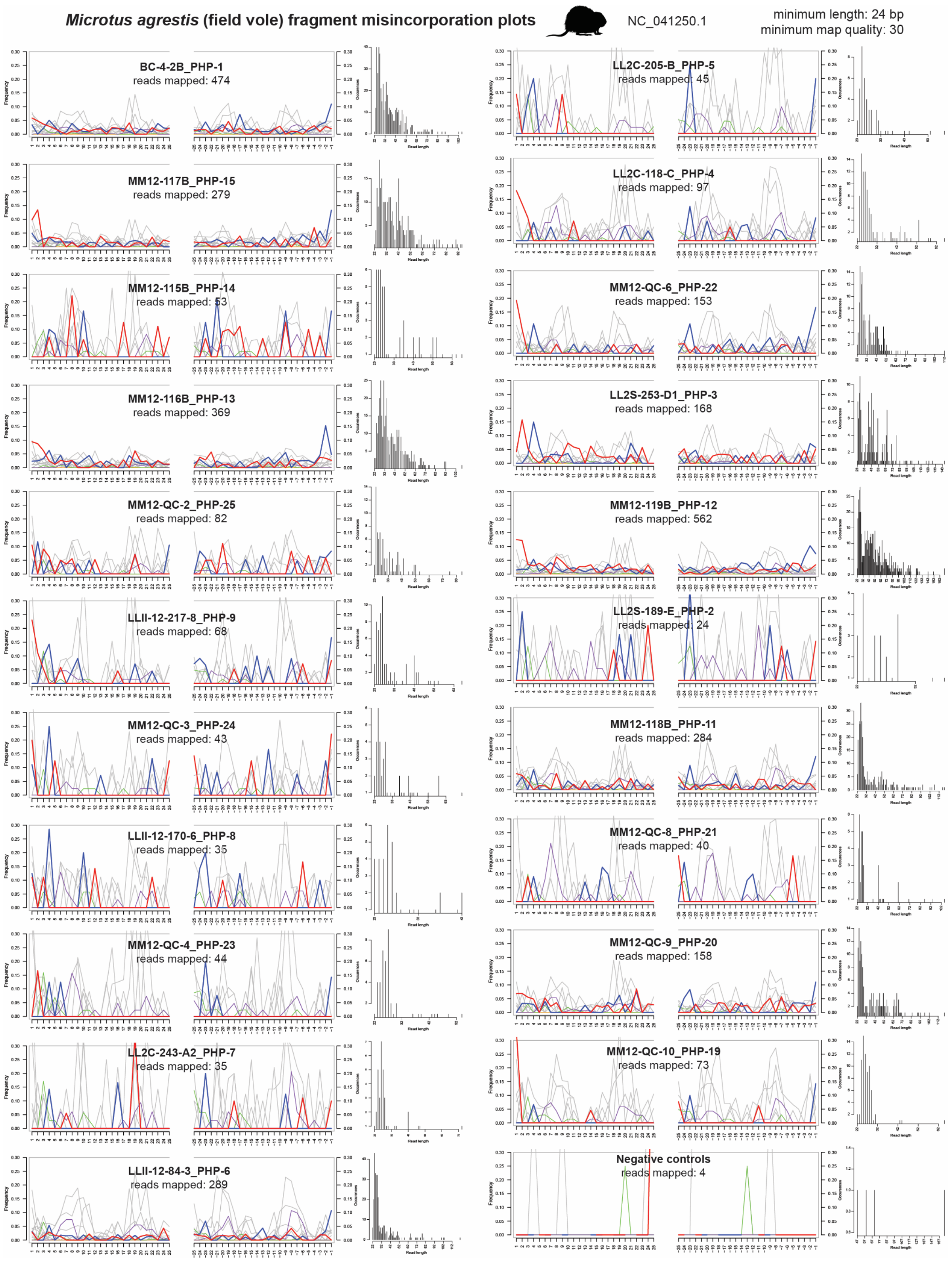

Supplementary Figure 23 *Microtus agrestis mapDamage* plots and fragment length distributions. https://www.ncbi.nlm.nih.gov/nuccore/NC_041250

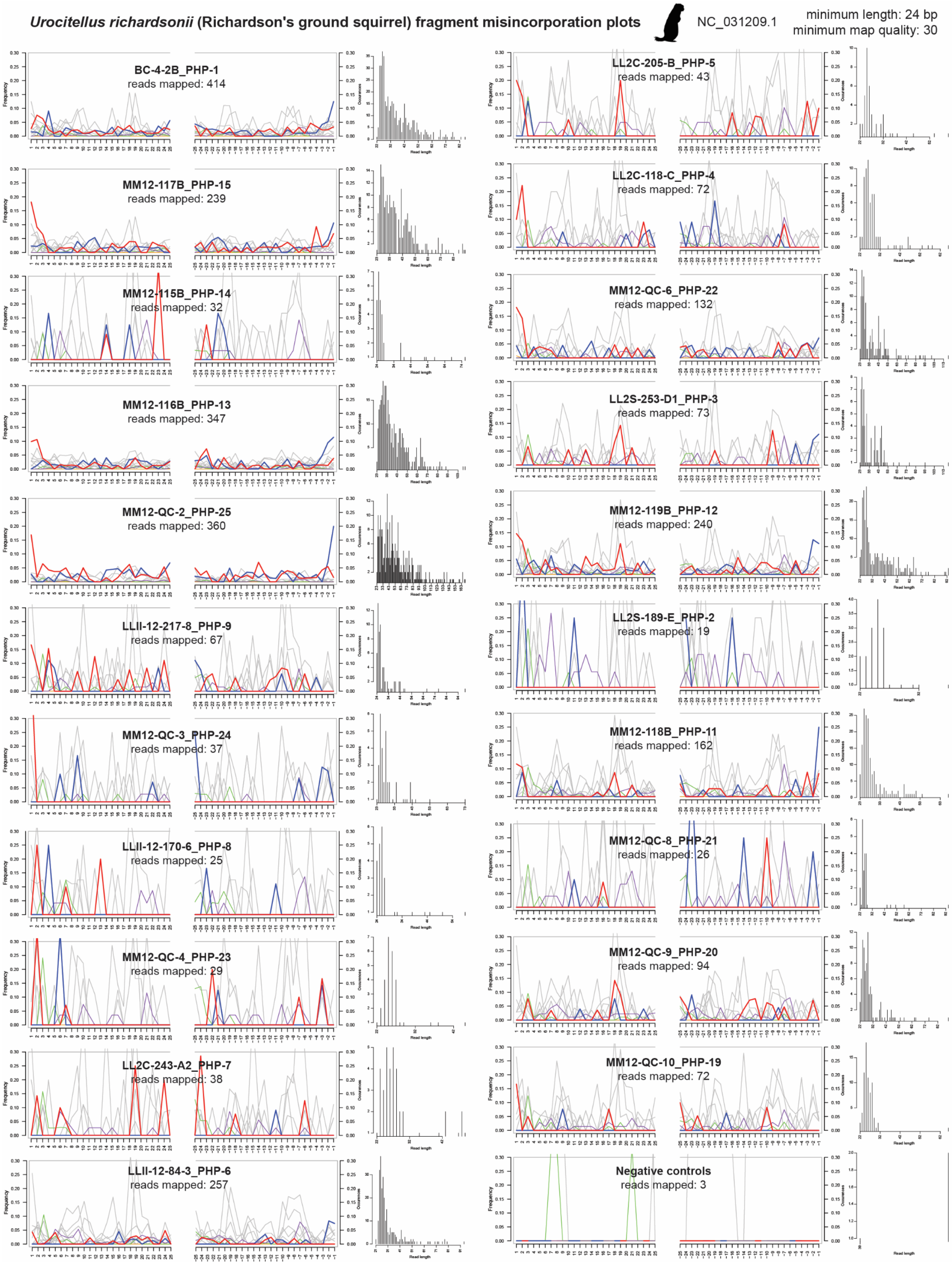

Supplementary Figure 24 *Uroitellus richardsonii mapDamage* plots and fragment length distributions. https://www.ncbi.nlm.nih.gov/nuccore/NC_031209

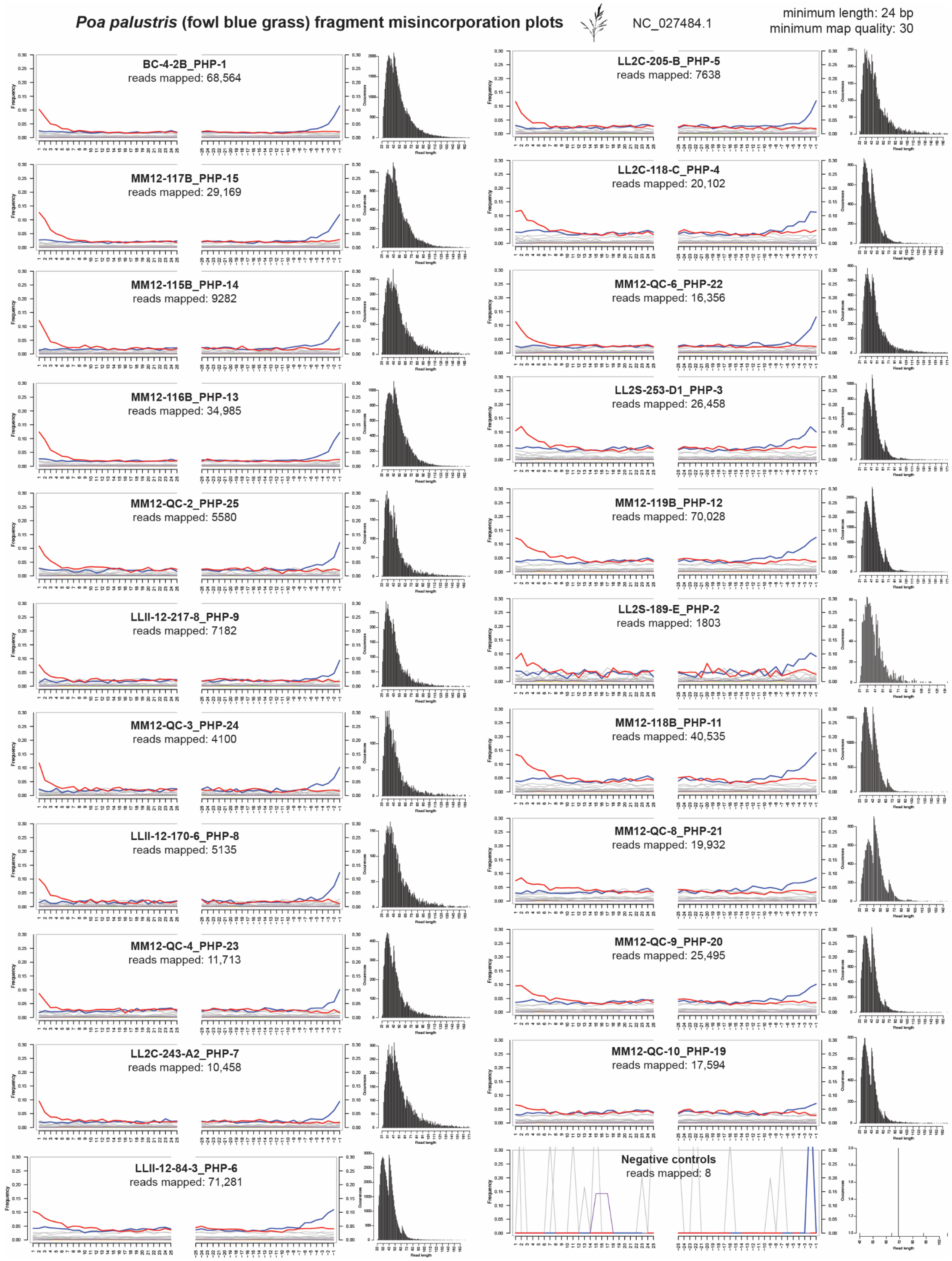

Supplementary Figure 25 *Poa palustris mapDamage* plots and fragment length distributions. https://www.ncbi.nlm.nih.gov/nuccore/NC_027484.1

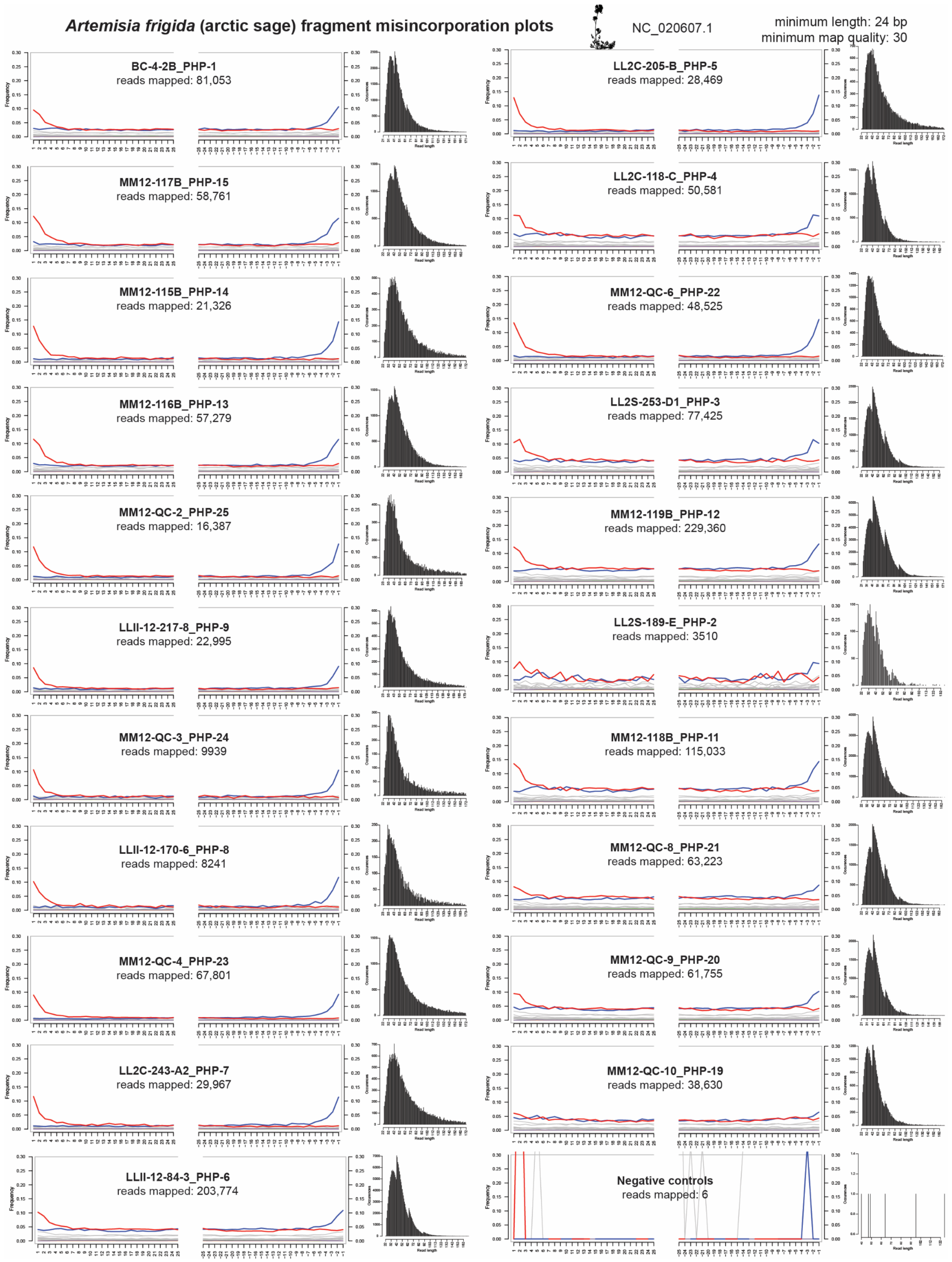

Supplementary Figure 26 *Artemisia frigida mapDamage* plots and fragment length distributions. https://www.ncbi.nlm.nih.gov/nuccore/NC_020607

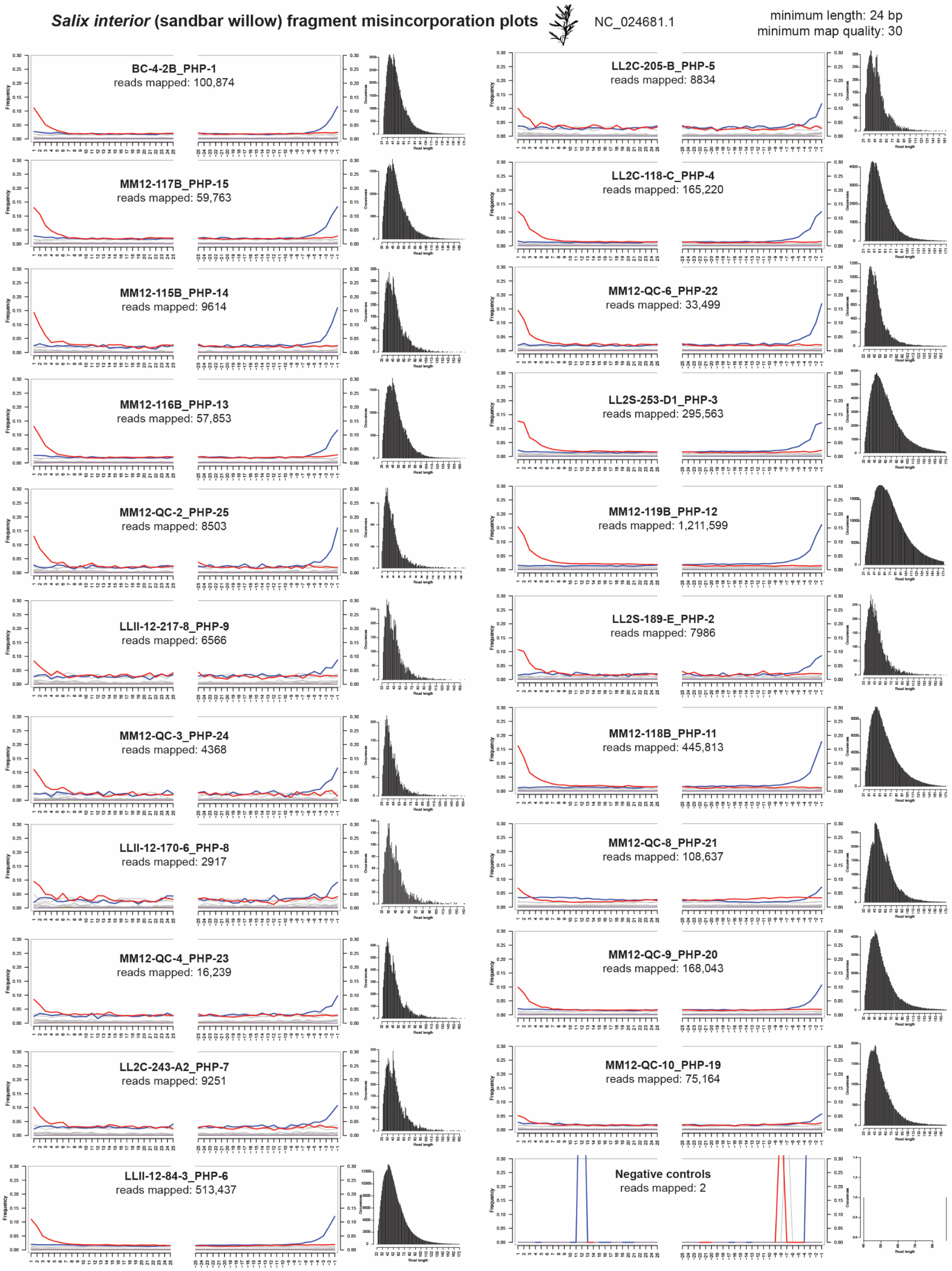

Supplementary Figure 27 *Salix interior mapDamage* plots and fragment length distributions. https://www.ncbi.nlm.nih.gov/nuccore/NC_024681

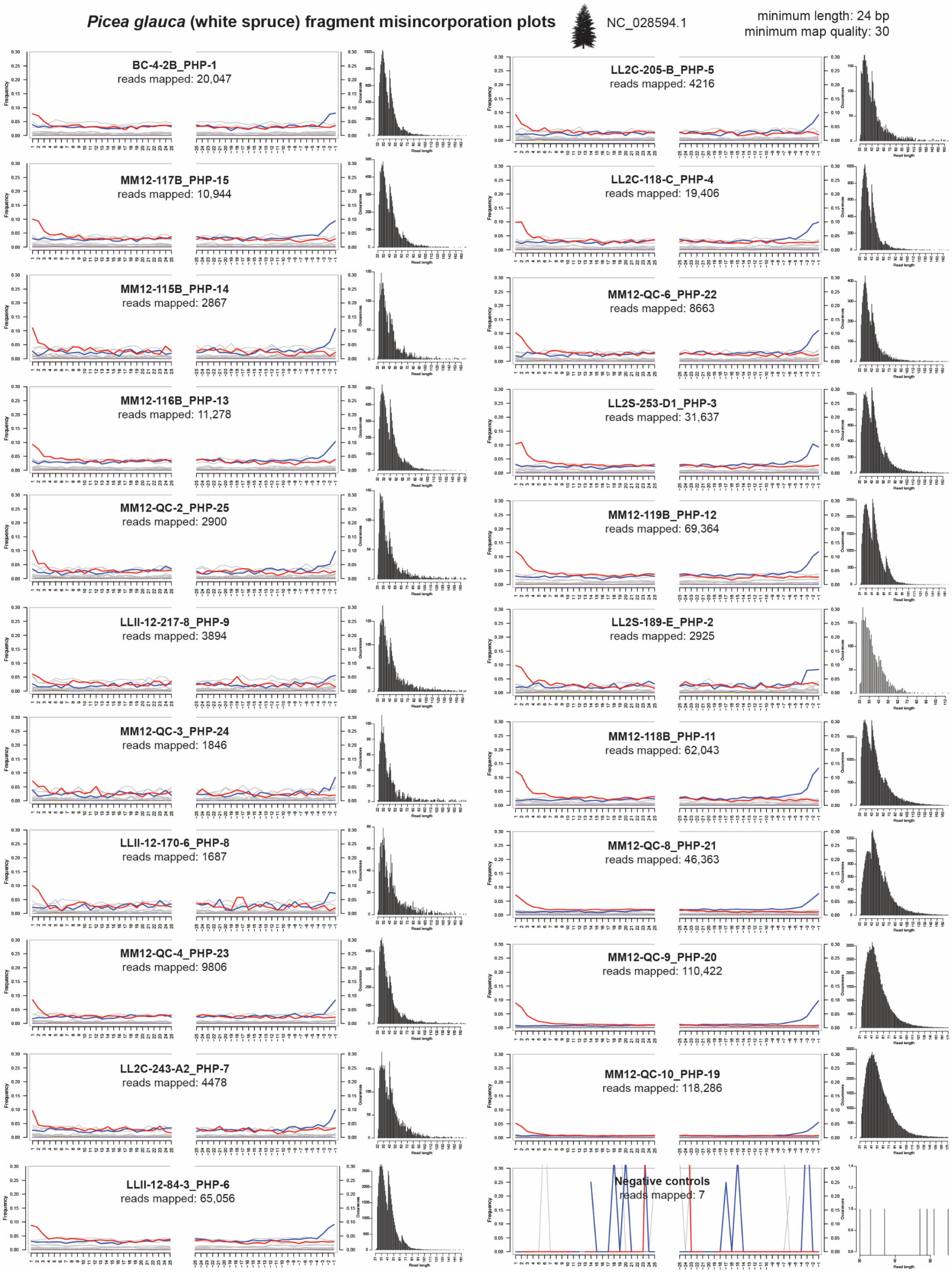

Supplementary Figure 28 *Picea glauca mapDamage* plots and fragment length distributions. https://www.ncbi.nlm.nih.gov/nuccore/NC_028594

2.5.5 Depurination and deamination rates

Several additional analyses were performed to assess whether the sequenced molecules had damage patterns characteristic of ancient DNA with a paired temporal or climatological signature. We expanded on the *mapDamage* analyses with reads mapped specifically to the *Salix interior* (sandbar willow) chloroplast reference genome (NC_024681.1 [https://www.ncbi.nlm.nih.gov/nuccore/NC_024681.1]) as Salicaceae was identified in all samples (mean = 160,733 reads mapped per library; min = 2917; max = 1,211,599; std dev = 269,302). To note, sandbar willow is likely not the specific species of willow represented in this dataset. This *Salix* chloroplast genome was chosen because it is a curated NCBI reference sequence, and as it has been demonstrated that *Salix* readily hybridizes within the genus, limiting phylogenetic resolution at the species rank[54].

We first assessed whether the mapping target was reasonably accurate by assessing the nucleotide mismatch distribution as denoted by a decreasing edit distance[55]. Mapped reads followed the expected pattern with the exception of samples PHP-5, 7–9, and 23 (Supplementary Figure 29). *Salix* was found to have the most consistent edit distance signal across time compared with alternative taxa within Poaceae and *Artemisia* despite some *Salix* mapped libraries with atypical distributions. The extent of DNA damage[56] was assessed using *mapDamage* v2.0[53]. Hydrolytic deamination, the erroneous replacement of nucleotides on the 5′ and 3′ ends of ancient DNA molecules, was assessed with fragment misincorporation plots (Supplementary Figure 30). Hydrolytic depurination, the fragmentation of DNA into short (20–60 bp) sections was assessed using fragment length distributions (FLD). These lengths will take the form of an exponential distribution and thus the depurination rate can be estimated as λ in the formula $y = N \cdot e^{-\lambda \cdot x}$ [57]. This parameter was estimated using a modified version of a previously published script from Kistler et al.[58].

We compared the rates of deamination and depuration against the modeled age estimates using multiple linear regressions by core sample and site. As the total amount of damage is expected to increase over time, a positive relationship between age and damage was hypothesized to be present. Further, mean temperature and annual precipitation have been shown to affect the rate of DNA damage [58]. While not exactly temperature or precipitation measurements, $\delta^{18}O$ can serve as an excellent proxy as it is affected by these two variables[59].

Single stranded deamination rates are roughly correlated with age, wherein older samples tend to have more deamination than those which are younger (Supplementary Figure 31). However, when comparing deamination rates to $\delta^{18}O$, we see a stronger correlation to temperature and hydrology wherein cores dating

to colder and drier periods have less deamination relative to those where the eDNA was released during warm and wet periods (Supplementary Figure 32). This is consistent with the results of Kistler et al.[58] who found that precipitation and temperature fluctuations contributed significantly to aDNA damage patterns, often irrespective of sample age. Depuration rates are also correlated with age wherein sedaDNA fragments tend to be shorter with older permafrost cores (Supplementary Figure 33). Deamination was found to be strongly correlated in a multivariate regression when the $\delta^{18}O$ values at the time of eDNA release was paired with modelled age (R^2_{adj} = 0.4219; p-value = 0.0112; F-statistic: 6.473 on 2 and 13 DF). Depurination rates by contrast were not significantly correlated with age and $\delta^{18}O$ (R^2_{adj} = 0.1695; p-value = 0.118; F-statistic: 2.531 on 2 and 13 DF).

The age and climate associated damage patterns observed here support the proposition that the bulk of eDNA recovered for our ecological reconstructions are roughly contemporaneous with the age-modelled dates for these cores. This supports both the timings of ecological turnover, and the late persistence of *Mammuthus* and *Equus* sedaDNA to the early and mid-Holocene.

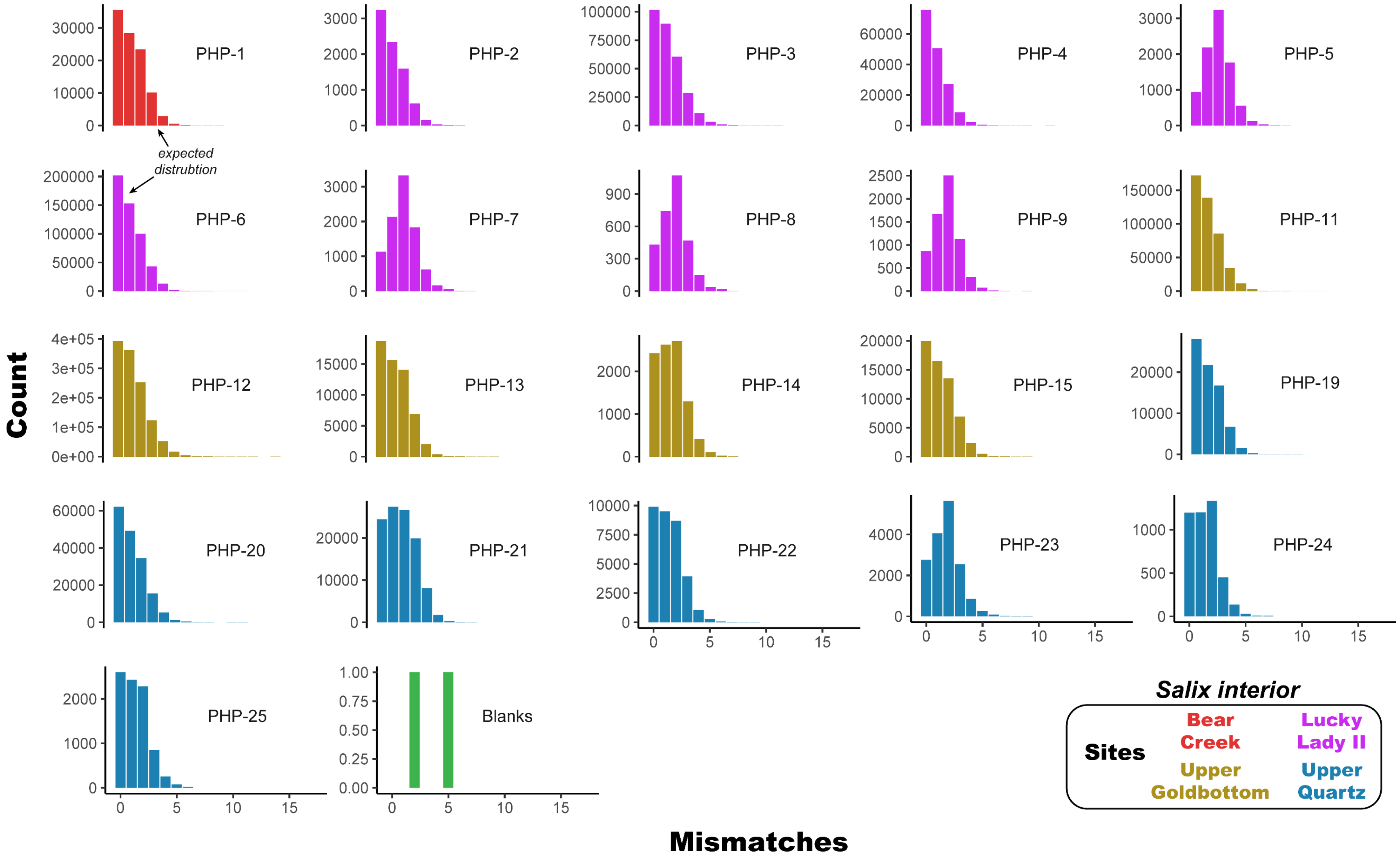

Supplementary Figure 29 Plotted edit distances for reads mapped to the *Salix interior* chloroplast reference genome (NC_024681.1). PHP-5,7–9, and 23 have atypical edit distance distributions indicating that there is abundant off-target mapping to this reference, which may impact deamination/depuration rate estimates.

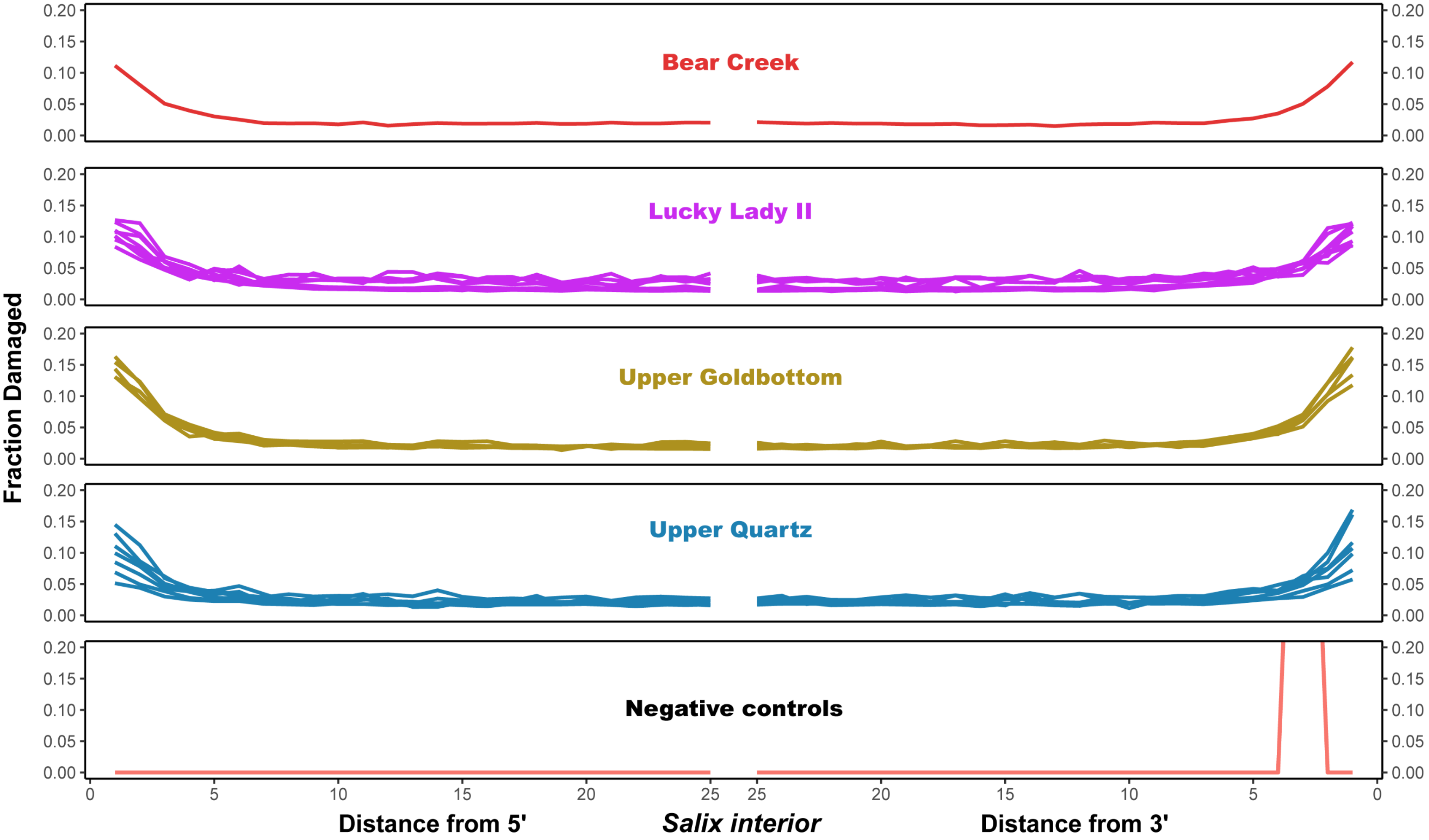

Supplementary Figure 30 Fragment misincorporation plots to confirm the presence of hydrolytic deamination for reads mapped to the *Salix interior* chloroplast reference genome (NC_024681.1). All libraries show characteristic ancient DNA damage patterns.

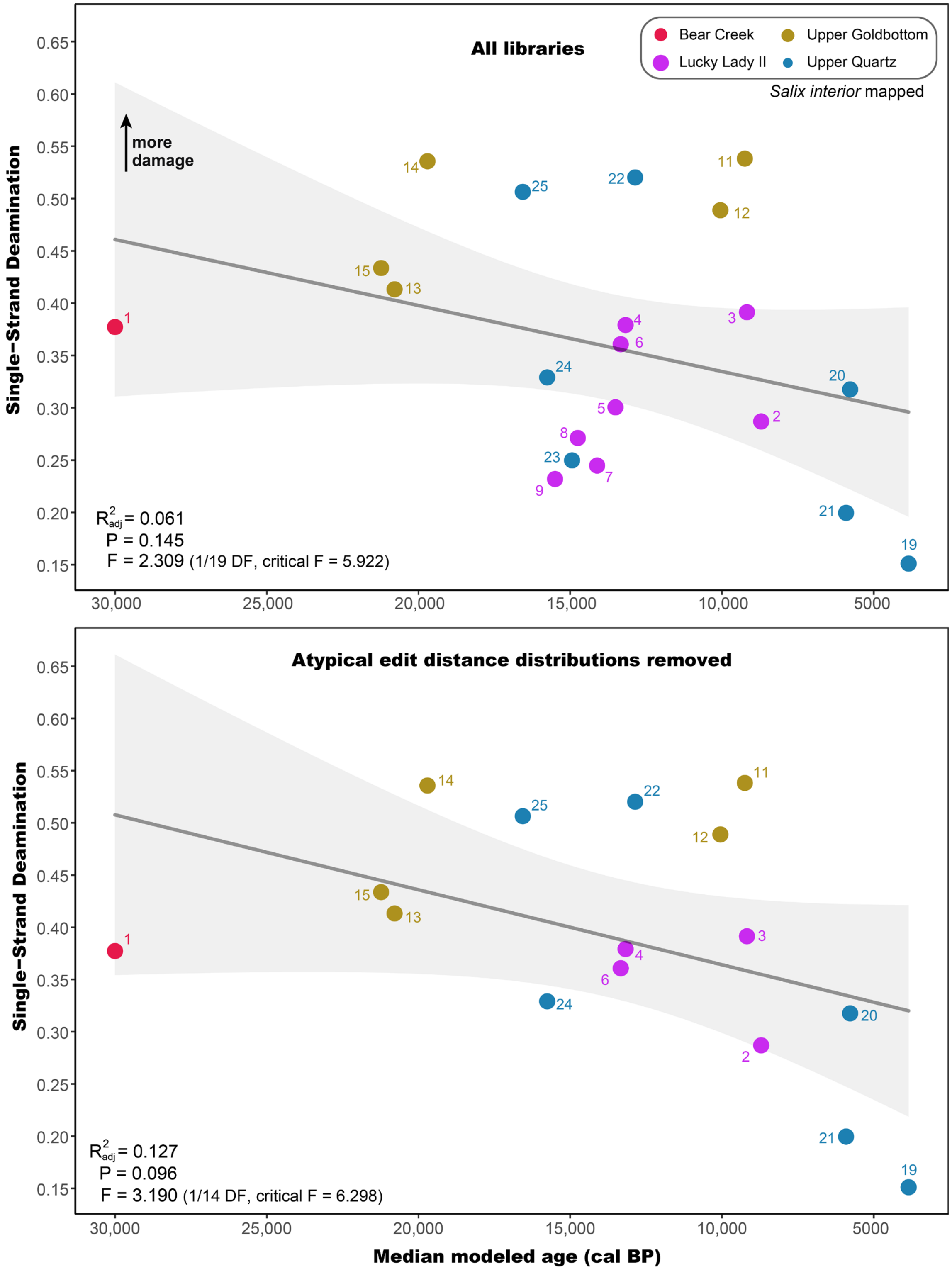

Supplementary Figure 31 Deamination rate of *Salix interior* mapped reads against time. **A)** All libraries included (n=21). **B)** Reads with atypical edit distance distributions removed (n=16). Shaded region = 95% confidence interval. P-values from a two-sided F-test of the linear regression model.

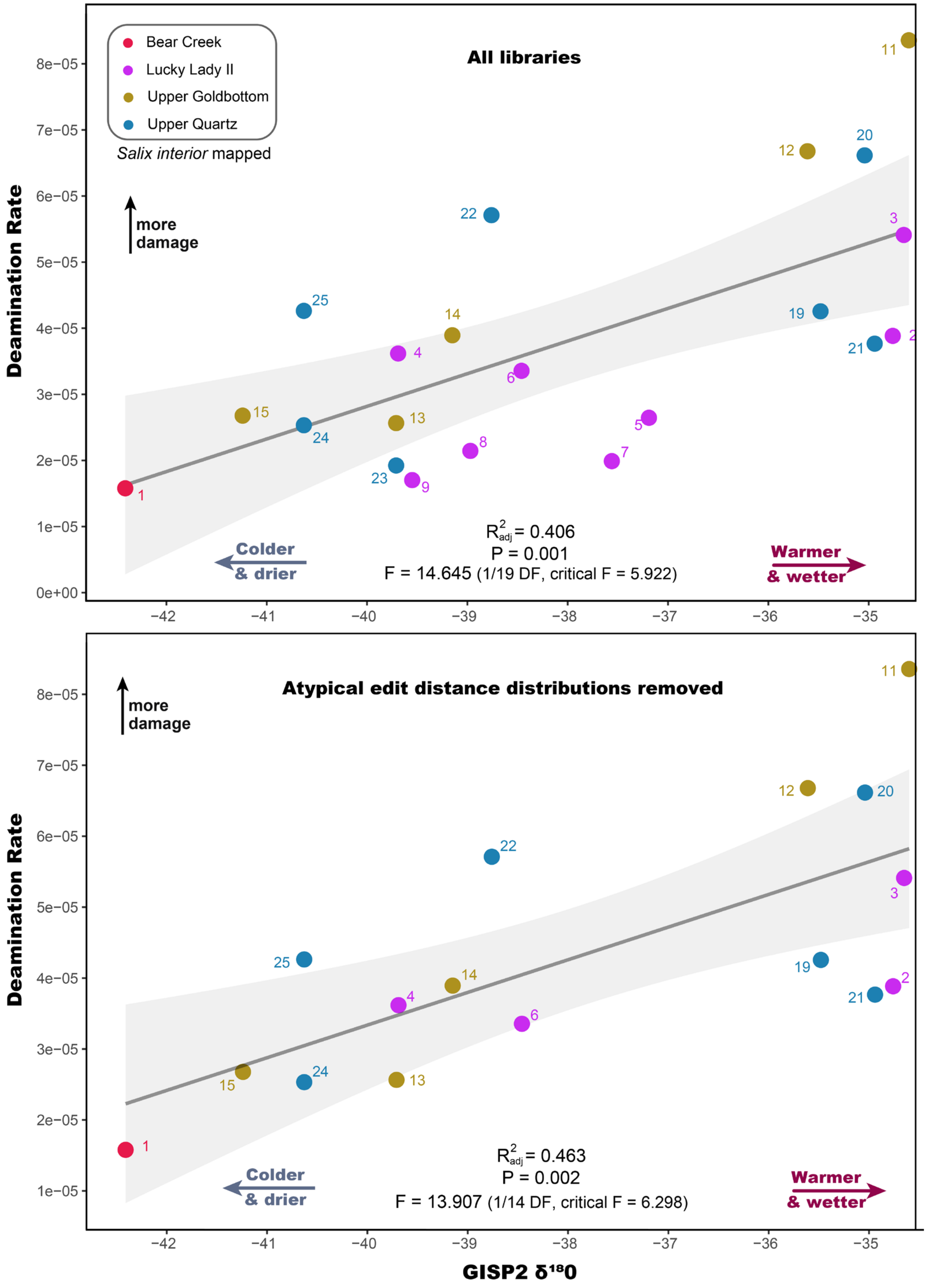

Supplementary Figure 32 Deamination rate of *Salix interior* mapped reads compared with the temporally nearest Greenland Ice Sheet Project 2 (GISP2) oxygen-18 isotope value [62]. A) All libraries included (n=21). B) Atypical edit distance libraries removed (n=16). Shaded region = 95% confidence interval. P-values from a two-sided F-test of the linear regression model.

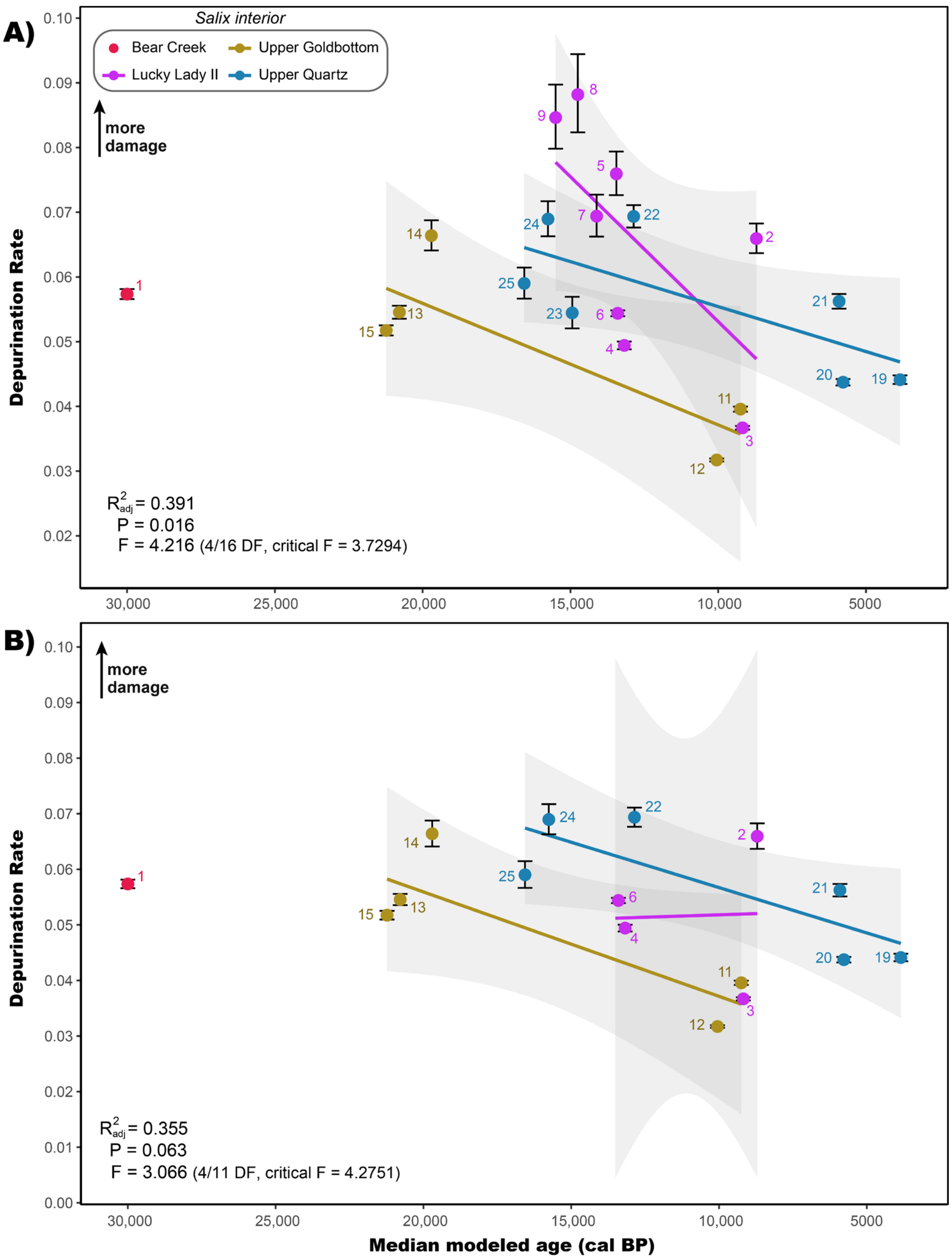

Supplementary Figure 33 Depurination rate of *Salix interior* mapped reads against time. A) All libraries included (n=21). B) Reads with atypical edit distance distributions removed (n=16). Shaded region = 95% confidence interval. P-values from a two-sided F-test of the linear regression model. Note: PHP-2 is an outlier in the metagenomic data with exceptionally poor sedaDNA preservation (no faunal DNA were identified). When this library is removed from panel B, the model becomes significantly correlated (R^2_{adj} = 0.620, P = 0.007, F = 6.716 (4/10 DF, critical F = 4.468).

2.5.6 Taxonomic binning replicate comparison between MEGAN and PIA

This section includes a taxonomic binning breakdown of subsampled replicates across batches. We observe a consistency between replicates in terms of their taxonomic compositions. We also observe that while the *PIA* taxonomic binning approach my be effective in reducing the potential for false-positive hits, it is too conservative to be of direct utility with this dataset. We utilized the top 600 *BLASTn* hits (100 more hits retained than recommended by the *PIA* developers[60], but still observe in many instances that all 600 hits are for the same or very closely related organisms. In these instances, *PIA* drops the read entirely assuming that this portion of the database is lacking in references aside from a set of over-represented 'oasis' taxa, irrespective of the taxonomic accuracy. Overall, this results in substantial data loss compared with *MEGAN*. We suspect that this limitation could be avoided by returning as many as the top 1500+ hits, but our blast files were already almost unwieldy with 600 hits. Some individual blast files are over 200 gigabytes, and in total the post-filtering dataset is approximately 3 terabytes in size. These limitations could likely be avoided by utilizing a taxonomically non-redundant or otherwise manually curated reference database, but these options may bias taxonomic binning to only organisms one expects to find. A *BLASTn* or similar alignment approach that had the option to collapse accession hits based on Taxonomy ID up to a certain threshold and could instead return the top 500+ Taxonomic ID hits would help alleviate the challenges of metagenomic noise observed here and in Murchie et al.[48]. We also observed that there is utility in an ensemble *PIA* and *MEGAN* approach insofar as it provides a sanity check for taxonomic binning of rare or unexpected taxa. The flexibility and power of *MEGAN* with its customizable LCA and comprehensive GUI was found to be highly effective with this dataset, with limitations in *BLASTn* customization being the limiting factor.

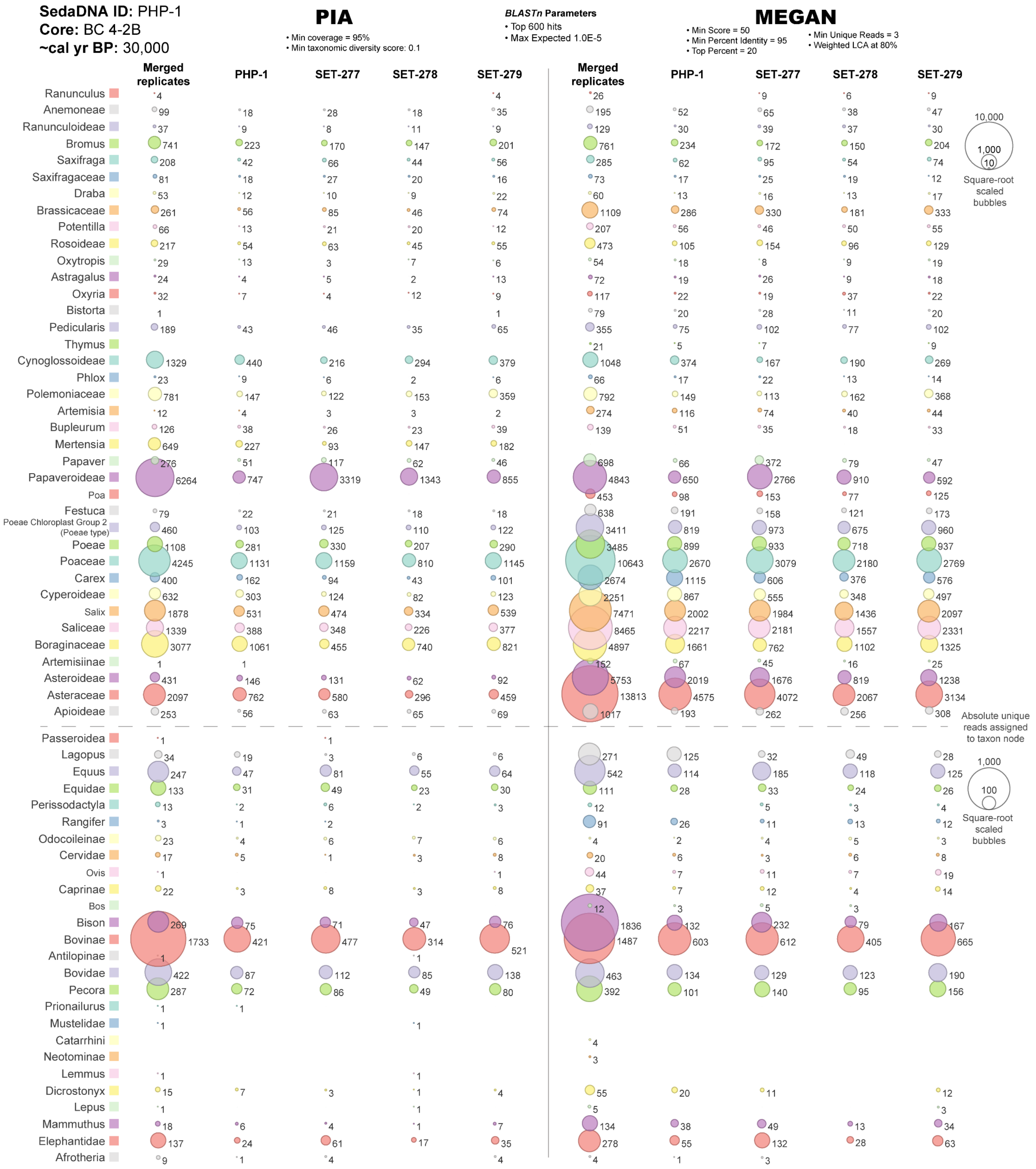

Supplementary Figure 34 PHP-1 replicates comparison with *PIA* and *MEGAN* taxonomic binning. Select nodes depicted.

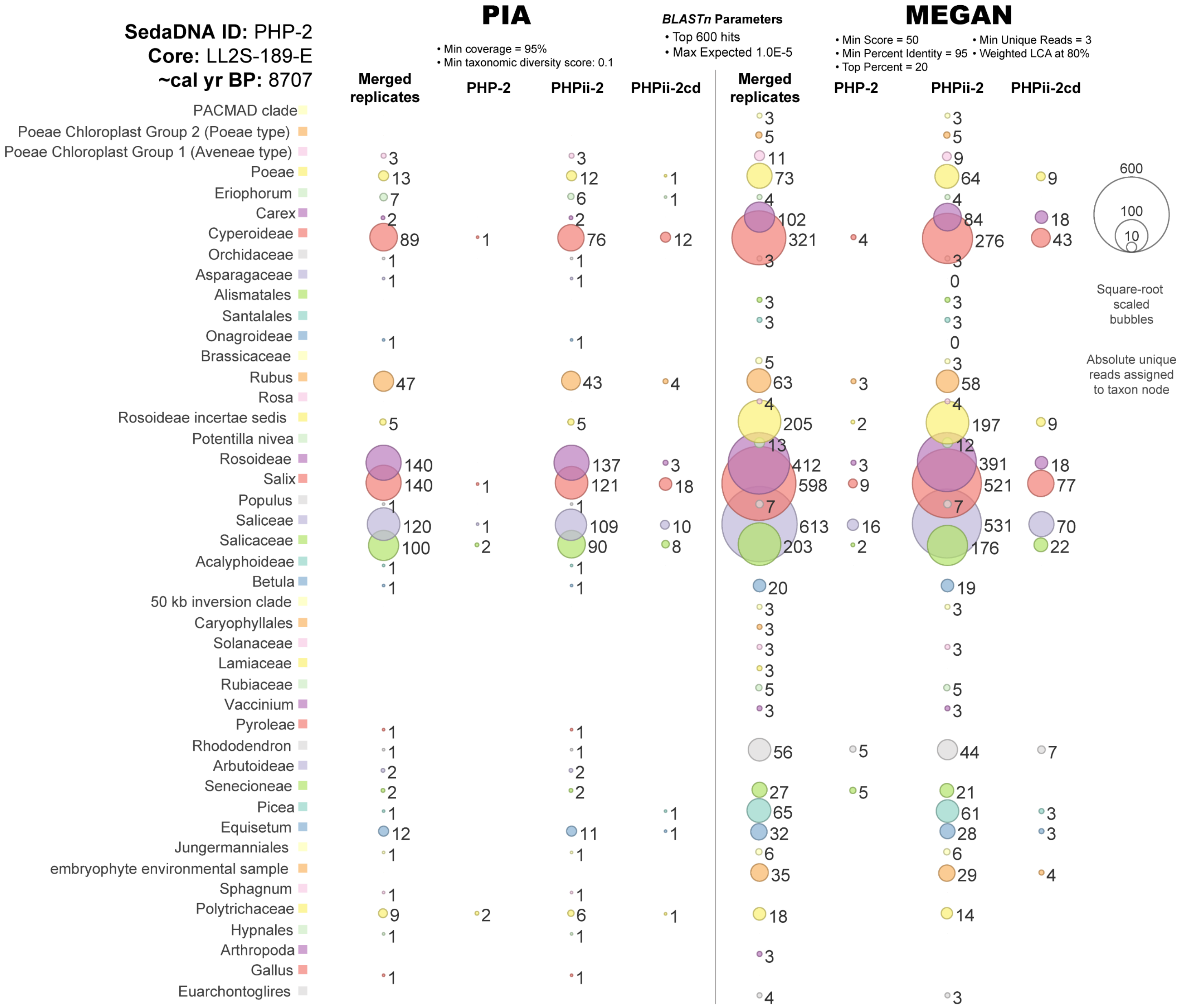

Supplementary Figure 35 PHP-2 replicates comparison with *PIA* and *MEGAN* taxonomic binning. Select nodes depicted.

SedaDNA ID: PHP-3
Core: LL2S-253-D1
~cal yr BP: 9177

PIA

- Min coverage = 95%
- Min taxonomic diversity score: 0.1

BLASTn **Parameters**

- Top 600 hits
- Max Expected 1.0E-5

MEGAN

- Min Score = 50
- Min Percent Identity = 95
- Top Percent = 20
- Min Unique Reads = 3
- Weighted LCA at 80%

Taxon	PIA Merged replicates	PIA PHPii-3	PIA PHPii-3cd	MEGAN Merged replicates	MEGAN PHPii-3	MEGAN PHPii-3cd
Papaver	6		6	13		12
Papaveroideae	61	5	56	53	11	47
Poa				24		22
Poeae Chloroplast Group 2 (Poeae type)	23	5	18	96	17	80
Poeae	66	13	53	348	70	278
Pooideae	52	8	44	437	85	353
Carex	900	99	801	7941	1114	6823
Cyperoideae	1069	128	941	4936	847	4099
Ribes	100	15	85	100	15	84
Epilobium	15	8	7	107	51	76
Potentilla	27	3	24	47	6	41
Fragariinae	4	1	3	338	78	261
Salix	14647	2737	11910	44032	9598	36457
Populus	3288	572	2716	14890	1273	11702
Fagaceae	6	1	5	707	159	560
Betula	473	86	387	2529	546	1993
Alnus	14	4	10	44	10	34
Betulaceae	7037	1331	5706	7166	1372	5800
Lupinus	124	24	100	128	18	108
Pedicularis	22	5	17	48	14	34
Thymus				130	25	101
Vaccinium				6		5
Vaccinieae	26	1	25	31	3	29
Vaccinioideae	43	5	38	80	14	70
Pyrola	150	30	120	460	60	344
Rhododendron				494	122	396
Ericoideae	48	12	36	221	67	168
Arctous	9	2	7	344	15	298
Arbutoideae	1750	279	1471	886	164	746
Viburnum	400	59	341	242	34	208
Senecioneae	80	16	64	328	62	267
Artemisia	3		3	43	7	36
Anthemideae	45	7	38	201	46	155
Pinus	2		2	4		3
Picea	1		1	1015	102	913
Abies				4		3
Pinaceae	13	1	12	3847	378	3471
Equisetum	6292	1107	5185	17228	3006	13841
Equisetaceae	5386	959	4427	19	25	29
Turdus	2		2	9		9
Lagopus	117	11	106	679	70	589
Tetraoninae	64	4	60	4	4	6
Phasianidae	64	8	56	12	5	18
Equus	4	3	1	11	8	3
Equidae	2	2		2		2
Alces				15		11
Odocoileinae	3		3	4		4
Cervidae	8		8	6		6
Bison	4		4	7		5
Bovinae	21	4	17	29	8	23
Bovidae	4	3	1	11	6	5
Pecora	8	1	7	14	4	14
Homo				4		3
Marmotini	1	1				
Murinae	1		1			
Myodes	1		1			
Microtus	15	2	13	46	6	36
Dicrostonyx	1		1	7		7
Arvicolinae	48	6	42	44	9	36
Cricetidae	14	3	11	15	1	15
Muroidea	11	1	10	22	2	21
Lepus	1		1	16	4	12
Mammuthus	1		1	3		3
Elephantidae	2		2	6		6

Bubble legend (plants): 40,000; 10,000; 1,000 — Square-root scaled bubbles

Absolute unique reads assigned to taxon node

Bubble legend (animals): 600; 100; 10 — Square-root scaled bubbles

Supplementary Figure 36 PHP-3 replicates comparison with *PIA* and *MEGAN* taxonomic binning. Select nodes depicted.

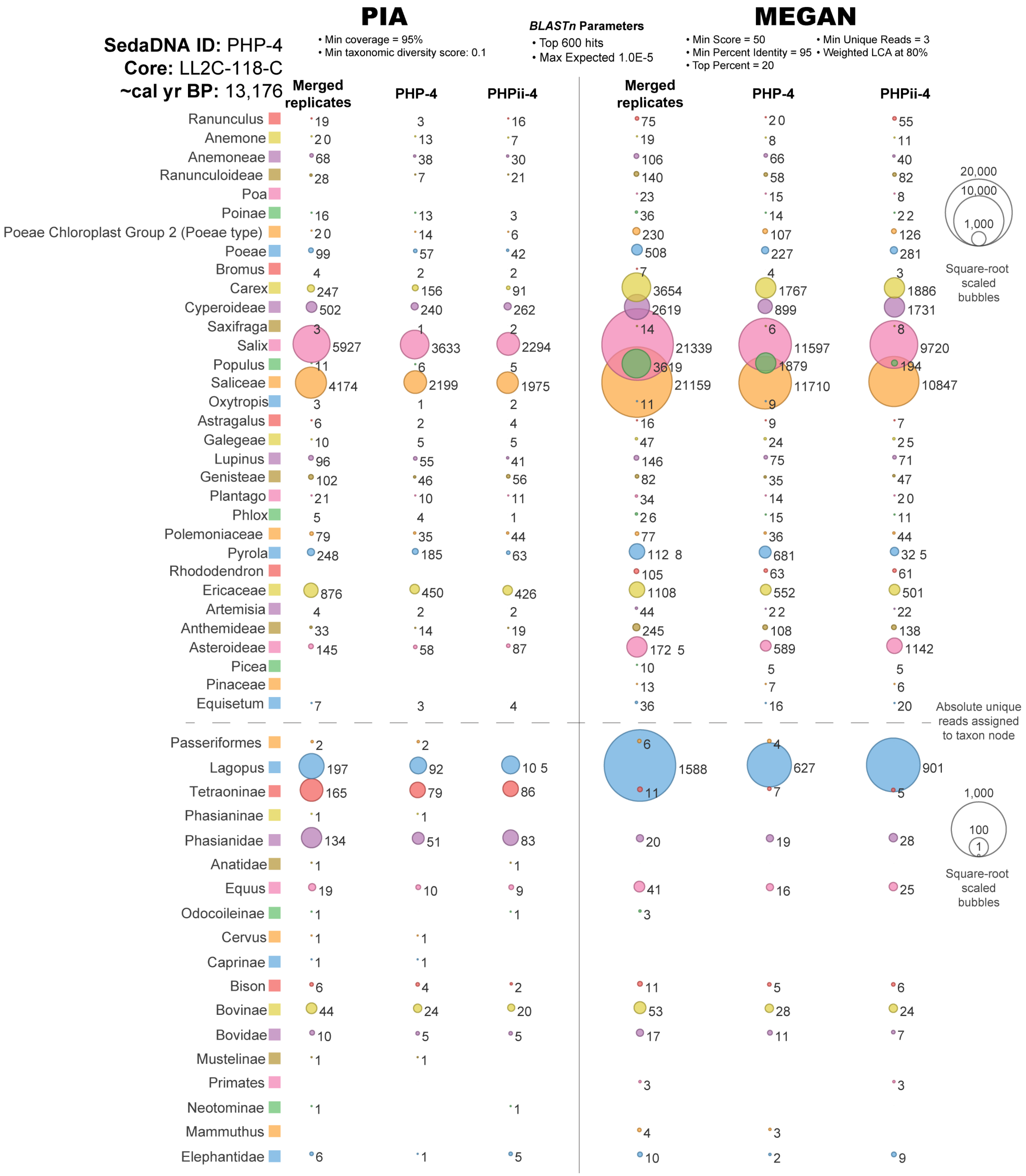

Supplementary Figure 37 PHP-4 replicates comparison with *PIA* and *MEGAN* taxonomic binning. Select nodes depicted.

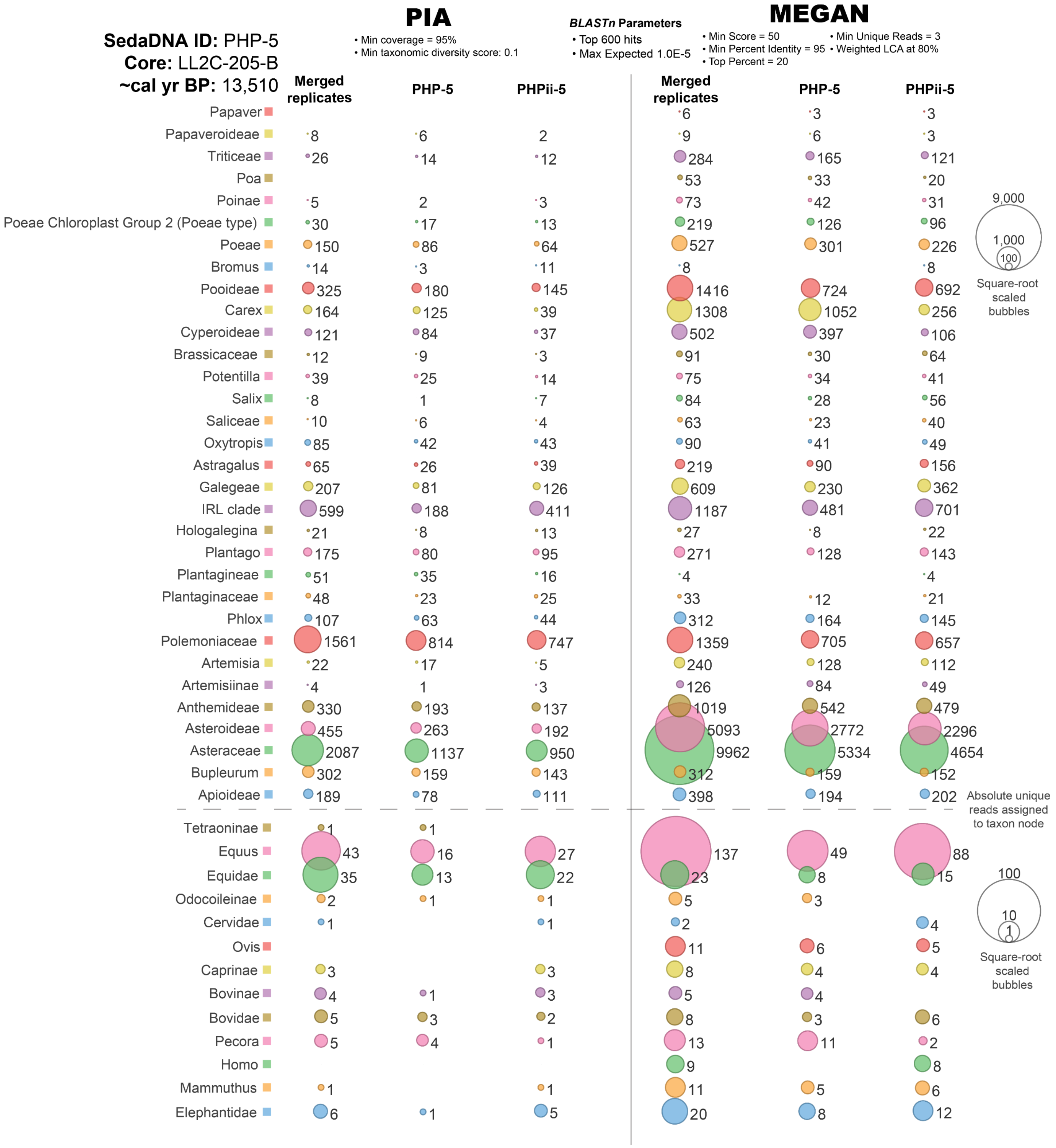

Supplementary Figure 38 PHP-5 replicates comparison with *PIA* and *MEGAN* taxonomic binning. Select nodes depicted.

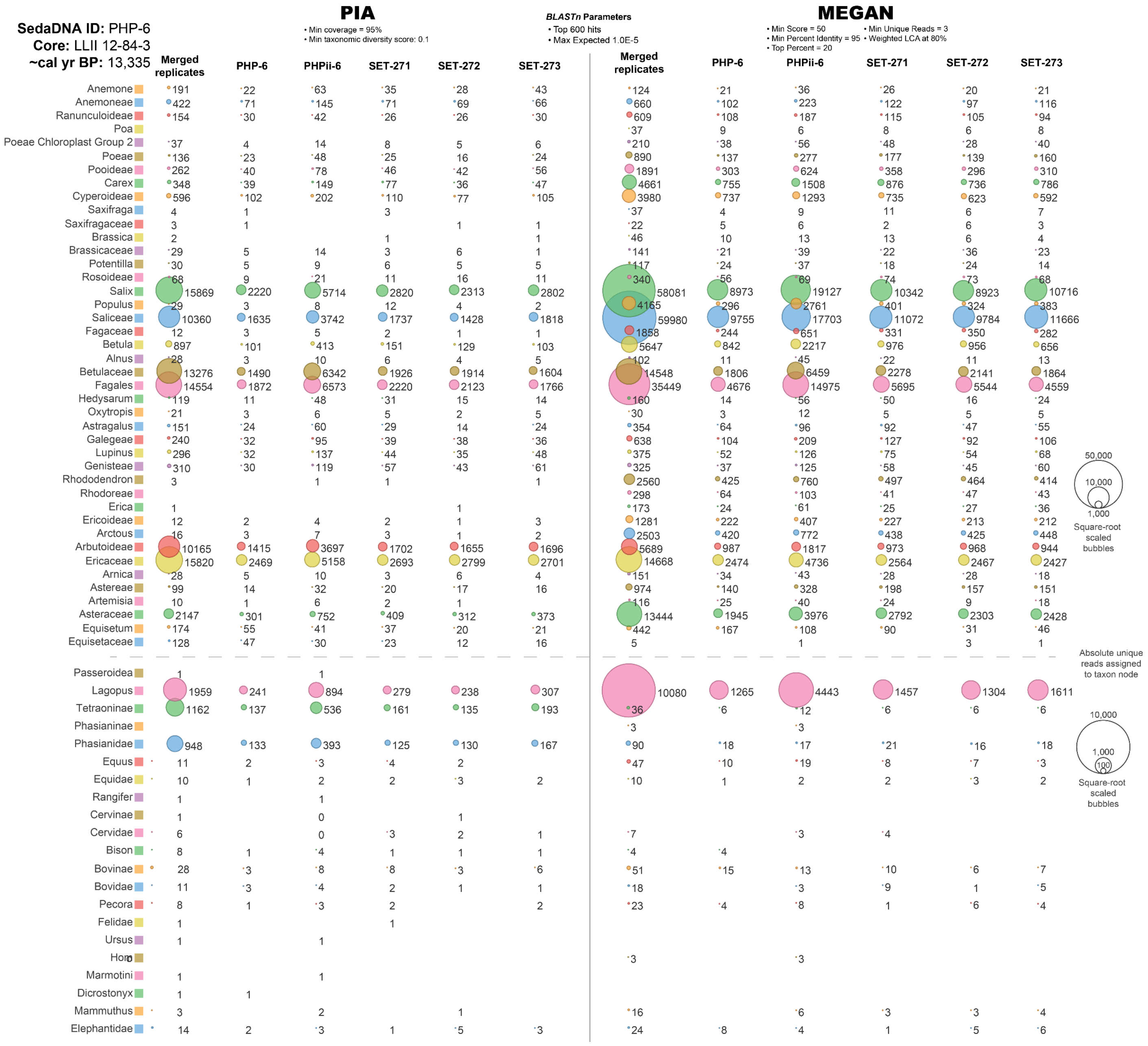

Supplementary Figure 39 PHP-6 replicates comparison with *PIA* and *MEGAN* taxonomic binning. Select nodes depicted.

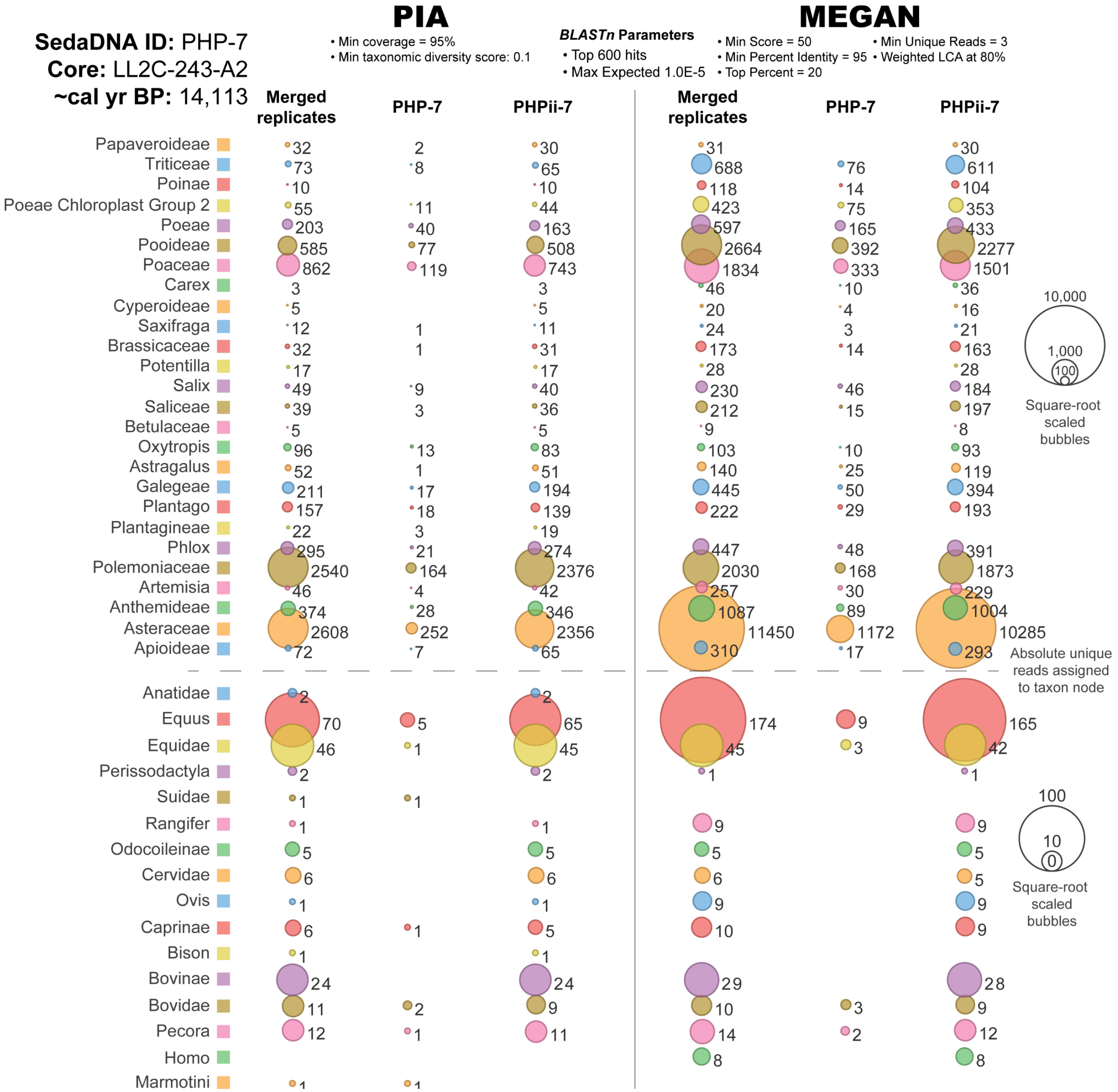

Supplementary Figure 40 PHP-7 replicates comparison with *PIA* and *MEGAN* taxonomic binning.

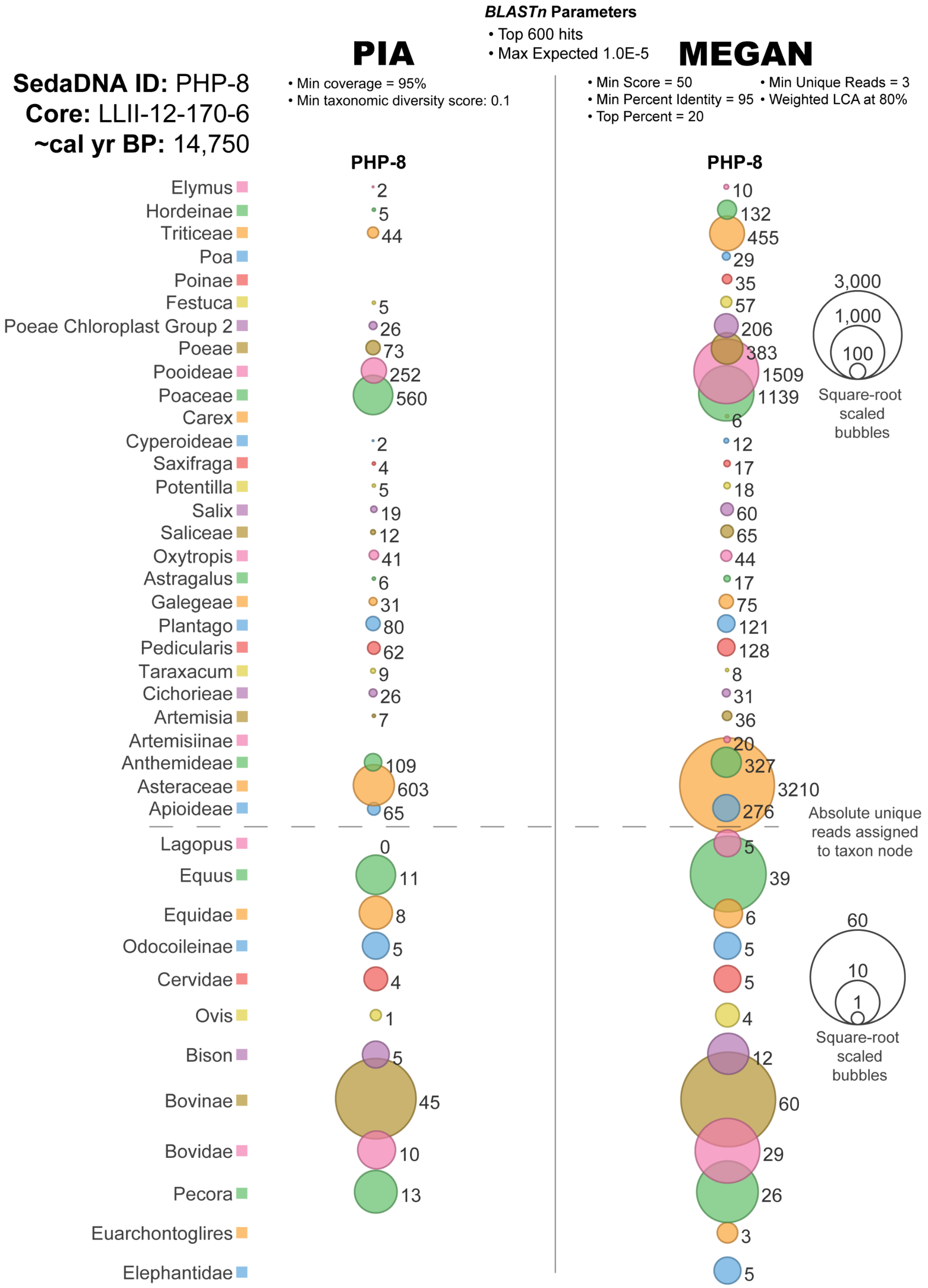

Supplementary Figure 41 PHP-8 replicates comparison with *PIA* and *MEGAN* taxonomic binning. Select nodes depicted.

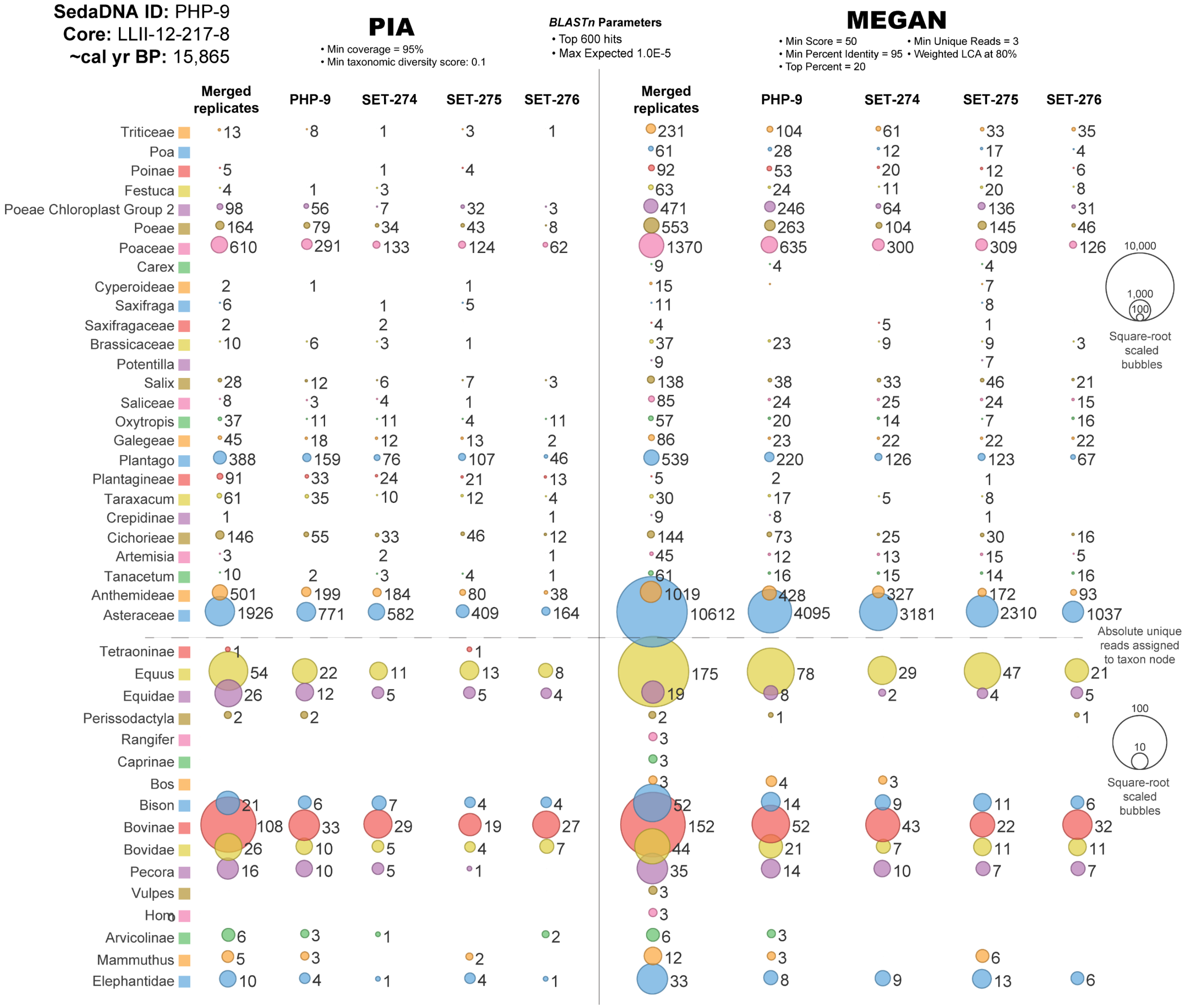

Supplementary Figure 42 PHP-9 replicates comparison with *PIA* and *MEGAN* taxonomic binning. Select nodes depicted.

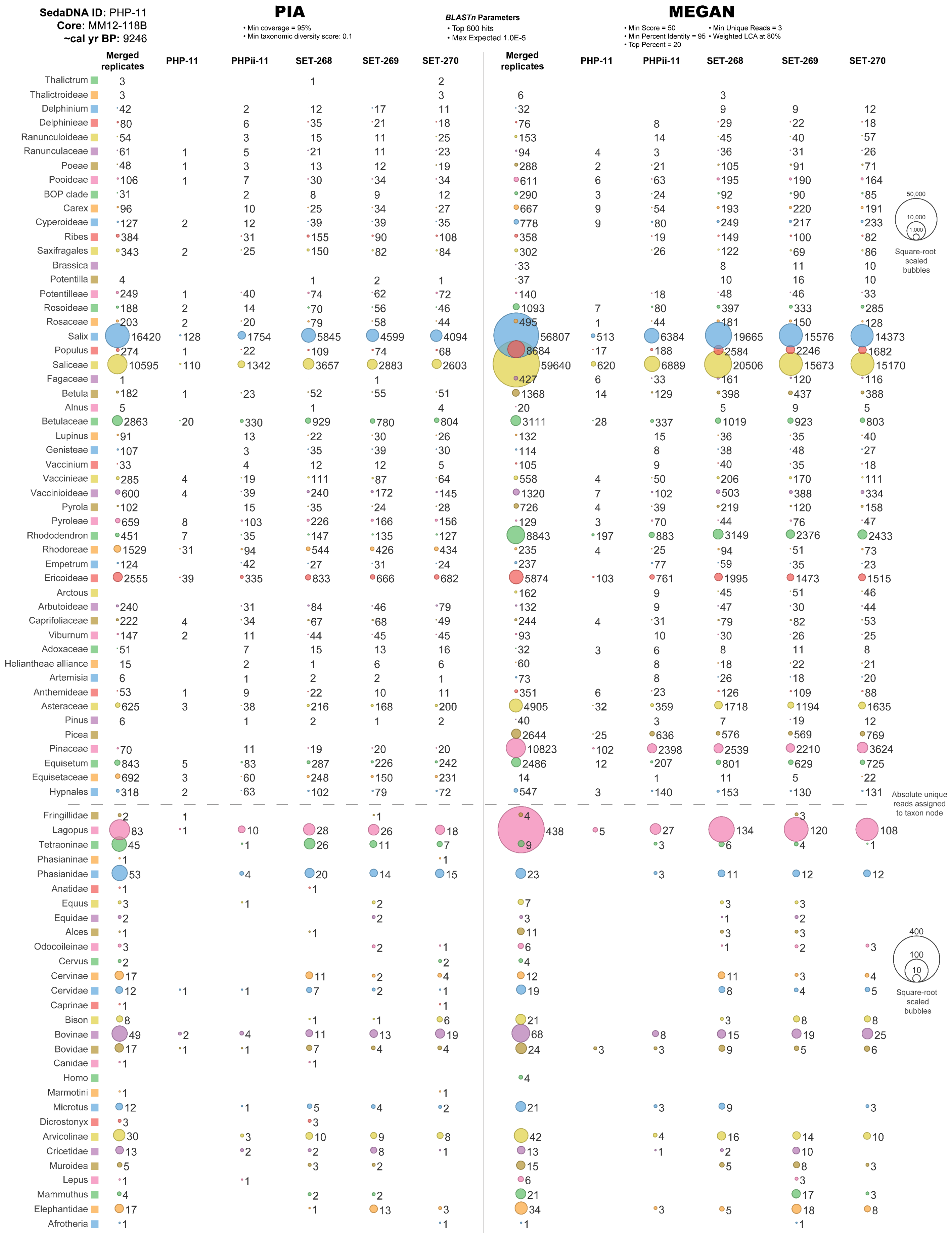

Supplementary Figure 43 PHP-11 replicates comparison with *PIA* and *MEGAN* taxonomic binning. Select nodes depicted.

SedaDNA ID: PHP-12
Core: MM12-119B
~cal yr BP: 10,051

PIA
• Min coverage = 95%
• Min taxonomic diversity score: 0.1

***BLASTn* Parameters**
• Top 600 hits
• Max Expected 1.0E-5

MEGAN
• Min Score = 50
• Min Unique Reads = 3
• Min Percent Identity = 95
• Weighted LCA at 80%
• Top Percent = 20

Taxon	PIA Merged replicates	PIA PHPii-12	PIA CSMii-12a	PIA CSMii-12b	PIA CSMii-12c	PIA CSMii-12d	MEGAN Merged replicates	MEGAN PHPii-12	MEGAN CSMii-12a	MEGAN CSMii-12b	MEGAN CSMii-12c	MEGAN CSMii-12d
Ranunculeae	185		76	30	65	14	82		29	19	27	7
Anemone	130		35	72	6	17	96		27	44	9	16
Anemoneae	203		28	147	7	21	294		39	189	25	41
Ranunculoideae	185		50	64	48	23	414		114	153	92	55
Poeae	201		47	82	36	36	551		158	168	88	137
Pooideae	158		36	66	23	33	1104		282	380	172	270
Carex	315		131	58	45	81	3028		1182	525	475	846
Cyperoideae	533		218	82	79	154	2833		1058	547	451	777
Saxifraga	41		7	6	6	22	118		32	20	21	45
Ribes	3422	1	825	465	1240	891	3095		817	429	1074	775
Chamaenerion	63		16	19	17	11	20		8	0	5	7
Gossypium	11		3	2	4	2	255		90	54	54	57
Gossypioides							241		80	61	47	53
Brassica	4		3	1			88		26	18	20	24
Draba	50		4	18	26	2	38		3	16	19	0
Potentilla	21		7	4	4	6	101		35	19	14	33
Dasiphora	6		3	2	1		70		22	13	17	18
Salix	77277	3	26002	19629	13796	17847	241882	30	79930	61099	45168	55655
Populus	8189	1	3027	1798	1588	1775	53307		17178	13340	10234	12555
Fagaceae	22		12	4	3	3	2362		752	593	484	533
Betula	1735	1	596	421	342	375	6180		1495	2008	1653	1024
Astragalus	89		19	55	7	8	166		35	97	18	16
Galegeae	152		32	94	13	13	318		78	176	25	39
Lupinus	2921		2501	127	56	237	3012		2549	134	56	273
Genisteae	2821		2451	121	51	198	2153		1787	114	54	198
Acacia	2		2				138		43	30	34	31
Pedicularis	85		47	13	8	17	162		81	26	18	37
Thymus	1				1	0	560		183	125	123	129
Vaccinium	97		26	18	38	15	196		67	29	63	37
Vaccinieae	683		181	94	276	132	1186		380	184	383	239
Vaccinioideae	1268		427	163	452	226	2849		923	423	993	510
Pyrola	1230		354	271	185	420	4094		1012	899	697	1486
Pyroleae	4438	1	1163	1009	741	1524	497		179	119	88	111
Rhododendron	73		5	3	16	49	1953		368	327	544	714
Rhodoreae	190		22	17	58	93	106		26	19	36	25
Erica	1				1		40		12	12	10	6
Empetrum	84		15	21	24	24	173		57	31	48	37
Empetreae	520		146	100	127	147	89		32	9	19	29
Arctous	11		6	1	3	1	274		88	29	83	74
Arbutoideae	1608		500	412	364	332	842		266	229	178	169
Valeriana	3		1		1	1	27		6	4	10	7
Caprifoliaceae	394		69	153	98	74	398		77	158	87	76
Viburnum	1609		566	443	171	429	1060		387	269	120	284
Adoxaceae	696	1	252	176	69	198	259		88	69	33	69
Artemisia	7		2	1		4	74		28	13	14	19
Artemisiinae	1					1	45		21	5	6	13
Anthemideae	75		27	17	18	13	415		150	88	87	90
Picea							15		0	7	3	5
Pinaceae							53		12	10	6	25
Gymnocarpium	112		48	16	32	16	586		245	68	178	95
Equisetum	10754	2	3786	2260	1667	3039	30983	6	10992	6659	4807	8519
Equisetaceae	9982		3518	2103	1590	2771	62		9	18	20	15
Hypnales	307		92	87	72	56	680		201	175	164	140

Taxon	PIA Merged replicates	PIA PHPii-12	PIA CSMii-12a	PIA CSMii-12b	PIA CSMii-12c	PIA CSMii-12d	MEGAN Merged replicates	MEGAN PHPii-12	MEGAN CSMii-12a	MEGAN CSMii-12b	MEGAN CSMii-12c	MEGAN CSMii-12d
Passeriformes	8		1	2	1	4	25		6		5	14
Lagopus	152		58	16	34	44	728		229	96	132	271
Equus	4		2	1		1	17		14		3	
Alces	41		3	13	12	13	334		36	141	58	99
Odocoileinae	70		5	36	10	19	23		6	7	5	5
Cervus	59		13	21	19	6	25		6	9	6	4
Cervinae	110		27	48	23	12	135		30	53	32	20
Cervidae	154		23	59	30	42	126		24	50	30	22
Caprinae	3		1		1	1	3		3			
Bos							3					3
Bison	25		3	10	6	6	25		4	10	5	6
Bovinae	130		16	47	24	43	147		25	46	29	47
Bovidae	24		6	9	0	9	46		10	17	5	14
Pecora	157		33	54	36	34	169		39	58	36	36
Martes	3		1		2		3		3			
Lemuriformes							3		3			
Marmotini	1					1						
Rhizomys	1					1						
Neotominae	1		1									
Myodes	2		1			1						
Microtus	72		15	12	18	27	147		37	24	34	52
Eothenomys	1			1					0	0	0	0
Dicrostonyx	5		1		3	1	15		0	0	9	6
Arvicolinae	160		45	45	22	48	142		40	44	20	38
Ctenodactylidae	1			1								
Lepus	52		31	15	4	2	112		67	25	11	9
Mammuthus							18		15	3		
Elephantidae	23		16	3	2	2	42		30	4	4	4

Bubble legend (plants): 200,000; 100,000; 10,000 — Square-root scaled bubbles

Absolute unique reads assigned to taxon node

Bubble legend (animals): 700; 100; 10 — Square-root scaled bubbles

Supplementary Figure 44 PHP-12 replicates comparison with *PIA* and *MEGAN* taxonomic binning. Select nodes depicted.

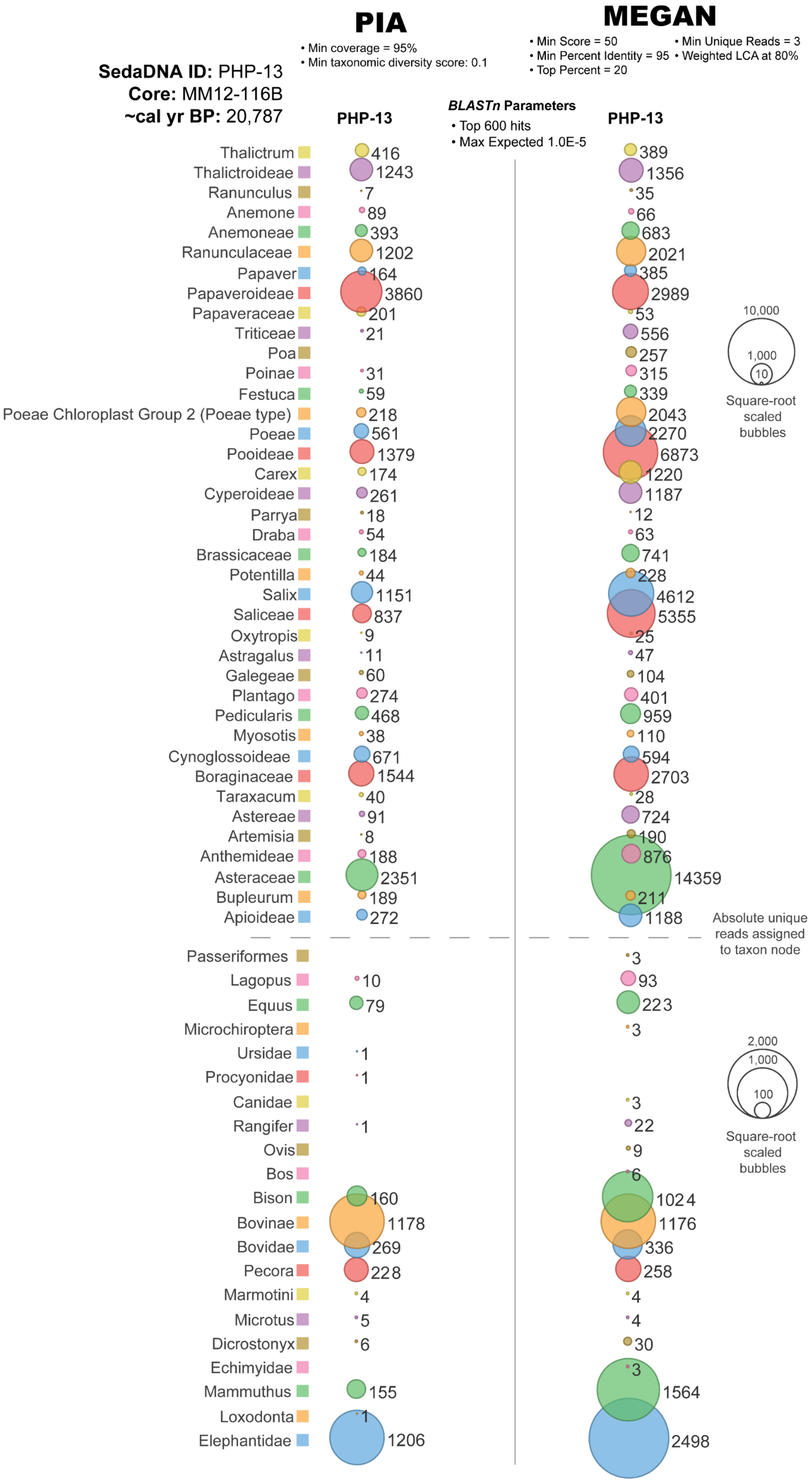

Supplementary Figure 45
PHP-13 replicates comparison with *PIA* and *MEGAN* taxonomic binning. Select nodes depicted.

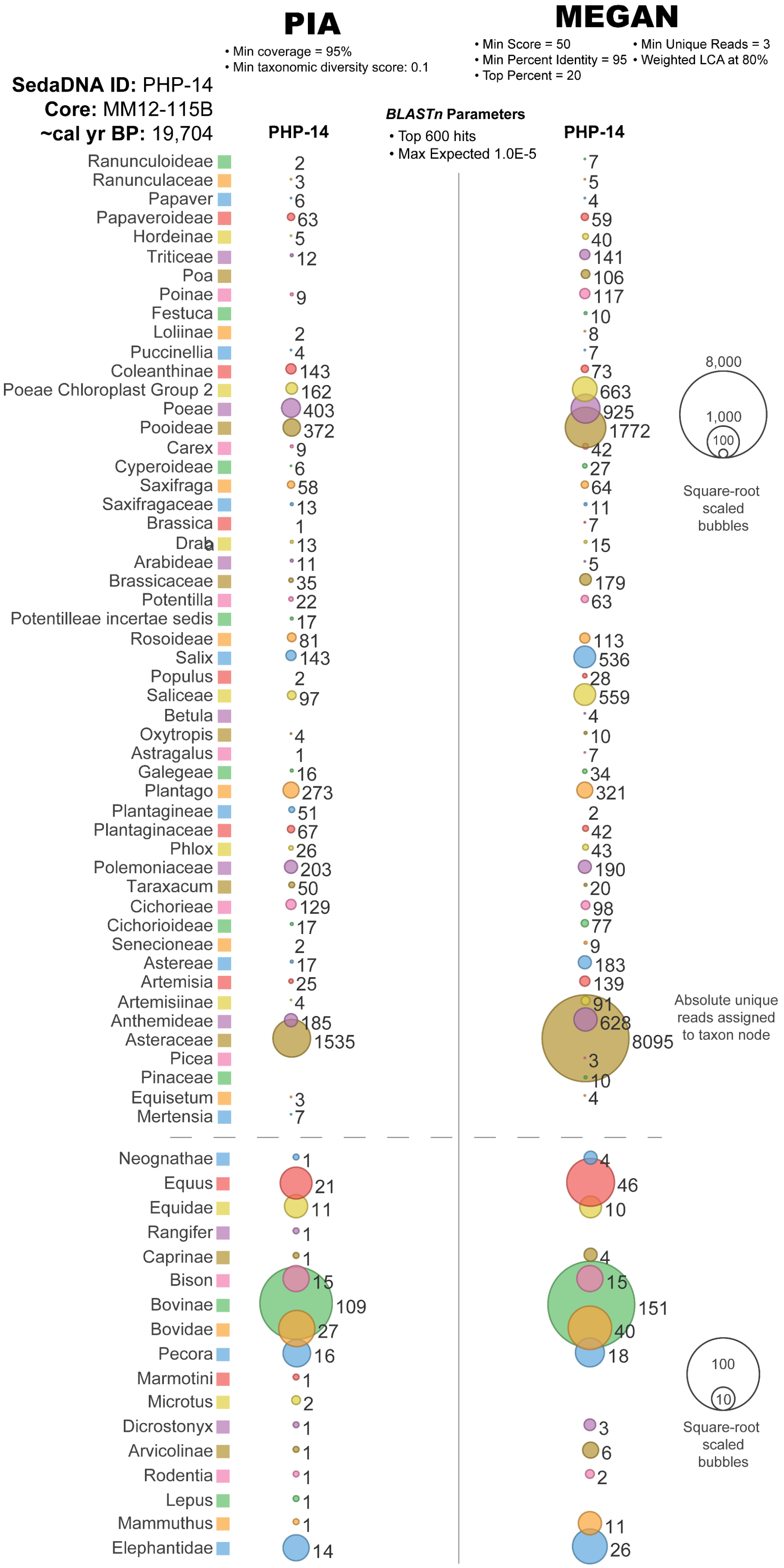

Supplementary Figure 46
PHP-14 replicates comparison with *PIA* and *MEGAN* taxonomic binning. Select nodes depicted.

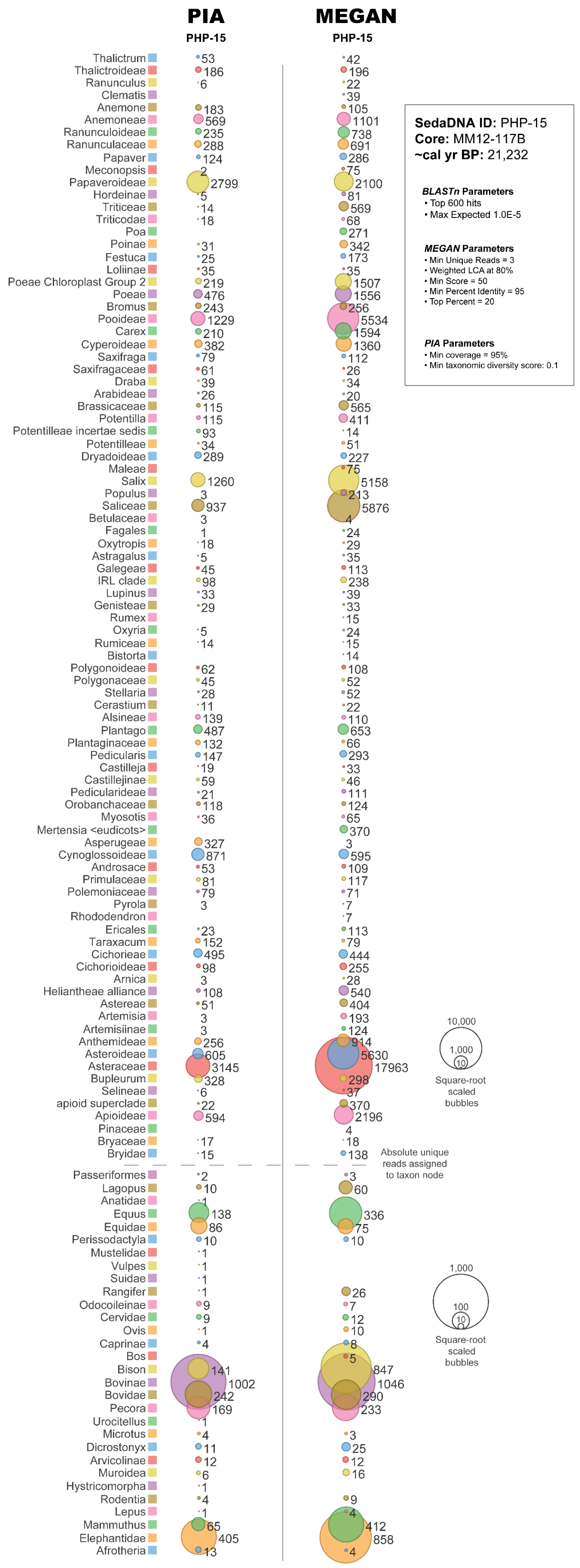

Supplementary Figure 47 PHP-15 replicates comparison with *PIA* and *MEGAN* taxonomic binning. Select nodes depicted.

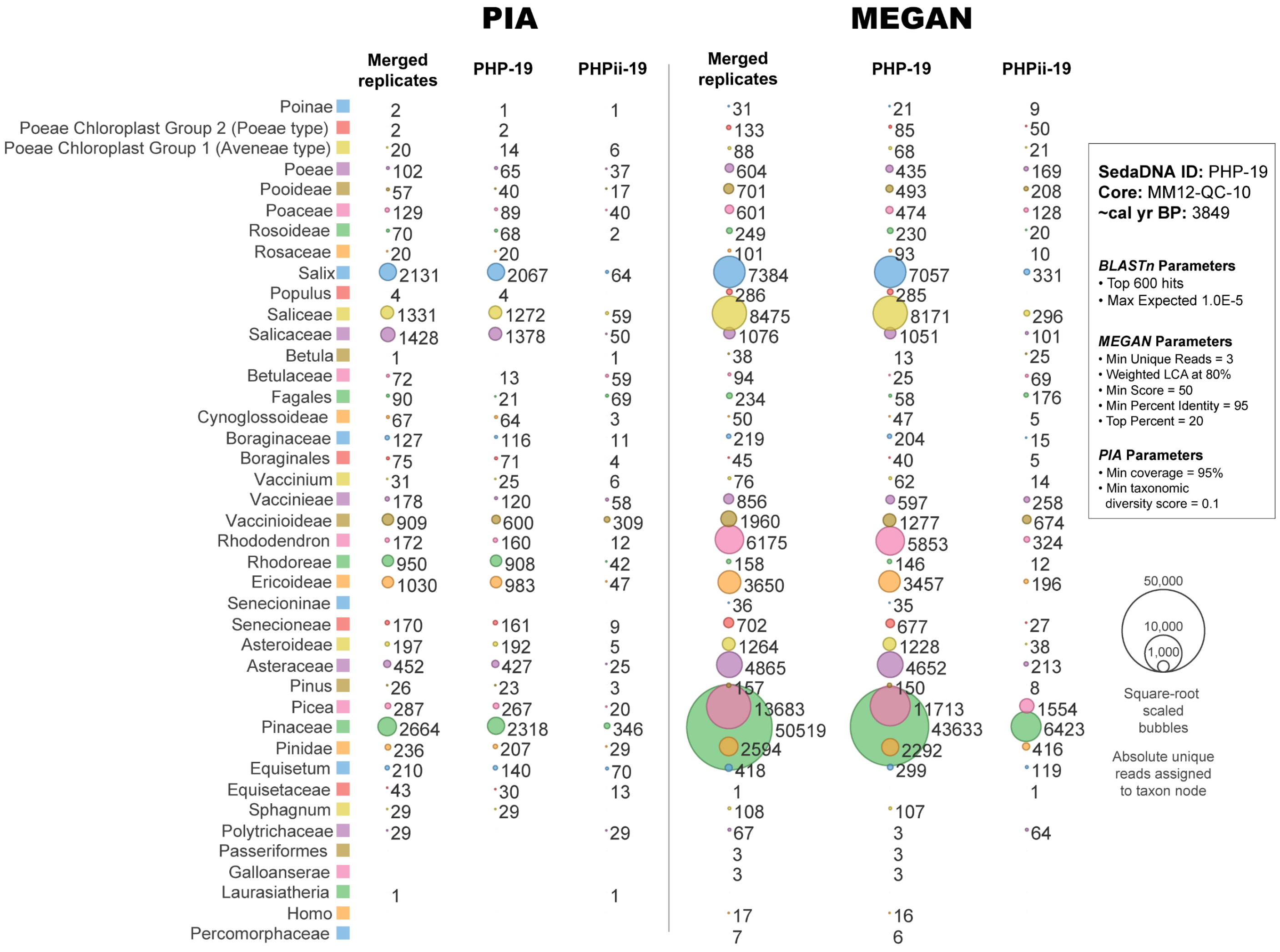

Supplementary Figure 48 PHP-19 replicates comparison with *PIA* and *MEGAN* taxonomic binning. Select nodes depicted.

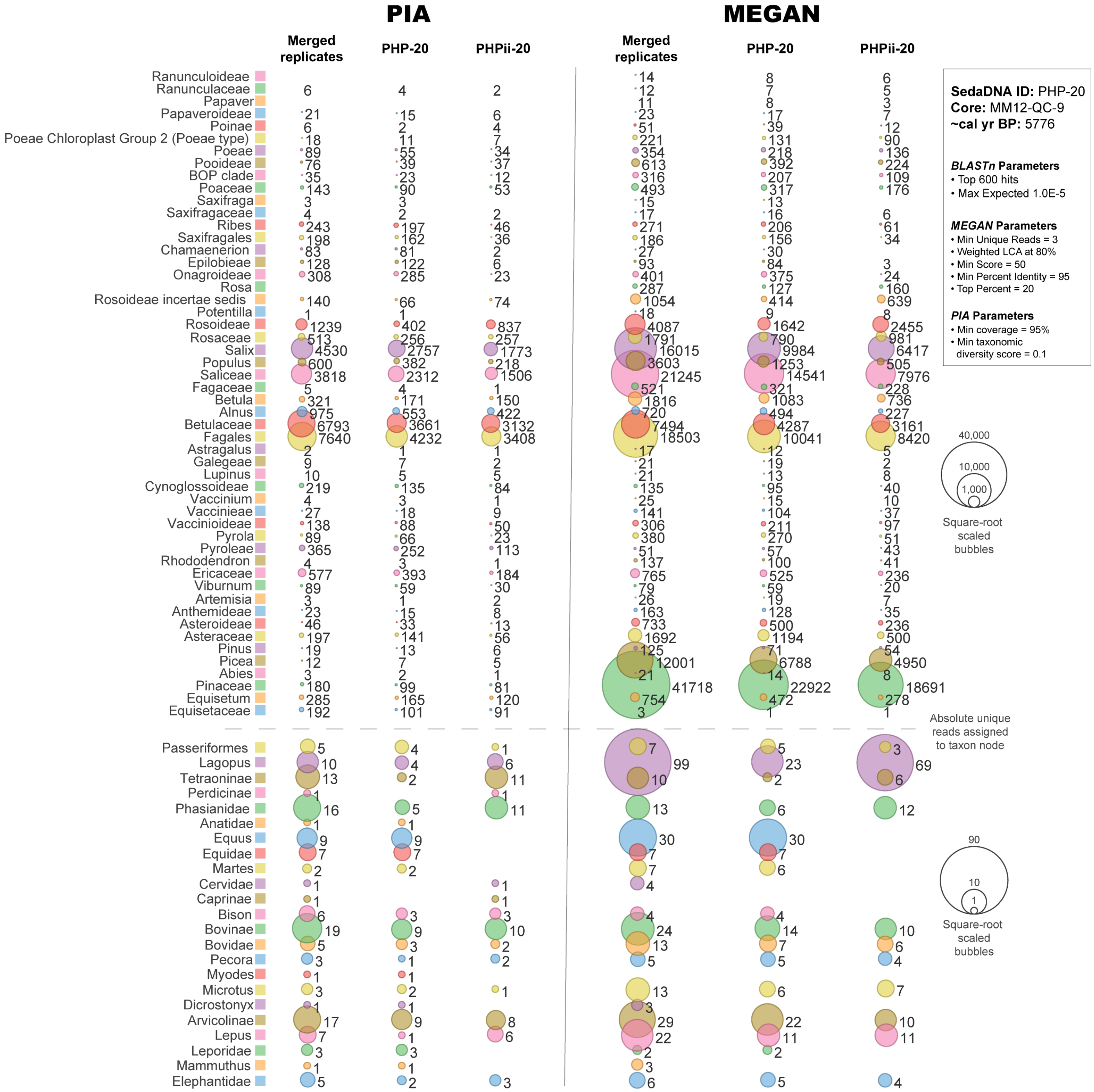

Supplementary Figure 49 PHP-20 replicates comparison with *PIA* and *MEGAN* taxonomic binning. Select nodes depicted.

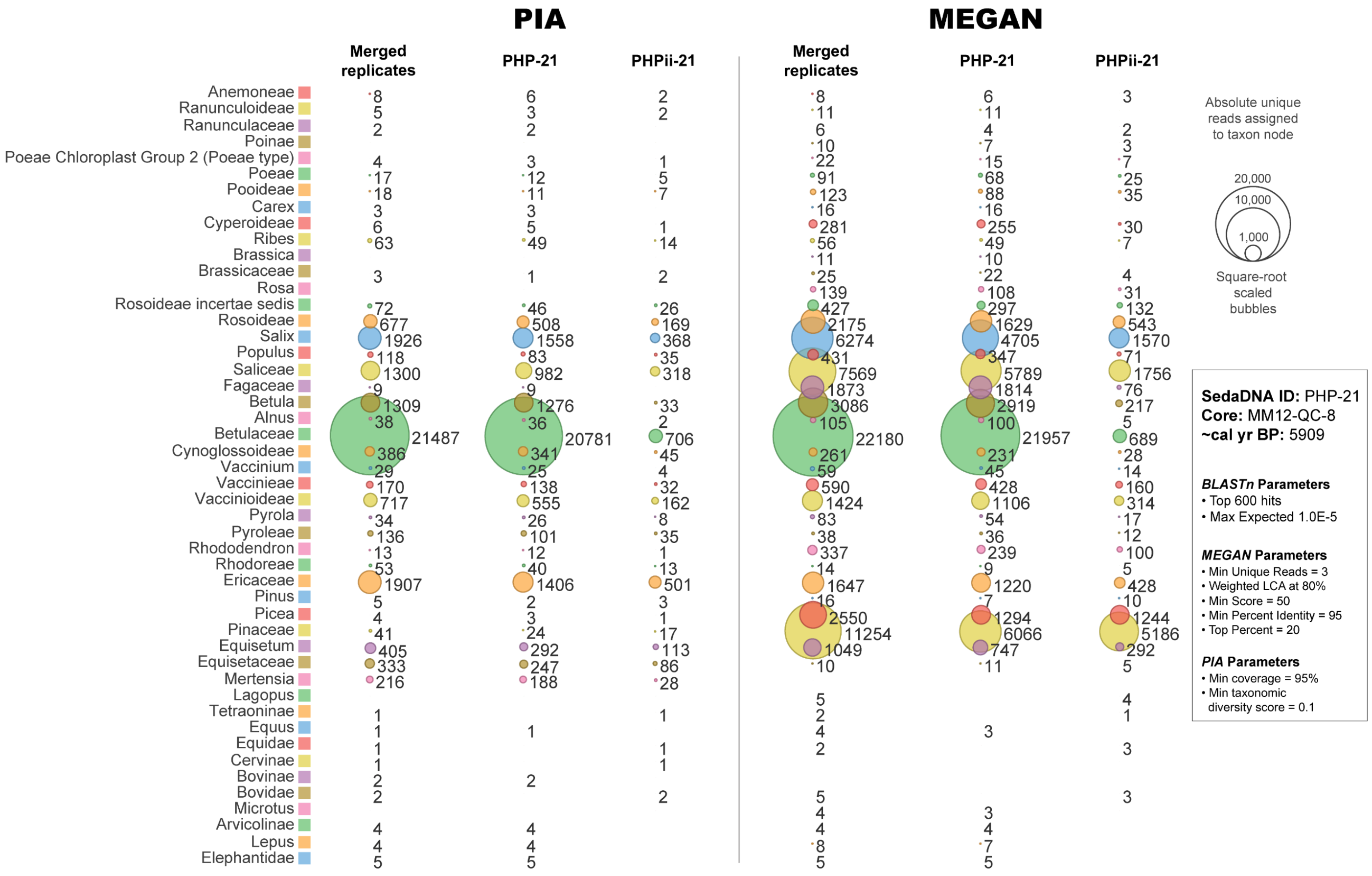

Supplementary Figure 50 PHP-21 replicates comparison with *PIA* and *MEGAN* taxonomic binning. Select nodes depicted.

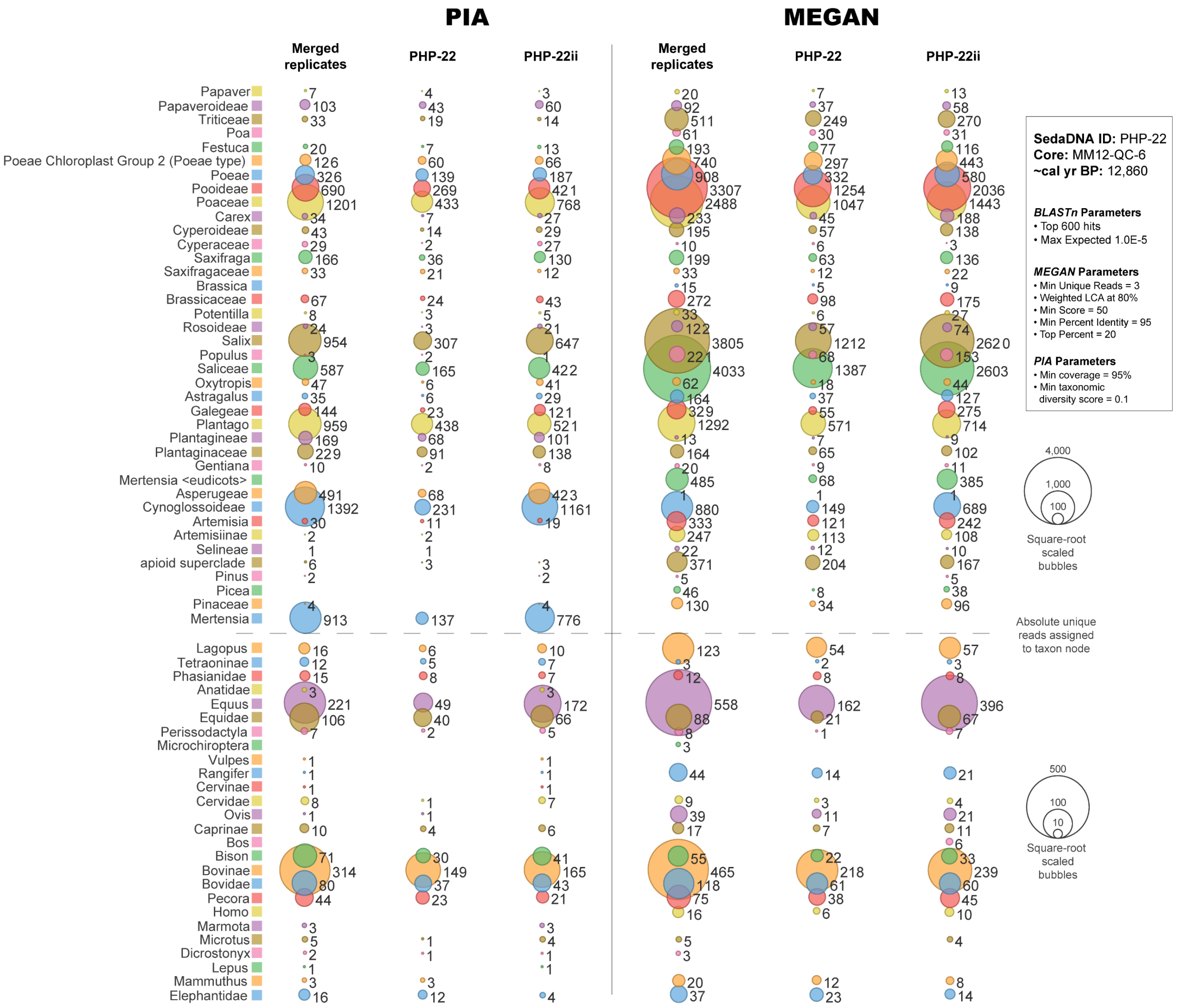

Supplementary Figure 51 PHP-22 replicates comparison with *PIA* and *MEGAN* taxonomic binning. Select nodes depicted.

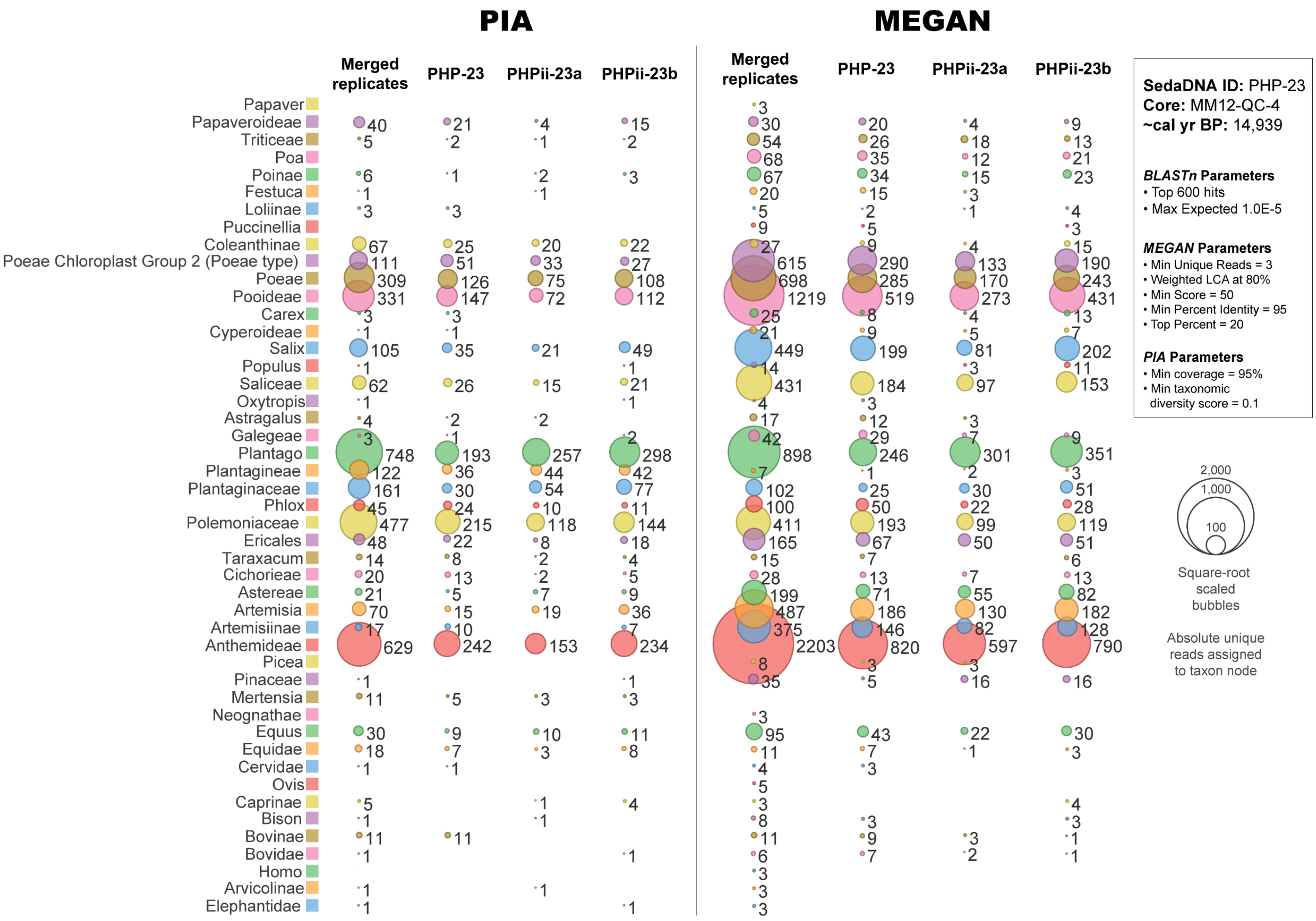

Supplementary Figure 52 PHP-23 replicates comparison with *PIA* and *MEGAN* taxonomic binning. Select nodes depicted.

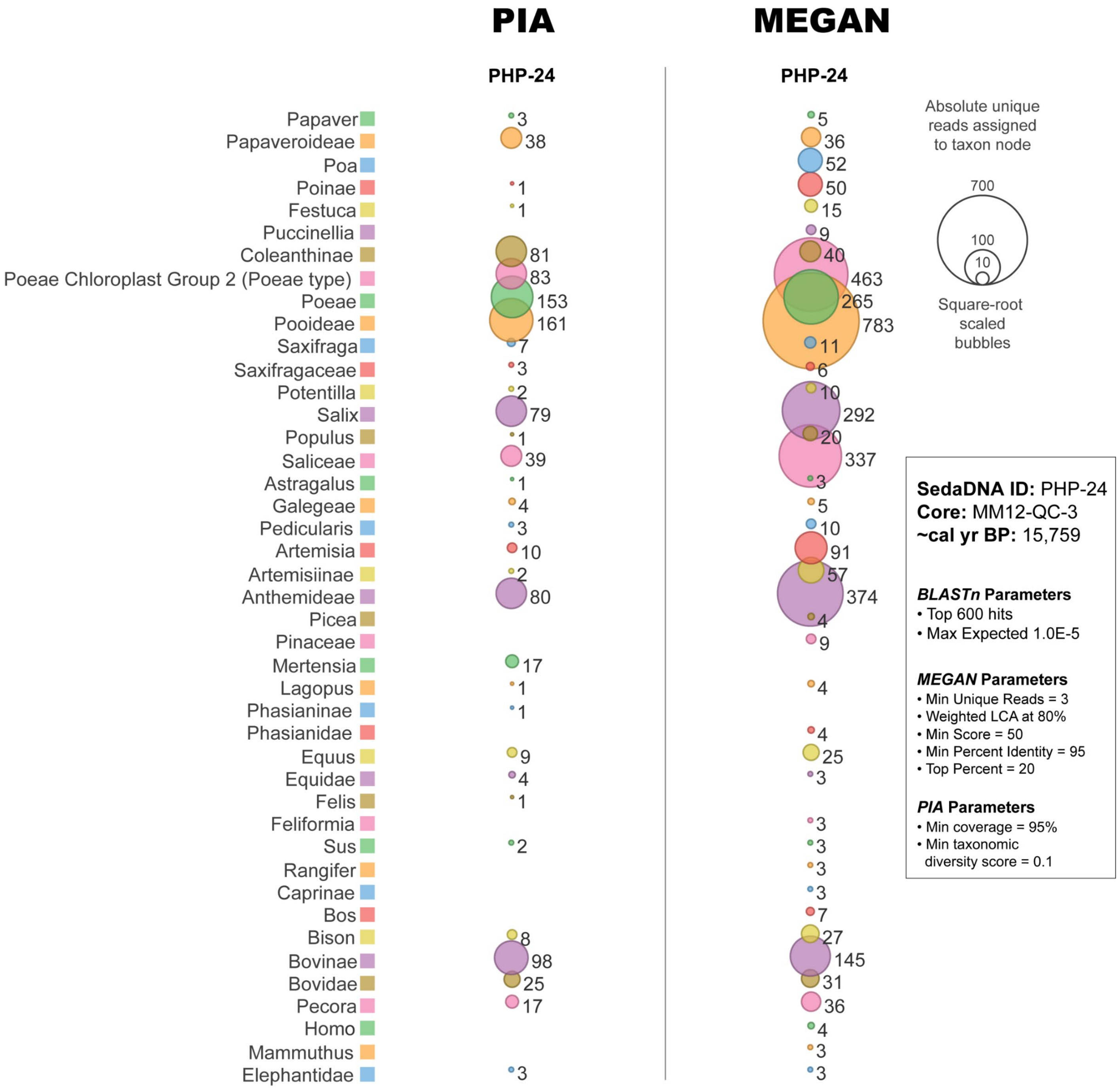

Supplementary Figure 53 PHP-24 replicates comparison with *PIA* and *MEGAN* taxonomic binning. Select nodes depicted.

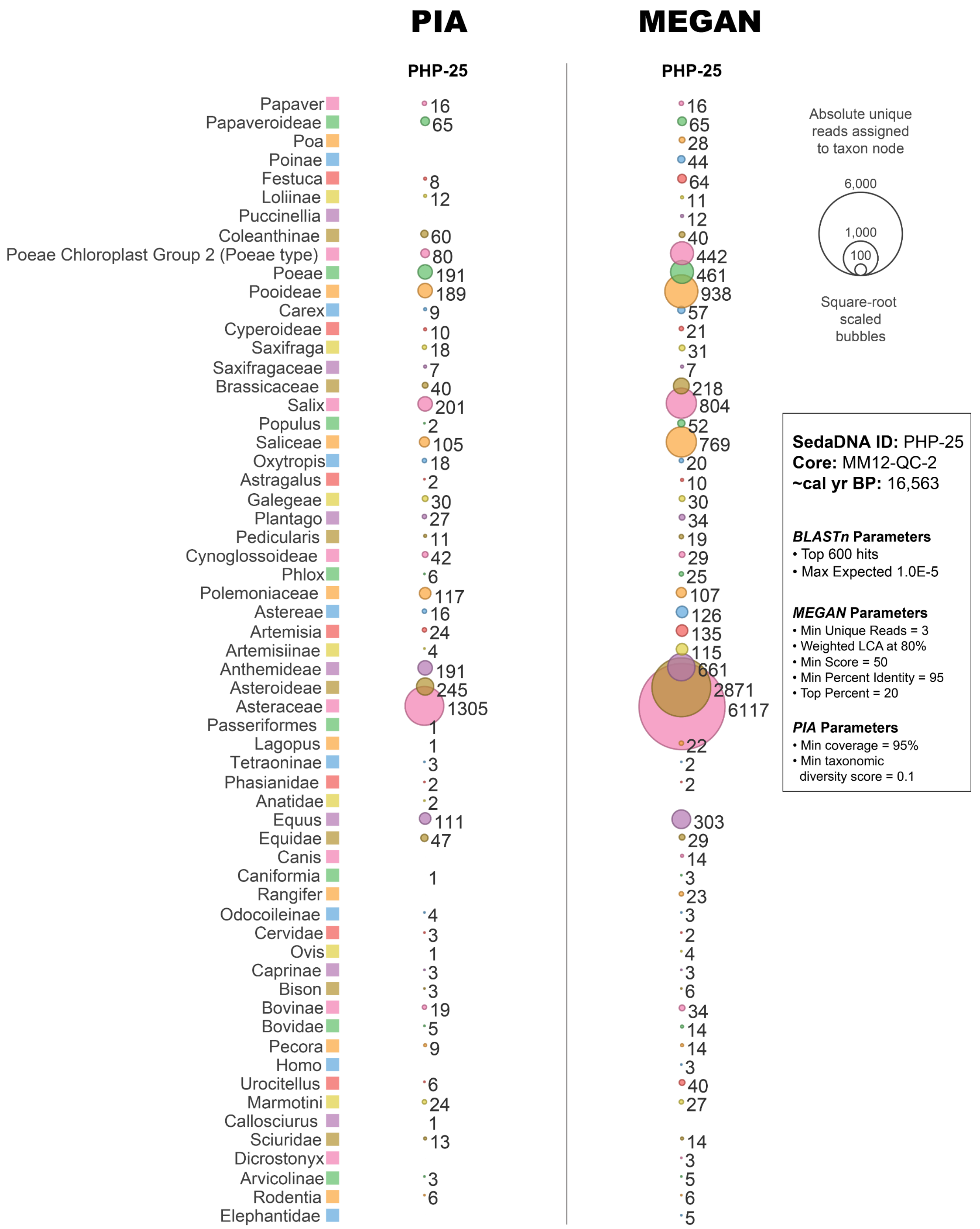

Supplementary Figure 54 PHP-24 replicates comparison with *PIA* and *MEGAN* taxonomic binning.

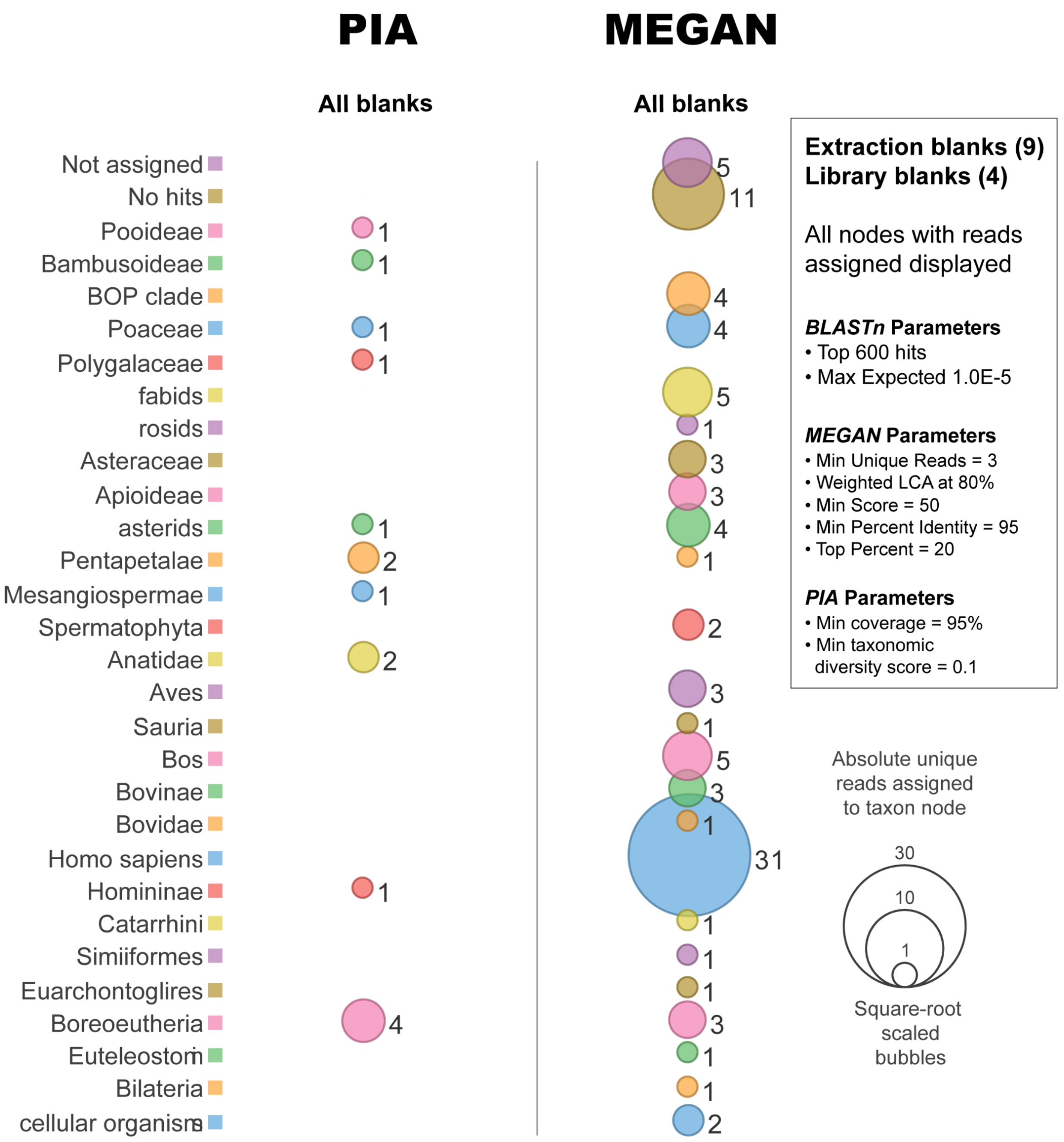

Supplementary Figure 55 Blanks comparison with *PIA* and *MEGAN* taxonomic binning.

2.5.7 GC content and ghost range fragment lengths

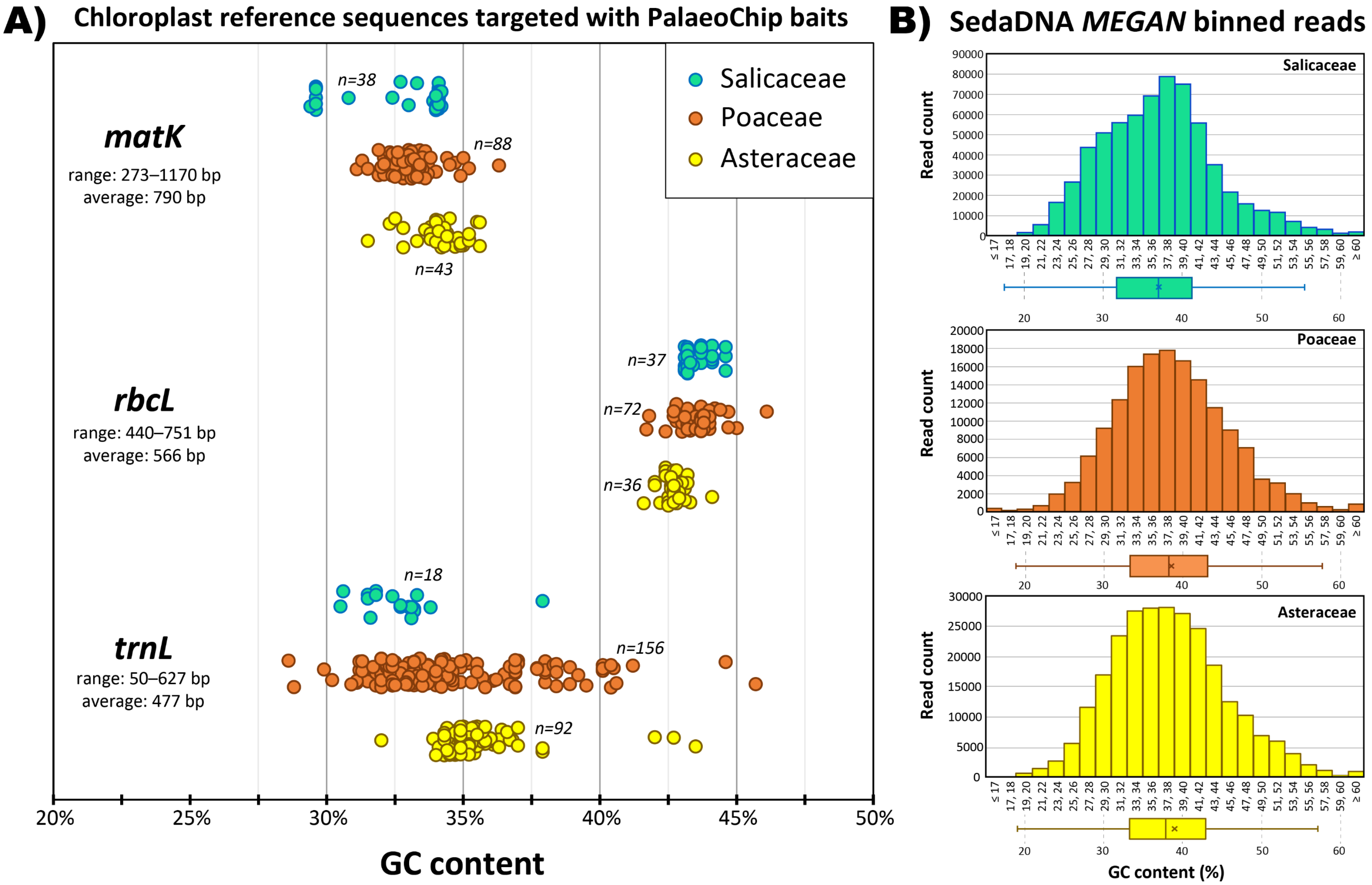

Supplementary Figure 56 Assessing GC content of reference sequences and binned reads.

A) GC content of reference sequences (limited to Salicaceae, Poaceae, and Asteraceae) of the PalaeoChip Arctic v1.0 bait-set targeting loci within the chloroplast genome. Dots indicate GC content of the reference sequence.

B) GC content of sedaDNA reads map-filtered, *BLASTn* assigned, and *MEGAN* binned to the three target loci. Includes molecules enriched from all three chloroplast loci (*matK*, *rbcL*, *trnL*). SedaDNA reads tend to have a GC content mode of ~37% irrespective of family, which may imply some degree of preferential bias. However, reads were sequenced from ~20–80% GC from the three loci, and could be assigned to all three families.

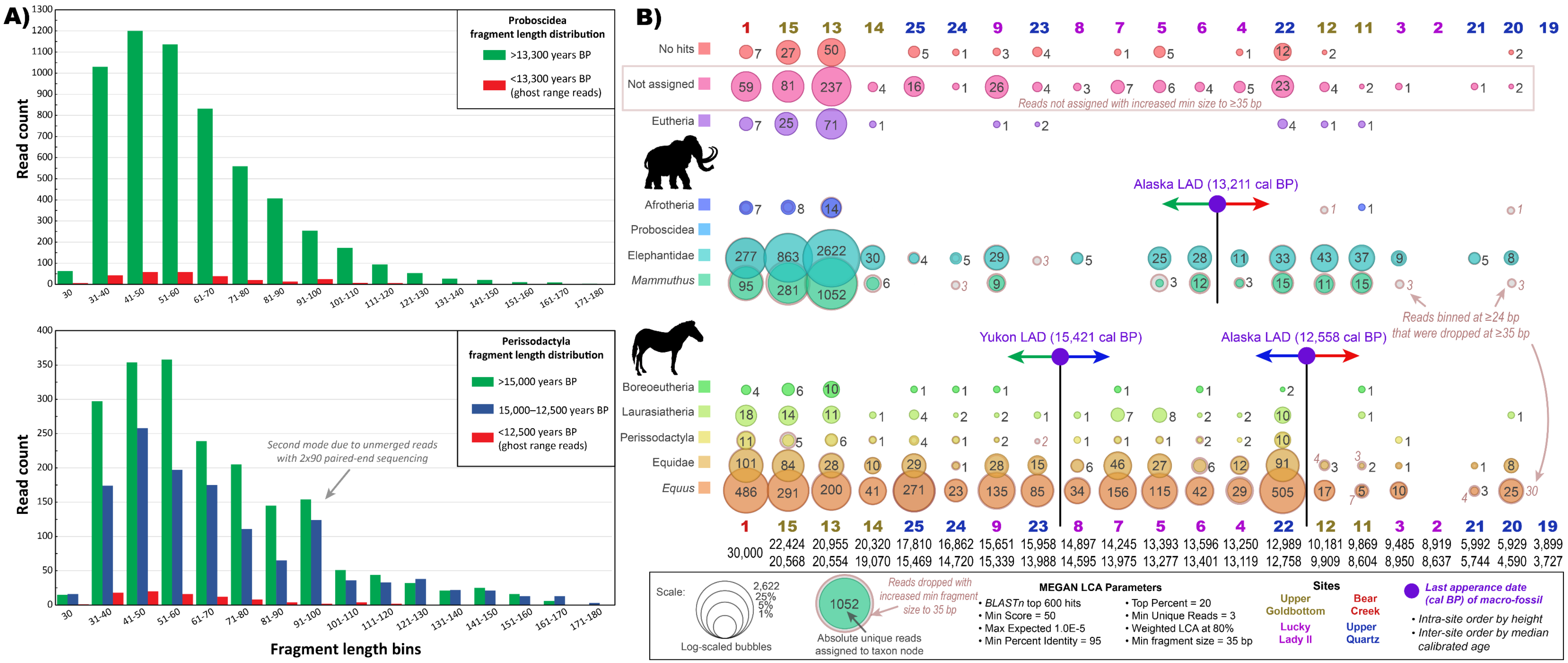

Supplementary Figure 57 Ghost range fragment length comparison (absolute unique read counts).

A) Fragment length distributions of reads binned within Proboscidea and Perissodactyla subdivided between periods that pre and post-date the last known appearance of that taxon with macrofossils in eastern Beringia. Most reads have fragment lengths between 30–50 BP, even among the ghost range samples.

B) Reads re-processed in *MEGAN* that were assigned to nodes within Proboscidea and Perissodactyla with an increased minimum fragment length to 35 bp. These plots demonstrate that exceptionally short fragments (~24 bp) have not influenced the taxonomic binning of ghost range reads. Some reads are dropped (not assigned) relative to the main-text bubble chart with the increased minimum fragment length (Fig. 2), but the signal does not disappear.

3. Supplementary References

1. Stuart, A. J. Late Quaternary megafaunal extinctions on the continents: a short review. *Geol. J.* **50**, 414–433 (2015).
2. Pitulko, V. V. *et al.* Early human presence in the Arctic: Evidence from 45,000-year-old mammoth remains. *Science (80-.).* **351**, 260–263 (2016).
3. Maschenko, E. N. *et al.* The Zhenya Mammoth (Mammuthus primigenius (Blum.)): Taphonomy, geology, age, morphology and ancient DNA of a 48,000 year old frozen mummy from western Taimyr, Russia. *Quat. Int.* **445**, 104–134 (2017).
4. Graf, K. E. Siberian Odyssey. in *Paleoamerican Odyssey* (eds. Graf, K. E., Ketron, C. V. & Waters, M. R.) 65–80 (Texas A&M University Press, 2014).
5. Pitulko, V. V. *et al.* The Yana RHS site: humans in the arctic before the last glacial maximum. *Science (80-.).* **303**, 52–56 (2004).
6. Hoffecker, J. F. & Elias, S. A. *Human Ecology of Beringia*. (Columbia University Press, 2007).
7. Nikolskiy, P. & Pitulko, V. Evidence from the Yana Palaeolithic site, Arctic Siberia, yields clues to the riddle of mammoth hunting. *J. Archaeol. Sci.* **40**, 4189–4197 (2013).
8. Clark, P. U. The Last Glacial Maximum. *Science (80-.).* **325**, 710–714 (2009).
9. Bourgeon, L. Bluefish Cave II (Yukon Territory , Canada): Taphonomic Study of a Bone Assemblage. *PaleoAmerica* **1**, 105–108 (2015).
10. Bourgeon, L., Burke, A. & Higham, T. Earliest human presence in North America dated to the last glacial maximum: New radiocarbon dates from Bluefish Caves, Canada. *PLoS One* **12**, e0169486 (2017).
11. Vachula, R. S. *et al.* Sedimentary biomarkers reaffirm human impacts on northern Beringian ecosystems during the Last Glacial period. *Boreas* **49**, 514–525 (2020).
12. Holmes, C. E. Tanana River Valley archaeology circa 14,000 to 9000 BP. *Arctic Anthropol.* **38**, 154–170 (2001).
13. Holmes, C. E. The Beringian and Transitional Periods in Alaska. in *From the Yenisei to the Yukon: Interpreting Lithic Assemblage Variability in Late Pleistocene/Early Holocene Beringia* (eds. Goebel, T. E. & Buvit, I.) 179–191 (Texas A&M University Press, 2011).
14. Potter, B. A. *et al.* Current evidence allows multiple models for the peopling of the Americas. *Sci. Adv.* **4**, 1–9 (2018).
15. Abramova, Z. A. Paleolit Severnoi Azii. in *Paleolit Kavkaza i Severnoi Azii* (ed. Boriskovskii, P. I.) 145–243 (Nauka, 1989).
16. Abramova, Z. A., Astakhov, S. N., Vasil’ev, S. A., Ermolva, N. M. & Lisitsyn, N. F. *Paleolit Eniseya*. (Nauka, 1991).
17. Goebel, T. Siberian middle Upper Paleolithic. in *Encyclopedia of prehistory. Vol 2: Arctic and Subarctic* (eds. Peregrine, P. N. & Ember, M.) 192–196 (Kluwer Academic Publishers, 2002).
18. Ermolova, N. M. *Teriofauna doliny Angary v pozdem antropogene*. (Nauka, 1978).
19. Goebel, T. Pleistocene Human Colonization of Siberia and Pepoling of the Americas: An

Ecological Approach. *Evol. Anthropol.* **8**, 208–227 (1999).
20. Davis, R. S. & Ranov, V. A. Recent Work on the Paleolithic of Central Asia. *Evol. Anthropol.* **8**, 186–193 (1999).
21. Dolukhanov, P. M., Shukurov, A. M., Tarasov, P. E. & Zaitseva, G. I. Colonization of Northern Eurasia by modern humans: Radiocarbon chronology and environment. *J. Archaeol. Sci.* **29**, 593–606 (2002).
22. Graf, K. E. Adandomment of the Siberian Mammoth-Steppe during the LGM: Evidence from the calibration of 14C-dated archaeological occupations. *Curr. Res. Pleistocene* **22**, 2–5 (2005).
23. Graf, K. E. 'The Good, the Bad, and the Ugly': evaluating the radiocarbon chronology of the middle and late Upper Paleolithic in the Enisei River valley, south-central Siberia. *J. Archaeol. Sci.* **36**, 694–707 (2009).
24. Graf, K. E. Hunter-gatherer disperals in the Mammoth-Steppe: Technological provisioning and land-use in the Enisei River Valley, south-central Siberia. *J. Archaeol. Sci.* **37**, 210–23 (2010).
25. Fiedel, S. J. & Kuzmin, Y. V. Radiocarbon date frequency as an index of intensity of Paleolithic occupation of Siberia: Did humans react predictably to climate oscillations? *Radiocarbon* **49**, 741–756 (2007).
26. Kuzmin, Y. V. & Keates, S. G. Dates are not just data: Paleolithic settlement patterns in Siberia derived from radiocarbon records. *Am. Antiq.* **70**, 773–789 (2005).
27. Kuzmin, Y. V. Siberia at the Last Glacial Maximum: Environment and archaeology. *J. Archaeol. Res.* **16**, 163–221 (2008).
28. Izuho, M. Human Technological and Behavioral Adaptation to Landscape Changes around the Last Glacial Maximum in Japan: A Focus on Hokkaido. in *Paleoamerican Odyssey* (eds. Graf, K. E., Ketron, C. V. & Waters, M. R.) 45–64 (Texas A&M University Press, 2014).
29. Buvit, I. & Terry, K. Outside Beringia: Why the Northeast Asian Upper Paleolithic Record Does Not Support a Long Standstill Model. *PaleoAmerica* **5563**, 1–5 (2016).
30. Goebel, T. The 'microblade adaptation' and recolonization of Siberia during the late Upper Pleistocene. in *Thinking small: Global perspectives on microlithization. Archaeological Paper No. 12* (eds. Elston, R. G. & Kuhn, S. L.) 117–131 (American Anthropological Association, 2002).
31. Bronk Ramsey, C. Deposition models for chronological records. *Quat. Sci. Rev.* **27**, 42–60 (2008).
32. Reimer, P. J. *et al.* The IntCal20 Northern Hemisphere Radiocarbon Age Calibration Curve (0-55 cal kBP). *Radiocarbon* **62**, 725–757 (2020).
33. D'Costa, V. M. *et al.* Antibiotic resistance is ancient. *Nature* **477**, 457–61 (2011).
34. Froese, D. G., Zazula, G. D. & Reyes, A. V. Seasonality of the late Pleistocene Dawson tephra and exceptional preservation of a buried riparian surface in central Yukon Territory, Canada. *Quat. Sci. Rev.* **25**, 1542–1551 (2006).
35. Mahony, M. E. 50,000 years of paleoenvironmental change recorded in meteoric waters

and coeval paleoecological and cryostratigraphic indicators from the Klondike goldfields, Yukon, Canada. (University of Alberta, 2015). doi:10.7939/R34T6FF58

36. Sadoway, T. R. A Metagenomic Analysis of Ancient Sedimentary DNA Across the Pleistocene-Holocene Transition. (McMaster University, 2014).

37. Fægri, K., Kaland, P. E. & Krzywinski, K. *Textbook of pollen analysis*. *Textbook of pollen analysis.* (John Wiley & Sons Ltd., 1989).

38. McAndrews, J. H., Berti, A. A. & Norris, G. *Key to the quaternary pollen and spores of the Great Lakes region*. (Royal Ontario Museum, 1973).

39. Hansen, B. C. Conifer stomate analysis as a paleoecological tool: An example from the Hudson Bay Lowlands. *Can. J. Bot.* **73**, 244–252 (1995).

40. van Geel, B. & Aptroot, A. Fossil ascomycetes in Quaternary deposits. *Nov. Hedwigia* 313–329 (2006). doi:10.1127/0029-5035/2006/0082-0313

41. van Geel, B. Non-pollen palynomorphs. in *Tracking environmental change using lake sediments* 99–119 (Springer, 2001).

42. Haas, J. N. Neorhabdocoela oocytes-Palaeoecological indicators found in pollen preparations from Holocene freshwater lake sediments. *Rev. Palaeobot. Palynol.* **91**, 371–382 (1996).

43. Turton, C. L. & McAndrews, J. H. Rotifer loricas in second millennium sediment of Crawford Lake, Ontario, Canada. *Rev. Palaeobot. Palynol.* **141**, 1–6 (2006).

44. McCarthy, F. M. C., Pilkington, P. M., Volik, O., Heyde, A. & Cocker, S. L. Non-pollen Palynomorphs in Freshwater Sediments and their Palaeolimnological Potential and Selected Applications. *Geol. Soc. London, Spec. Publ.* **511**, SP511-2020–109 (2020).

45. *Non-pollen palynomorphs as relevant indicators in palaeoecology and archaeobotany*. *Special Issue: Review of Palaeobotany and Palynology* **186**, (2012).

46. Miola, A. Tools for Non-Pollen Palynomorphs (NPPs) analysis: A list of Quaternary NPP types and reference literature in English language (1972-2011). *Rev. Palaeobot. Palynol.* 142–161 (2012). doi:10.1016/j.revpalbo.2012.06.010

47. Langeveld, B. W. *et al.* A multidisciplinary study of a Late Pleistocene arctic ground squirrel (Urocitellus parryii) midden from Yukon, Canada. *Quat. Res.* **89**, 333–351 (2018).

48. Murchie, T. J. *et al.* Optimizing extraction and targeted capture of ancient environmental DNA for reconstructing past environments using the PalaeoChip Arctic-1.0 bait-set. *Quat. Res.* **99**, 305–328 (2021).

49. Karpinski, E., Mead, J. I. & Poinar, H. N. Molecular identification of paleofeces from Bechan Cave, southeastern Utah, USA. *Quat. Int.* **443**, 140–146 (2016).

50. Dabney, J. *et al.* Complete mitochondrial genome sequence of a Middle Pleistocene cave bear reconstructed from ultrashort DNA fragments. *Proc. Natl. Acad. Sci.* **110**, 15758–15763 (2013).

51. Enk, J. *et al.* Mammuthus population dynamics in Late Pleistocene North America: Divergence, Phylogeogrpaphy and Introgression. *Front. Ecol. Evol.* **4**, 1–13 (2016).

52. Meyer, M. & Kircher, M. Illumina sequencing library preparation for highly multiplexed

target capture and sequencing. *Cold Spring Harb. Protoc.* **5**, (2010).

53. Jónsson, H., Ginolhac, A., Schubert, M., Johnson, P. L. F. & Orlando, L. MapDamage2.0: Fast approximate Bayesian estimates of ancient DNA damage parameters. *Bioinformatics* **29**, 1682–1684 (2013).

54. Percy, D. M. *et al.* Understanding the spectacular failure of DNA barcoding in willows (Salix): Does this result from a trans-specific selective sweep? *Mol. Ecol.* 4737–4756 (2014). doi:10.1111/mec.12837

55. Hübler, R. *et al.* HOPS: automated detection and authentication of pathogen DNA in archaeological remains. *Genome Biol.* **20**, (2019).

56. Dabney, J., Meyer, M. & Pääbo, S. Ancient DNA damage. *Cold Spring Harb. Perspect. Biol.* **5**, 1–7 (2013).

57. Deagle, B. E., Eveson, J. P. & Jarman, S. N. Quantification of damage in DNA recovered from highly degraded samples - A case study on DNA in faeces. *Front. Zool.* **3**, 11 (2006).

58. Kistler, L., Ware, R., Smith, O., Collins, M. & Allaby, R. G. A new model for ancient DNA decay based on paleogenomic meta-analysis. *Nucleic Acids Res.* **45**, 6310–6320 (2017).

59. Lisiecki, L. E. & Raymo, M. E. A Pliocene-Pleistocene stack of 57 globally distributed benthic δ 18O records. *Paleoceanography* **20**, 1–17 (2005).

60. Cribdon, B., Ware, R., Smith, O., Gaffney, V. & Allaby, R. G. PIA: More Accurate Taxonomic Assignment of Metagenomic Data Demonstrated on sedaDNA From the North Sea. *Front. Ecol. Evol.* **8**, 1–12 (2020).

61. Conroy, K. J. *et al.* Tracking late-Quaternary extinctions in interior Alaska using megaherbivore bone remains and dung fungal spores. *Quat. Res.* 1–12 (2020). doi:10.1017/qua.2020.19

62. Grootes, P. M. & Stuiver, M. Oxygen 18/16 variability in Greenland snow and ice with 10-3- to 105-year time resolution. *J. Geophys. Res. Ocean.* **102**, 26455–26470 (1997).
