# Supplementary material for: Collapse of the mammoth-steppe in central Yukon as revealed by ancient environmental DNA
Source: Nat Commun. 2021 Dec 8;12:7120. doi: 10.1038/s41467-021-27439-6 (PMC8654998; doi:10.1038/s41467-021-27439-6)
Supplement: Supplementary file 2 — Reporting Summary [file 41467_2021_27439_MOESM2_ESM.pdf]

nature research

Corresponding author(s): Tyler Murchie, Duane Froese, Hendrik Poinar

Last updated by author(s): Nov 6, 2021

Reporting Summary

Nature Research wishes to improve the reproducibility of the work that we publish. This form provides structure for consistency and transparency in reporting. For further information on Nature Research policies, see our Editorial Policies and the Editorial Policy Checklist.

Statistics

For all statistical analyses, confirm that the following items are present in the figure legend, table legend, main text, or Methods section.

n/a	Confirmed	
☐	☒	The exact sample size (n) for each experimental group/condition, given as a discrete number and unit of measurement
☒	☐	A statement on whether measurements were taken from distinct samples or whether the same sample was measured repeatedly
☐	☒	The statistical test(s) used AND whether they are one- or two-sided<br>*Only common tests should be described solely by name; describe more complex techniques in the Methods section.*
☒	☐	A description of all covariates tested
☒	☐	A description of any assumptions or corrections, such as tests of normality and adjustment for multiple comparisons
☐	☒	A full description of the statistical parameters including central tendency (e.g. means) or other basic estimates (e.g. regression coefficient) AND variation (e.g. standard deviation) or associated estimates of uncertainty (e.g. confidence intervals)
☐	☒	For null hypothesis testing, the test statistic (e.g. F, t, r) with confidence intervals, effect sizes, degrees of freedom and P value noted<br>*Give P values as exact values whenever suitable.*
☒	☐	For Bayesian analysis, information on the choice of priors and Markov chain Monte Carlo settings
☒	☐	For hierarchical and complex designs, identification of the appropriate level for tests and full reporting of outcomes
☒	☐	Estimates of effect sizes (e.g. Cohen's d, Pearson's r), indicating how they were calculated

Our web collection on statistics for biologists contains articles on many of the points above.

Software and code

Policy information about availability of computer code

Data collection	No software used during data collection.
Data analysis	bcl2fastq (v 1.8.4); BCL2BAM2FASTQ (fastq2bam); leeHom v1.1.5; network-aware BWA v0.5.10; samtools v0.1.18; libbam-retrieveMapped_single_and_ProperlyPair; biohazard-bam-rmdup v0.5.14; NGSeXplore v0.1.5; Geneious v6.1.5; mapDamage 2.0 v2.0.3; BLAST v2.9.0; MEGAN v6.19.7; PIA v5.4; Adobe Illustrator v25.4.1; NGSXRemoveDuplicates v0.1.5; Oxcal v4.4.2. Kistler et al. (2017) scripts available at https://doi.org/10.5061/dryad.5r10j

For manuscripts utilizing custom algorithms or software that are central to the research but not yet described in published literature, software must be made available to editors and reviewers. We strongly encourage code deposition in a community repository (e.g. GitHub). See the Nature Research guidelines for submitting code & software for further information.

Data

Policy information about availability of data

All manuscripts must include a data availability statement. This statement should provide the following information, where applicable:

- Accession codes, unique identifiers, or web links for publicly available datasets
- A list of figures that have associated raw data
- A description of any restrictions on data availability

Reference sequences from NCBI GenBank were used to map-filter the sequence data to the targets we enriched for using the PalaeoChip Arctic v1.0 bait-set. The reference and bait sequences are available in a Zenodo repository (https://doi.org/10.5281/zenodo.5643845). Sequence data map-filtered to the PalaeoChip bait reference sequences are publicly available on the NCBI SRA under BioProject PRJNA722670, Accessions: SRR14265632– SRR14265692. Source data on metagenomic assignment can be found in the source data file, and other source data are included in the supplementary information.

Field-specific reporting

Please select the one below that is the best fit for your research. If you are not sure, read the appropriate sections before making your selection.

☐ Life sciences ☐ Behavioural & social sciences ☒ Ecological, evolutionary & environmental sciences

For a reference copy of the document with all sections, see nature.com/documents/nr-reporting-summary-flat.pdf

Ecological, evolutionary & environmental sciences study design

All studies must disclose on these points even when the disclosure is negative.

Study description	Metagenomic analysis of faunal and floral organelle ancient DNA retrieved from permafrost cores in central Yukon, Canada dating between 30,000 to 4,000 years before present. Study evaluates macro-ecosystem turnover during Pleistocene-Holocene transition to understand factors that led to the mass extinction of Beringian megafauna (woolly mammoth, Yukon wild horse, and steppe bison). A gradual, long term decline of megafaunal sedimentary ancient DNA was observed, along with the coeval turnover from steppe grasslands to woody shrubs between 13,000-10,000 years ago, and the establishment of boreal taxa thereafter. A late persistence of megafaunal DNA into the Holocene was also identified, which post-dates the extirpation of these animals from Yukon and Alaska based on extant fossil records.
Research sample	Permafrost cores collected near Dawson City, Yukon were selected for sedimentary ancient DNA analysis to evaluate ecological change during the Pleistocene-Holocene transition. These cores were collected and studied by previous researchers at the University of Alberta and McMaster University (and kept in cold storage therafter). These cores were chosen for this study based on their previously identified age, coring site, and availability.
Sampling strategy	Permafrost cores were collected between June and August of 2010, 2012, and 2013 with research permits issued to DF from Yukon Heritage Branch. Sampling locations were placer gold mining exposures chosen for the quality of the exposure and expected age of sediments. Cores were drilled in permafrost exposures with a portable gas-powered drill (Echo) fitted with a 3" diameter concrete coring bit, removed from the exposure and placed in plastic core bags, labeled and immediately placed in a cooler for transport and storage in a -20C chest freezer. Cores were kept frozen in the chest freezer in the field and transported back to the University of Alberta or McMaster University for storage and subsampling. Permafrost cores were chosen for this study based on availability from previous research at the McMaster Ancient DNA Centre. Upper Goldbottom, Bear Creek, and Upper Quartz cores were re-subsampled for this study as described in the methods (cores were split in an ancient DNA clean lab and effort was made to ensure that inner core material was not contaminated by that of sediment from the core exteriors). The Lucky Lady II materials were previously subsampled in 2013 and kept in cold storage at McMaster University until they were reprocessed for this study.
Data collection	Core locations were recorded with a GPS in the field and these locations along with stratigraphic information were recorded in a field notebook at the time of sampling. Field coring was conducted by DF, ST, and MM. Field coring, sedimentology, palynology, and radiometric dating were conducted by researchers and affilates at the University of Alberta. Genetic work was conducted at the McMaster Ancient DNA Centre with sequencing (on an Illumina HiSeq 1500) at the McMaster Genome Facility within the Farncome Institute. qPCR data collected on a BioRad CFX96, post pooling quantifications were recorded on a Agilent TapeStation.
Timing and spatial scale	Core samples collected within 40 km SE of Dawson City, Yukon by Duane Froese, Matt Mahony, and Tara Sadoway (among others) between June and August in 2010, 2012, and 2013 for graduate research theses completed in 2014/2015. All cores and subsamples (from Lucky Lady II) remained untouched in cold storage at McMaster University until 2018-2020, at which time these samples were reanalyzed using new sedimentary ancient DNA techniques to expand on previous research efforts. Subsampling of Upper Goldbottom, Bear Creek, and Upper Quartz cores occurred through TJM's doctoral thesis.
Data exclusions	Only sequence data that mapped to our targeted bait-set (Holarctic flora and fauna) were analyzed for this study. Other microbial sequence data (which does not map to the PalaeoChip bait references) has been excluded here for use in parallel research.
Reproducibility	Most samples were extracted and processed with subsampled replicates in subsequent processing batches. The qPCR and metagenomic sequencing data are consistent between replicates, indicating that the metagenomic profiles reconstructed here are representative of the samples themselves and not the result of methodological bias. Replicate metagenomic comparisons between BLAST>MEGAN and PIA taxonomic binning are detailed in the SI. Negative controls were used throughout to monitor for contamination. Sequence data is publicly available to facilitate reproducibility.
Randomization	Samples were processed in parallel with a set of replicate batches as needed to improve extraction success. These data were compared and later compiled by core to reduce batch related variation. Little variation was observed in the megagenomic comparison or qPCR data. A previous study (Murchie et al. [2021], published in Quaternary Research) compared a range of processing variants to optimize wet lab procedures with these same core samples. As such, methodological comparisons were not the objective here and randomization was not part of the research design. All bioinformatic analyses were conducted simultaneously to ensure a consistency in program versions and databases. Negative controls were utilized with each batch of samples to monitor for wet-lab contamination, which were processed identically in the lab and bioinformatically.
Blinding	Core samples were assigned new lab identifiers to blind against baised treatment (PHP-1,2,3, etc.) and were processed identically in the wet lab and bioinformatically. Core identification, ages, and sites were only re-assigned to the sample identifiers when analyzing the results and ordering the samples stratigraphically. No direct comparisons were drawn between sample treatments. Blinding was not part of the research design as our primary goal was ecological reconstruction, not a methods comparison.

Did the study involve field work? ☐ Yes ☒ No

Reporting for specific materials, systems and methods

We require information from authors about some types of materials, experimental systems and methods used in many studies. Here, indicate whether each material, system or method listed is relevant to your study. If you are not sure if a list item applies to your research, read the appropriate section before selecting a response.

Materials & experimental systems

n/a	Involved in the study
☒	☐ Antibodies
☒	☐ Eukaryotic cell lines
☐	☒ Palaeontology and archaeology
☒	☐ Animals and other organisms
☒	☐ Human research participants
☒	☐ Clinical data
☒	☐ Dual use research of concern

Methods

n/a	Involved in the study
☒	☐ ChIP-seq
☒	☐ Flow cytometry
☒	☐ MRI-based neuroimaging

Palaeontology and Archaeology

Specimen provenance

Permafrost cores were collected between June and August of 2010, 2012, and 2013 with research permits issued to Duane Froese from the Yukon Heritage Branch. Sampling locations were placer gold mining exposures chosen for the quality of the exposure and expected age of the sediments. Cores were drilled in permafrost exposures with a portable gas-powered drill (Echo) fitted with a 3" diameter concrete coring bit, removed from the exposure and placed in plastic core bags. These were labeled and immediately placed in a cooler for transport and storage in a -20C chest freezer. Cores were kept frozen in the chest freezer in the field and transported back to the University of Alberta or McMaster University for storage and subsampling. Permafrost cores were chosen for this study based on availability from previous research at the McMaster Ancient DNA Centre. No new cores were collected for this study. All cores and subsamples remain cold storage either at McMaster University (PI: Hendrik Poinar) or the University of Alberta (PI: Duane Froese).

Specimen deposition

Permafrost cores remain in cold storage at the University of Alberta or McMaster University.

Dating methods

Plant macrofossils were picked from thawed samples using a dissecting microscope, dried and pre-treated for AMS dating at the University of Alberta along with known-age wood standards. Pre-treatment of all samples followed standard acid-base-acid procedures. Solutions heated to 70°C and placed in 1 M HCl for 30 minutes, followed by 60 minute washes in 1 M NaOH until the solution became clear. Finally, samples were placed in 1 M HCl for 30 minutes and rinsed with ultrapure water until they became neutral. Measurements of CO2 production, graphitization and radiocarbon abundance in all samples were completed at the Keck-Carbon Cycle AMS facility (UCIAMS). Details on age-modelling and a list of radiocarbon ages with 14C lab identifiers are included in the supplemental materials. Calibration/modelling was conducted with Oxcal v4.4 and the IntCal20 calibration curve. Raw, calibrated, and modelled ages are reported in the appendix in supplementary table 3.

☒ Tick this box to confirm that the raw and calibrated dates are available in the paper or in Supplementary Information.

Ethics oversight

No ethics approval was required. No palaentological or archaeological remains were processed in this study, only permafrost sediments collected in previous research efforts. No human sequence data was targeted or analyzed.

Note that full information on the approval of the study protocol must also be provided in the manuscript.
